# The Effectiveness of Psychological Interventions Delivered in Routine Practice: Systematic Review and Meta-analysis

**DOI:** 10.1007/s10488-022-01225-y

**Published:** 2022-10-06

**Authors:** Chris Gaskell, Melanie Simmonds-Buckley, Stephen Kellett, C. Stockton, Erin Somerville, Emily Rogerson, Jaime Delgadillo

**Affiliations:** 1 University of Sheffield, Sheffield, UK; 2 Sheffield Health and Social Care NHS Foundation Trust, Sheffield, UK

**Keywords:** Psychotherapy, Effectiveness, Naturalistic, Routine outcomes, Meta-analysis

## Abstract

This review presents a comprehensive evaluation of the effectiveness of routinely delivered psychological therapies across inpatient, outpatient and University-based clinics. This was a pre-registered systematic-review of studies meeting pre-specified inclusion criteria (CRD42020175235). Eligible studies were searched in three databases: MEDLINE, CINAHL and PsycInfo. Pre–post treatment (uncontrolled) effect sizes were calculated and pooled using random effects meta-analysis to generate effectiveness benchmarks. Moderator analyses were used to examine sources of heterogeneity in effect sizes. Overall, 252 studies (*k* = 298 samples) were identified, of which 223 (*k* = 263 samples) provided sufficient data for inclusion in meta-analysis. Results showed large pre–post treatment effects for depression [*d* = 0.96, (CI 0.88–1.04), *p* ≤ 0.001, *k* = 122], anxiety [*d* = 0.8 (CI 0.71–0.9), *p* ≤ 0.001, *k* = 69], and other outcomes [*d* = 1.01 (CI 0.93–1.09), *p* ≤ 0.001, *k* = 158]. This review provides support for the effectiveness of routinely delivered psychological therapy. Effectiveness benchmarks are supplied to support service evaluations across multiple settings.

**Supplementary Information:**

The online version contains supplementary material available at 10.1007/s10488-022-01225-y.

## Introduction

Meta-analyses of clinical trials support the efficacy of psychological interventions for various mental health problems such as depression (Cuijpers et al., 2008), anxiety disorders (e.g., Cuijpers et al., 2014a; Mayo-Wilson et al., 2014; Olatunji et al., 2014; Sánchez-Meca et al., 2010; Wolitzky-Taylor et al., 2008), post-traumatic stress disorder (Lewis et al., 2020), obsessive–compulsive disorder (Rosa-Alcázar et al., 2008), eating disorders (Linardon et al., 2017) and other conditions. Grounded in this evidence, clinical guidelines support the use of psychological interventions in routine clinical care (e.g., Chambless & Hollon, 1998; Chambless & Ollendick, 2001; National Institute for Health & Care Excellence, 2011). These guidelines commonly advocate the implementation of empirically supported treatments, closely following the procedures implemented in clinical trials and specified in associated treatment manuals. To this end, competency frameworks have been developed to support the dissemination of empirically supported treatments in routine care and clinical training programmes (e.g., Lemma et al., 2008; Roth & Pilling, 2008; Roth et al., 2009).

Some studies have found similar treatment outcomes when comparing data from efficacy trials and routine practice (e.g., Lutz et al., 2016; Persons et al., 1999). However, there are some reasons to assume that the effects of psychotherapy delivered in routine care settings may differ from those observed in clinical trials. Recent evidence indicates that psychological treatment outcomes are associated with treatment *integrity*, which refers to the competent (skilled) delivery of protocol-driven treatment procedures (Power et al., 2022). However, surveys of clinicians working in routine settings often reveal negative attitudes towards protocol-driven treatment and a lack of adherence to treatment manuals (e.g., Addis & Krasnow, 2000). Hence, the integrity of routinely delivered psychological treatments is unclear, and it probably varies across services (Freedland et al., 2011). Furthermore, the strict selection criteria applied in clinical trials may result in unusually homogeneous samples that do not reflect the diverse clinical populations typical of routine care settings (e.g., Lambert, 2013; Zimmerman et al., 2002). Previous studies have found systematic differences in the clinical profiles of patients included and excluded from psychotherapy trials (e.g., van der Lem et al., 2012). For these reasons, it is plausible to assume that the effects of routinely delivered therapy may vary across settings and clinical populations, and may not necessarily conform to benchmarks from efficacy trials.

A tradition of practice-based evidence (PBE, Margison et al., 2000) has emerged in recent decades, with numerous studies examining the effects of routinely delivered psychological interventions in various settings. Narrative reviews of PBE generally confirm that moderate-to-large uncontrolled (pre-to-post treatment) effect sizes are observed in routine care settings, supporting the effectiveness of psychotherapy but also demonstrating considerable variability across patient samples, therapists and clinics (e.g., see Barkham et al., 2010; Castonguay et al., 2013, 2021). An inherent limitation of such narrative reviews is that they perform a selective rather than systematic synthesis of available data. Benchmarking studies can be useful to provide general indices of treatment effectiveness, enabling services to evaluate their outcomes relative to efficacy trials (e.g., McAleavey et al., 2019; Minami et al., 2008) or aggregated effect size data from similar clinical services (e.g., Delgadillo et al., 2014). Psychotherapy benchmarking studies tend to report favorable pooled effects sizes, but also show variability in effects across clinics (e.g., Barkham et al., 2001; Connell et al., 2007; Delgadillo et al., 2014; Gyani et al., 2013). Although benchmarking studies help to quantify the expected magnitude of treatment effects observed in routine clinical settings, most are nevertheless circumscribed to small sets of clinics or geographical areas, offering limited insights into possible sources of heterogeneity in treatment outcomes. Systematic reviews and meta-analyses may therefore offer a more comprehensive examination of the effectiveness of routinely delivered treatments.

Some meta-analytic investigations have reported that outcomes from routine practice-based treatments are not as favorable as those delivered in research settings (Weisz et al., 1995). Other meta-analyses suggest that there are no differences in treatment effects when comparing PBE and efficacy studies after controlling for case-mix differences (e.g., Shadish et al., 1997, 2000). However, many of the PBE studies in these meta-analyses applied stringent controls on the treatment procedures (e.g., adherence and competence assessments)—making them more akin to efficacy trials. Hunsley and Lee (2007) reviewed 35 studies and concluded that the completion and improvement rates observed in PBE studies were comparable to efficacy trials. Cahill et al. (2010) reviewed 31 studies, concluding that psychotherapy was most effective for the treatment of common mental disorders, with a pooled uncontrolled effect size of *d* = 1.29. More recently, Wakefield et al. (2021) reviewed 60 studies, of which 47 were eligible for meta-analysis. They reported large uncontrolled effect sizes for depression (*d* = 0.87) and anxiety (*d* = 0.88), and a moderate effect on functional impairment (*d* = 0.55). These meta-analyses show wide variability in treatment effects (i.e., heterogeneity) across studies/samples.

PBE meta-analyses provide some insights into plausible sources of heterogeneity, including methodological (e.g., completers analyses vs. inclusion of patients lost to follow-up) and clinical features (e.g., larger effects for common mental disorders, lower effects for patients with comorbidities and socioeconomic disadvantages, larger effects for lengthier interventions). Nevertheless, these meta-analyses are over a decade old (Cahill et al., 2010; Hunsley & Lee, 2007) or limited to a specific treatment setting (e.g., primary care outpatient services; Wakefield et al., 2021). Further research into the methodological and clinical sources of treatment heterogeneity is needed to better understand why treatment effects vary across samples, and to determine whether or not these effects vary across different treatment settings (e.g., outpatient, inpatient, university-based treatment).

The considerable growth of the PBE literature in the last decade and Implementation of empirically supported treatments across many settings warrants a comprehensive review of treatment outcomes data. The aim of the present study was to systematically review available PBE studies. The objectives of the study were to (1) provide benchmarks of treatment effectiveness using meta-analysis and (2) to examine sources of effect size heterogeneity using pre-specified moderator analyses informed by earlier studies.

## Methods

### Search Strategy and Study Selection

The present study followed good practice guidelines for systematic reviews (PRISMA, Page et al., 2021) and meta-analyses of psychotherapy studies (MAP-24, Flückiger et al., 2018). A review protocol was pre-registered in the PROSPERO database (CRD42020175235).^1^ Literature searches were carried out without any restrictions on date of publication up to the search date (April 2020). Inclusion criteria were: (a) studies reporting outcomes for routinely delivered treatments (i.e., not as part of efficacy trials); (b) all adult sample (no patients under 16); (c) employed a *psychological treatment* (i.e., driven by psychological theory and intended to be therapeutic (Spielmans & Flückiger, 2018), as inferred or described by study manuscripts); and (d) conducted face-to-face. Studies were excluded if they: used (e) family/group treatments, (f) were not available in English; (g) did not employ a self-report measure of treatment effectiveness^2^; (h) did not provide sufficient data to calculate pre–post treatment effect sizes; or (i) employed randomization procedures or control groups. A more detailed table of inclusion/exclusion criteria is available in supplementary Table 1.

The search strategy had three phases. Phase one was a systematic search of three electronic literature databases (MEDLINE, CINAHL and PsycInfo) via EBSCO using a pre-registered list of key terms. Methodological terms included: *practice-based evidence*, *routine practice*, *benchmarking*, *transportability*, *transferability*, *clinically representative*, *managed care setting*, *uncontrolled*, *external validity*, *applicable findings*, *empirically supported*, *dissemination*, and *clinical effectiveness evaluation*. These terms were informed by prior reviews of psychotherapy effectiveness (Cahill et al., 2010; Stewart & Chambless, 2009). *Effectiveness* and *evaluation* were not used as single word terms due to producing unmanageable numbers of irrelevant records. For the psychologically relevant term: psycho* OR therap* was used for PsycInfo while psycho* alone was used for MEDLINE and CINAHL (therap* was removed from MEDLINE/CINAHL due to producing an unmanageable number of irrelevant records). Limiters included *adult population* and *English language*. No exclusions were made based on the type of publication. Key term combinations and Boolean operators are reported in supplementary Table 2. Phase two included a manual search of reference lists, and forward citation searching (using Google Scholar) for studies identified in phase one. Titles relevant to the current review were identified by the first author. Finally, phase three was a grey literature search using the terms *psychotherapy* AND *routine-practice* AND *effectiveness* in Google Scholar.

After removal of duplicates, titles and abstracts of potentially eligible studies were screened by the first author using a pre-developed and piloted screening tool. Sub-samples were screened by a second coder at each stage (20% at the stage of title screening; 10% at the stage of full-text screening). Percentage agreement and inter-rater reliability statistics (Kappa ($\kappa$), Cohen, 1960] indicated good reliability ($\kappa$ = 0.78, 1713/1740, 98.45%) in the first stage and adequate reliability ($\kappa$ = 0.65, 24/30, 80%) in the second stage. After the selection process was completed, corresponding authors for eligible studies were contacted via email to request additional recommendations for potentially eligible studies, and to request additional statistical information to calculate effect sizes. E-mail responses were received from 76 authors and additional data was provided for 41 samples.

### Data Extraction

There were three separate outcome domains (and subsequently three meta-analyses) for ‘depression’, ‘anxiety’ and ‘other’ outcomes. The latter category consisted of general psychological distress scales, measures of functioning/quality of life, or diagnosis-specific outcome scales (e.g., obsessive-compulsive disorder, etc.). A pilot extraction sheet was developed and pilot-tested with a sample of studies (*k* = 10). When multiple samples were reported in the same study, effect-sizes across these samples were aggregated to reduce bias of statistical dependency (Gleser & Olkin, 2009; Hoyt & Del Re, 2018). To avoid loss of information (e.g., aggregating sub-samples that are distinct based on levels of a moderator), study samples were disaggregated for moderator analyses (Cooper, 1998). Studies with overlapping datasets (e.g., reanalysis of the same sample) were only included once in the meta-analysis. Samples which performed an intention-to-treat (ITT) analysis were preferred to completer samples due to being less prone to attrition bias (Jüni et al., 2001); so the ITT data was extracted for studies that reported both ITT and completer analyses. As extraction of multiple study effect-sizes within a single domain (e.g., depression) threatens statistical dependency (Borenstein et al., 2021) we selected a single effect-size per domain (Card, 2015; Cuijpers, 2016), using a preference system (defined a priori, supplementary material). Reliability of coding for effect-size data was computed using a second coder for a sub-sample of manuscripts (n = 29) demonstrating almost perfect reliability across all values ($\kappa$ = 0.97, agreement = 97.56%) and perfect reliability for effect-size values ($\kappa$ = 1.00). Key categorical and numerical variables extracted from manuscripts for moderator analyses are reported in Table 1. For sample severity, the decision was made to cluster university counselling centers in the ‘mild’ severity category due to prior research finding normative data of UK University students comparable to primary care samples (Connell et al., 2007).

**Table 1:** Summary coding sheet for extracting study information

*Categorical variables*
Setting: the study was (i) *out-patient*, (ii) *inpatient* or (iii) *mixed*
Analysis: samples (i) *included* or (ii) *excluded* (completers) patients lost to follow up
Severity: was determined through a stratification of studies based on characteristics of the service (similar to the approach used by de Jong et al., 2021). (i) *Mild services* included primary care, physical health, university counselling, voluntary, private (independent or group) and employee assistance programmes; (ii) *Moderate services* included secondary care, community mental health centers, specialist psychotherapy centers, managed care settings, or intensive outpatient programmes; (iii) *severe services* represented inpatient samples; and (iv) *university* included university outpatient and training clinics (which are known to vary in the severity of sample)
Treatment modality: Treatments were coded as (i) *cognitive-behavioral* or (ii) *psychodynamic* based on manuscript self-designation (i.e., if the manuscript described treatment as CBT, then that was coded). In the absence of these terms, modality of best-fit was decided using treatment descriptions. Treatments that could not be confidently allocated to these groups were coded as (iii) *counselling* (e.g., person-centred, undefined) or (iv) *other*. Treatments that did not describe treatment modality were rated as other
Continent: Studies were coded as North America, United Kingdom (UK), mainland Europe, Australasia, or Asia. The UK was separated from Europe because of the high representation of outcomes research coming from the UK
Intervention development stage: Studies were coded as (i) *preliminary studies* (i.e., testing novel treatments or treatment iterations) or (ii) *routine evaluations*
Experience: Samples for which treatment delivery was exclusively by (i) *trainees*, or (ii) *qualified professionals*
Measurement tool: Measures that were represented at least ten times in the meta-analysis were entered as subgroups
Sample Size: Following the approach of Barth et al. (2013), studies were coded as small (N ≤ 25), medium (N = 25–50), or large (N = 50+)
*Continuous variables*
Age: Sample mean average age
Year: of publication
Female participants: Sample rate (%)

These moderators form the subgroup and continuous variables moderator variables for the current study

### Risk of Bias and Quality Assessment

The Joanna Briggs Institute Quality Appraisal Tool for Case Series (Munn et al., 2020) was used to assess risk of bias. Eight criteria primarily focusing upon manuscript reporting detail were used. Criteria included manuscript reporting of: (i) patient inclusion criteria, (ii) service description, (iii) treatment description, (iv) sample characteristics, (v) outcome data, (vi) effect-size calculation, (vii) consecutive patient recruitment, and (viii) inclusion of patients lost to follow-up in statistical analysis. Each item was coded as either met or not met (including not clear) by the first author for each sample. A sub-sample (23.8%) was rated independently by two other reviewers (11.9% each). The pooled agreement was 84.17% ($\kappa$ = 0.62).

### Statistical Analysis

All analyses were conducted using the R statistical analysis environment (R Core Team, 2020, v 4.0.2). We calculated standardised mean change (SMC: Becker, 1988) for included studies using the metafor package. This approach divides the pre–post mean change score by the pretreatment standard deviation with a sampling variance adjustment using the correlation between the pre-treatment and post-treatment measures (Morris, 2008). When unavailable, Pearson’s *r* was imputed using an empirically derived estimate (*r* = .60, Balk et al., 2012). Aggregation of samples/sampling errors was conducted using the *aggregate* function of *metafor* using standard inverse-variance weighting.

Random effects meta-analyses were performed using the *metafor* (Viechtbauer, 2020), *dmetar* (Harrer et al., 2019), and *meta* (Schwarzer, 2020) packages. Forest plots were used to visualise pre–post treatment effects sizes across samples. Effect size heterogeneity was assessed using I^2^ (Higgins & Thompson, 2002) and the Q statistic (Cochran, 1954). Publication bias was examined using funnel plots and assessed statistically using rank correlation tests (Begg & Mazumdar, 1994), Egger’s regression test for funnel plot asymmetry (Egger et al., 1997), and the fail-safe N (Rosenthal method, Rosenthal, 1979).

Moderator analyses were based on a set of moderator variables selected a priori, following evidence from prior reviews. Subgroup variables included: (i) *analysis* (inclusion of patients lost to follow-up), (ii) *geographical region*, (iii) *severity* (mild, moderate, severe, university^3^), (iv) *treatment modality*, (v) *experience* [unqualified (i.e., trainees) vs. qualified therapists], (vi) *stage of treatment development* (preliminary study vs. routine evaluations), and (vii) *sample size* (small, medium, large). Continuous meta-regression variables included (i) *publication year*, (ii) average *age* of sample, and (iii) percentage of samples who identified as *female*. All moderators were included in meta-regression which was based on a mixed effects (i.e., multilevel) model (Borenstein et al., 2021) with weighted estimation (inverse-variance weights).

Finally, we developed effect size benchmarks to support the evaluation of effectiveness across four broad settings: outpatient services, inpatient services and university counselling services (i.e., student population) and university psychotherapy clinics (non-student population). Informed by previous benchmarking studies (Delgadillo et al., 2014), pooled effect sizes (using random effects meta-analyses) were stratified into quartiles to differentiate between low effectiveness (bottom 25%), average effectiveness (middle 50%) and high effectiveness benchmarks (top 25%).

## Results

### Search Results

**Fig. 1:**
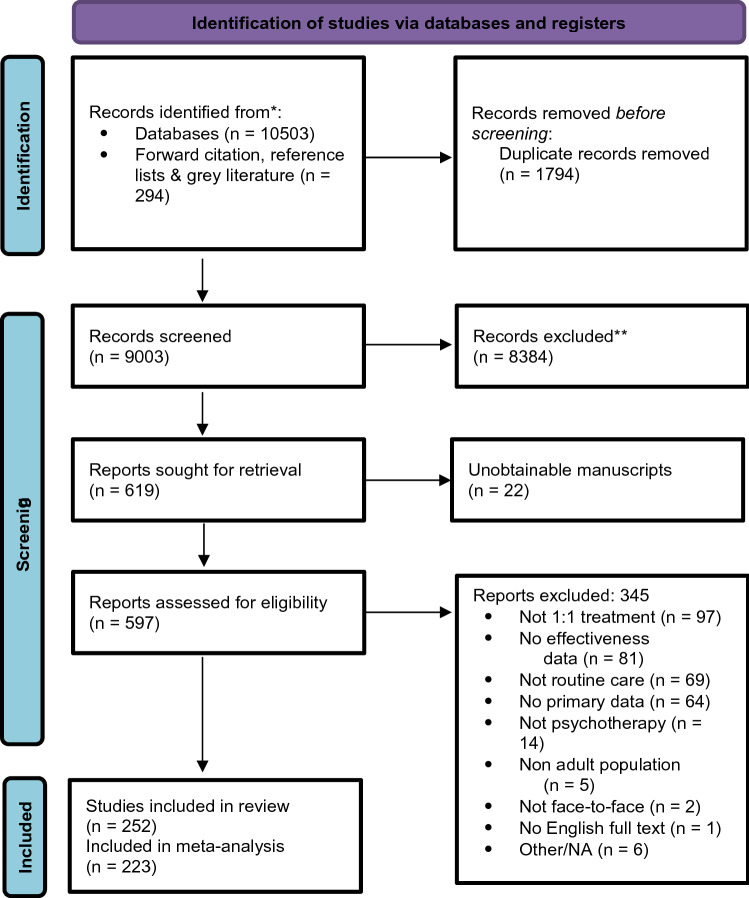
Prisma flow diagram of studies throughout the review

The PRISMA diagram in Fig. 1 presents a summary of the study selection process. Overall, 10,503 records were identified, of which 252 manuscripts were eligible for inclusion and 223 (samples *k* = 263) had sufficient information to be included in the meta-analysis. Summary statistics are provided in Table 2.

**Table 2:** Summary statistics across the pooled sample and by sample severity

	University	Mild	Moderate	Severe	Other	Total
N						
N	9195	158,150	9515	22,586	33,694	233,140
*k*	58	88	32	92	8	278
Mean	158.53	1797.16	297.34	245.50	4211.75	838.63
Median	93.50	121.00	61.00	63.00	93.00	81.50
IQR	162.50	935.00	347.00	107.50	1999.75	224.50
Females						
N	5350	95,373	5797	14,952	22,801	144,273
Age						
*k*	65.000	77.00	29	82	7	260
Mean	33.78	36.53	34.80	35.55	36.24	35.33
Min	20.50	19.00	24.30	21.52	24.52	19.00
Max	52.29	60.50	47.49	52.00	46.10	60.50
Sessions						
*k*	54	64	4	54	6	182
Mean	21.00	11.26	13.75	14.67	8.55	15.13
Min	2.15	4.00	9.00	1.00	8.00	1.00
Max	85.33	64.90	24.00	64.00	9.52	85.33
Median	14.77	8.18	11.00	11.15	8.15	13.00
IQR	13.55	8.60	3.750	10.00	1.20	9.98
Setting						
Mixed	0	0	0	0	5	5
Outpatient	68	96	0	91	4	259
Inpatient	0	0	33	1	0	34
Continent						
Asia	4	1	0	0	1	6
Australasia	5	0	0	5	0	10
Europe	20	13	15	14	1	63
America	38	32	10	39	4	123
UK	1	50	8	34	3	96
Analysis						
Inclusion	48	48	16	53	4	169
Completers	19	45	16	35	3	118
Therapy modality						
Cognitive-behavioural	43	41	14	49	5	152
Counselling	0	22	0	3	0	25
Psycho-dynamic	12	9	13	16	0	50
Other	13	24	6	24	4	71
Treatment stage						
Preliminary	4	6	7	16	1	34
Evaluations	64	90	26	76	8	264

### Study Characteristics

Eligible studies were published between 1984 and 2020 (median = 2013, *k* = 294 published ≥ 2000). Of these, 169 samples included patients lost to follow-up (*k* = 118, 56.72% completers). Most studies were from the USA (*k* = 113, 37.92%), England (*k* = 78, 26.17%), Germany (*k* = 24, 8.05%), Sweden (*k* = 12, 4.02%) and Canada (*k* = 10, 3.36%). These five most represented countries accounted for most of the included samples (*k* = 237, 79.53%).

### Sample Characteristics

Sample characteristics were reported for 291 samples, with a cumulative N of 233,140 patients (mean = 838.63, median = 81.5, range—4 to 33,243, IQR = 224.5). The prevalence of female participants was 61.88% (N = 144,273, *k* = 279) with 13 all-female samples and 2 all-male samples. The mean average sample age was 35.33 years (range = 19.00–60.50). Across studies which provided information, 23.00% of patients were from ethnic minorities (*k* = 127), 37.00% were married (*k* = 106), and 23.00% were in employment (*k* = 96).

### Treatment Characteristics

Most samples evaluated cognitive-behavioral interventions (*k* = 152, 51.01%) while 50 samples evaluated psychodynamic (16.78%), and 25 samples evaluated counselling (8.29%; other = 71, 23.82%). Counselling interventions were interventions described simply as ‘counselling’ by study authors (with no further treatment information) or ‘person-centered counselling’ interventions. Interventions termed ‘counselling’ but described in a way that fit closely with of the other treatment modalities (e.g., cognitive-behavioral counselling) was assigned to the more specific treatment modality group. For symptom severity, 96 (32.21%) samples came from services treating mild conditions, 92 (30.87%) from services treating moderate conditions, 33 (11.07%) from services treating severe conditions, and 68 (22.82%) from university psychotherapy clinics (not counselling centers) that treated a wide spectrum of conditions from mild-to-severe (other, *k* = 9, 3.02%). Treatment dosage, when reported (*k* = 256) was in hours/sessions (*k* = 225), months (*k* = 12) or days (*k* = 8). The pooled (non-weighted) average dose (hours) was 16.30 sessions (median = 13.00, range = 1.00–139.30, IQR = 11.00). A total of 62 (20.81%) samples reported that treatment was delivered exclusively by trainees, while 100 (35.58%) samples reported having at least one trainee.

### Risk of Bias

In order of satisfactory criteria (e.g., the criterion under evaluation was met), the following risk of bias domains were assessed: demographic reporting detail (264/298, agreement = 98.33%, $\kappa$ = 0.88), service reporting detail (260/298, agreement = 85%, $\kappa$ = 0.31), study outcome reporting details (240/298, agreement = 83.33%, $\kappa$ = − 0.03), intervention reporting detail (234/298, agreement = 85%, $\kappa$ = 0.32), service inclusion criteria (214/298, agreement = 90%, $\kappa$ = 0.64), appropriate use of analysis (214/298, agreement = 70%, $\kappa$ = 0.26), complete inclusion (i.e. consecutive recruitment and inclusion of those lost to follow-up, 41/298, agreement = 85%, $\kappa$ = 0.45), and consecutive inclusion (93/298, agreement = 76.67%, $\kappa$ = 0.51).

### Meta-analyses

**Fig. 2:**
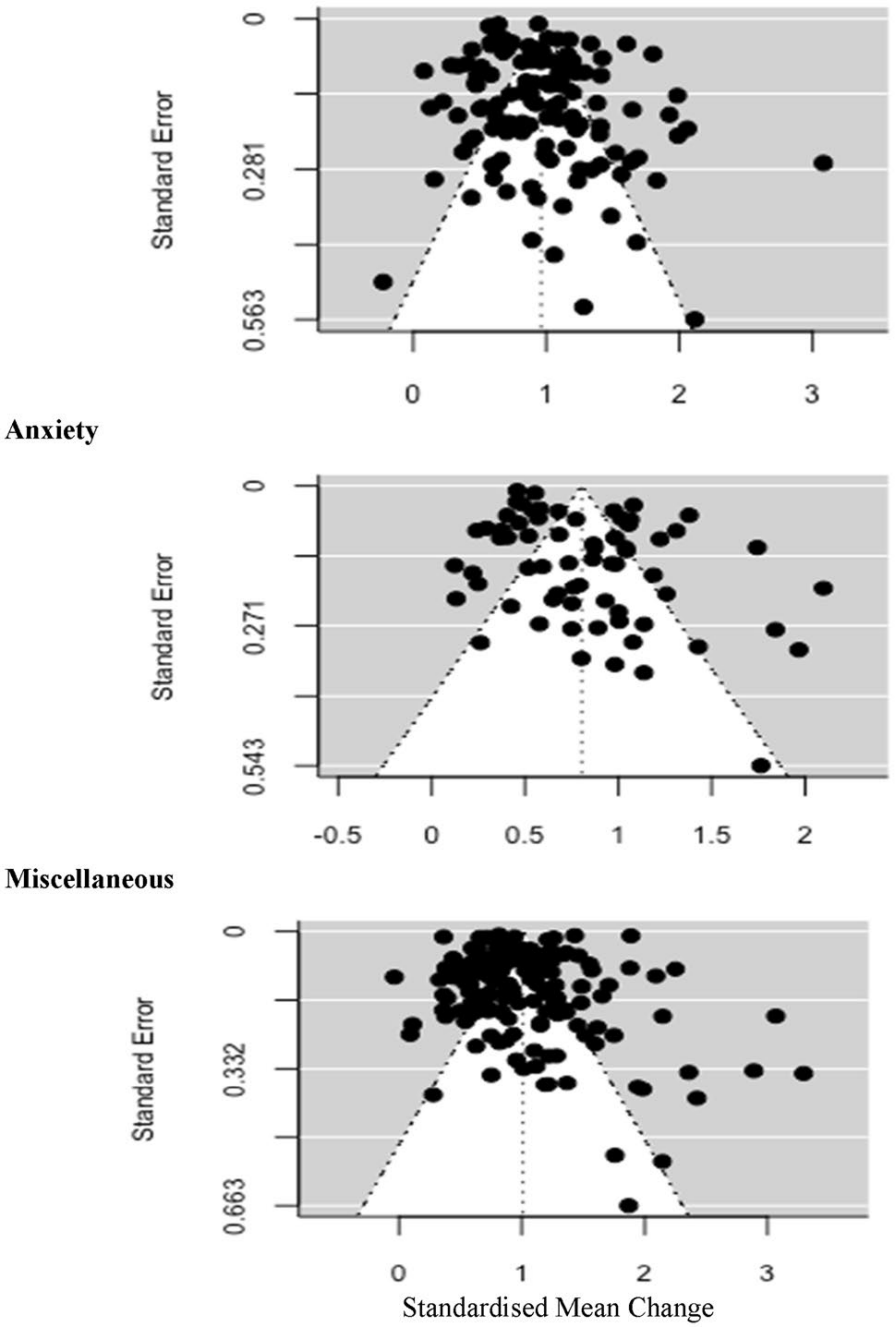
Funnel plots displaying the distribution of studies reporting pre–post outcomes for (i) depression, (ii) anxiety, and (iii) miscellaneous outcomes

The random-effects meta-analysis for depression outcomes (*k* = 140, N = 68,077), across 10 unique measurement tools was statistically significant (*p* ≤ 0.001), indicative of a large pre–post treatment (*d* = 0.96, CI 0.88–1.04) reduction in depression severity. There was a large magnitude of statistically significant heterogeneity [I^2^ = 97.94%, Q(df = 121) = 2677.37, *p* ≤ 0.001]. The funnel plot (Fig. 2) shows limited visual evidence of asymmetry. The funnel rank correlation test was not statistically significant ($\tau$ = 0.061, *p* = 0.46) however the funnel regression test was statistically significant (Z = 2.13, *p* = 0.033). The fail-safe N was 515,853.

The random-effects meta-analysis for anxiety outcomes (*k* = 84, N = 26,689, measurement tools = 20) was statistically significant (*p* ≤ 0.001), indicative of a large (*d* = 0.80, CI 0.71–0.90) reduction in symptom severity. Heterogeneity was large and statistically significant [I^2^ = 97.51%, Q(df = 68) = 1328.96, *p* ≤ 0.001]. The funnel plot shows limited evidence of asymmetry. The funnel rank correlation test was not significant ($\tau$ = 0.009, *p* = 0.888). In contrast, the funnel regression test was statistically significant (Z = 2.533, *p* = 0.011). The fail-safe N was 121,899.

The random-effects meta-analysis for other outcomes (*k* = 184, N = 126,734, measurement tools = 40) was statistically significant (*p* ≤ 0.001), indicative of a large (*d* = 1.01, CI 0.93–1.09) reduction in severity of indices of distress. Heterogeneity was large and statistically significant [I^2^ = 99.06%, Q(df = 157) = 15,330.32, *p* ≤ 0.001]. The funnel plot shows a degree of asymmetry with clustering to the right of the mid-line. The funnel rank correlation test was statistically significant ($\tau$ = 0.208, *p* ≤ 0.001). In contrast, the funnel regression test was not significant (Z = 3.697, *p* ≤ 0.001). The fail-safe N was 1,695,607.

### Moderator Analyses

Multivariable meta-regressions were conducted for each of the three outcome domains (Tables 3, 4, 5). After controlling for other moderators, the depression meta-regression found a significant effect for geographical region, therapist experience and type of analysis. UK samples had larger effect sizes compared to samples from Asia; effects sizes in samples treated by qualified staff members were larger than those observed in samples exclusively consisting of trainees; and samples excluding patients lost to follow-up (i.e., completer analyses) had larger effect sizes compared to intention-to-treat analyses. For anxiety outcomes, UK studies had larger effect sizes than studies from mainland Europe; mild severity samples had larger effect sizes than samples of patients with moderate or severe symptoms; and cognitive-behavioural interventions had larger effect sizes than counselling interventions. Finally, for other outcomes, the only significant moderator indicated that cognitive-behavioural interventions had larger effect sizes than psychodynamic interventions and unspecified (i.e., other) interventions.

**Table 3:** Multi-moderator analyses for depression outcomes

	Moderator level	*d*	SE	Z	P	CI
*Intercept*		1.22	0.17	7.20	< .0001	0.88 to 1.56
Region	UK	Ref				
	North America	− 0.04	0.10	− 0.34	0.733	− 0.24 to 0.17
	Mainland Europe	− 0.25	0.13	− 1.94	0.053	− 0.50 to 0.00
	Asia	− 0.62	0.24	− 2.62	0.001*	− 1.09 to − 0.16
	Australasia	− 0.49	0.26	− 1.87	0.062	− 1.01 to 0.02
Severity	Mild	Ref				
	Moderate	− 0.13	0.11	− 1.17	0.241	− 0.34 to 0.09
	Severe	− 0.10	0.15	− 0.70	0.482	− 0.39 to 0.18
	University (mild-to-severe)	0.20	0.15	1.34	0.180	− 0.01 to 0.93
Therapy modality	Cognitive-behavioural	Ref				
	Psychodynamic	0.07	0.11	0.62	0.540	− 0.15 to 0.28
	Counselling	− 0.27	0.23	− 1.19	0.236	− 0.71 to 0.18
	Other	− 0.04	0.12	− 0.33	0.742	− 0.28 to 0.12
Treatment stage	Preliminary studies	Ref				
	Routine evaluations	− 0.12	0.13	− 0.99	0.324	− 0.37 to 0.12
Analysis	Includes lost to follow up	Ref				
	Completers	0.16	0.13	2.01	0.045*	0.00 to 0.32
Experience	Qualified staff	Ref				
	Trainees	− 0.29	0.14	− 2.06	0.039*	− 0.57 to − 0.01
Sample size	Large	Ref				
	Medium	− 0.15	0.10	− 1.44	0.151	− 0.35 to 0.05
	Small	− 0.21	0.11	− 1.83	0.069	− 0.43 to 0.02
Publication year		− 0.001	0.01	− 0.19	0.851	− 0.01 to 0.01
Sample age		− 0.004	0.01	− 0.67	0.503	− 0.02 to 0.01
% Female		0.13	0.23	0.56	0.574	− 0.33 to 0.59

k = 124, Tau^2^ = 0.17[SE = 0.02], I^2^ = 99.99%, R^2^ = 19.28%

* = < .05

**Table 4:** Multi-moderator analyses for anxiety outcomes

	Moderator level	*d*	SE	Z	P	CI
*Intercept*		1.24	0.22	5.59	< .0001	0.80 to 1.67
Region	UK	Ref				
	North America	− 0.13	− 0.13	− 0.91	0.363	− 0.40 to 0.14
	Mainland Europe	− 0.35	0.15	− 2.37	0.018*	− 0.63 to − 0.06
	Asia	− 0.55	0.29	− 1.87	0.061	− 1.13 to 0.026
	Australasia	− 0.32	0.24	− 1.30	0.194	− 0.79 to 0.16
Severity	Mild	Ref				
	Moderate	− 0.41	0.15	− 2.71	0.007*	− 0.70 to − 0.11
	Severe	− 0.49	0.19	− 2.56	0.011*	− 0.86 to 0.11
	University (mild-to-severe)	0.03	0.17	0.20	0.838	− 1.21 to 0.45
Therapy modality	Cognitive-behavioural	Ref				
	Psychodynamic	0.00	0.14	0.01	0.989	− 0.27 to 0.28
	Counselling	− 0.64	0.30	− 2.16	0.031*	− 1.23 to − 0.06
	Other	− 0.64	0.16	− 0.41	0.368	− 0.39 to 0.25
Treatment stage	Preliminary studies	Ref				
	Routine evaluations	− 0.13	0.16	− 0.81	0.421	− 0.45 to 0.19
Analysis	Includes lost to follow up	Ref				
	Completers	0.15	0.12	1.28	0.120	− 0.08 to 0.38
Experience	Qualified staff	Ref				
	Trainees	0.08	0.16	0.50	0.614	− 0.23 to 0.39
Sample size	Large	Ref				
	Medium	0.15	0.13	1.11	0.267	− 0.11 to 0.40
	Small	− 0.01	0.12	− 0.07	0.942	− 0.23 to 0.22
Publication year		0.01	0.01	1.71	0.088	− 0.00 to 0.03
Sample age		− 0.01	0.01	− 1.16	0.248	− 0.03 to 0.01
% Female		− 0.22	0.37	− 0.59	0.555	− 0.94 to 0.50

k = 78, Tau^2^ = 0.13[SE = 0.02], I^2^ = 99.95%, R^2^ = 40.55%

* = < .05

**Table 5:** Multi-moderator analyses for other outcomes

	Moderator level	*d*	SE	Z	P	CI
*Intercept*		1.13	0.17	6.60	< .0001	0.80 to 1.47
Region	UK	Ref				
	North America	0.17	0.11	1.59	0.111	− 0.04 to 0.39
	Mainland Europe	0.04	0.12	0.32	0.752	− 0.20 to 0.27
	Asia	0.03	0.27	0.10	0.924	− 0.50 to 0.55
	Australasia	− 0.16	0.31	− 0.49	0.626	− 0.76 to 0.46
Severity	Mild	Ref				
	Moderate	− 0.14	0.11	− 1.23	0.220	− 0.36 to 0.08
	Severe	− 0.21	0.14	− 0.12	0.901	− 0.30 to 0.26
	University (mild-to-severe)	0.49	0.17	− 1.25	0.210	− 0.54 to 0.12
Therapy modality	Cognitive-behavioural	Ref				
	Psychodynamic	− 0.25	0.11	− 2.23	0.026*	− 0.47 to − 0.03
	Counselling	− 0.16	0.18	− 0.86	0.387	− 0.51 to 0.12
	Other	− 0.39	0.11	− 3.47	0.001*	− 0.60 to − 0.17
Treatment stage	Preliminary studies	Ref				
	Routine evaluations	0.06	0.13	0.45	0.650	0.20 to 0.32
Analysis	Includes lost to follow up	Ref				
	Completers	0.14	0.09	1.59	0.111	− 0.03 to 0.31
Experience	Qualified staff	Ref				
	Trainees	− 0.30	0.16	− 1.90	0.058	− 0.61 to 0.01
Sample size	Large	Ref				
	Medium	− 0.01	0.12	− 0.09	0.925	− 0.24 to 0.22
	Small	− 0.06	0.12	− 0.49	0.626	− 0.30 to 0.18
Publication year		0.00	0.01	0.46	0.646	− 0.01 to 0.02
Sample age		− 0.00	0.01	− 0.56	0.576	− 0.02 to 0.01
% Female		− 0.14	0.23	− 0.62	0.534	− 0.59 to 0.31

k = 153, Tau^2^ = 0.24[SE = 0.03], I^2^ = 100%, R^2^ = 21.44%

* = < .05

### Benchmarking Data

Pooled effect-sizes for low, average and high performing services are shown in Table 6, organized according to setting [outpatient services, inpatient services, university counselling services (i.e., student population) and university psychotherapy clinics (non-student population)]. Although the effect size estimates for each benchmark vary across settings, confidence intervals consistently overlapped, indicating similar levels of symptom-changes across the performance strata (low, average, high). The exception to this is the low performance benchmark for anxiety measures which were significantly larger in university psychotherapy clinics (*d* = 0.51) and significantly smaller in inpatient services (*d* = 0.13) by comparison to outpatient services (*d* = 0.37).

**Table 6:** Benchmarks for routine services based on individual study sample quartiles

	Outpatient	Inpatient	UCC	Uni clinics
Top 25%				
Depression	*d* = 1.68 [1.53–1.83]	*d* = 1.34 [1.16–1.52]	*	*d* = 1.77 [1.50–2.03]
Anxiety	*d* = 1.56 [1.38–1.73]	*d* = 1.07 [1.04–1.09]	*	*d* = 1.80 [1.57–2.02]
Other	*d* = 1.70 [1.54–1.86]	*d* = 1.67 [1.37–1.97]	*d* = 1.47 [1.24–1.69]	*d* = 1.14 [1.10–1.18]
Average				
Depression	*d* = 0.94 [0.90–0.97]	*d* = 0.98 [0.81–1.15]	*	*d* = 0.91 [0.87–0.95]
Anxiety	*d* = 0.84 [0.78–0.89]	*d* = 0.67 [0.42–0.92]	*	*d* = 0.95 [0.87–1.02]
Other	*d* = 0.92 [0.89–0.96]	*d* = 1.04 [0.96–1.11]	*d* = 0.94 [0.84–1.03]	*d* = 0.86 [0.77–0.94]
Low 25%				
Depression	*d* = 0.46 [0.41–0.52]	*d* = 0.38 [0.26–0.5]	*	*d* = 0.40 [0.27–0.54]
Anxiety	*d* = 0.37 [0.33–0.42]	*d* = 0.13 [0.03–0.29]	*	*d* = 0.51 [0.44–0.57]
Other	*d* = 0.49 [0.43–0.54]	*d* = 0.58 [0.46–0.69]	*d* = 0.64 [0.61–0.67]	*d* = 0.41 [0.23–0.59]

University clinics refers to university managed clinics treating communities beyond the student population. University counselling centres that are more specifically targeted at the student population are included within the mild category

*UCC* University Counselling Centres; *d* uncontrolled, pre-to-post treatment effect size [95% confidence intervals]

*Cannot be computed due to too few samples

## Discussion

This review provides a comprehensive quantitative review of the effectiveness of psychological treatments delivered in routine care settings. Overall, 252 studies (samples *k* = 298) were identified, of which 223 (88.5%, *k* = 263) were included in the meta-analysis. Consistent with prior psychotherapy effectiveness reviews, we found large uncontrolled (pre–post treatment) effect sizes (*d* = 0.80–1.01) across multiple outcome domains (depression, anxiety, and general psychological distress).

Consistent with previous meta-analyses of PBE (e.g., Cahill et al., 2010; Hunsley & Lee, 2007; Wakefield et al., 2021), we observed wide variability in effect sizes across studies and large (> 90%) indices of heterogeneity across outcome domains. The large number of samples included in this review enabled us to carry out adequately-powered moderator analyses to better understand potential sources of heterogeneity. For depression outcomes, smaller effect sizes were found for samples in Asia (compared to the UK), and in treatments delivered by trainees (i.e., compared to qualified professionals). For anxiety outcomes, smaller effect sizes were found for treatments delivered in mainland Europe (compared to the UK), services treating patients with moderate or high levels of severity (compared to mild severity), and counselling interventions (compared to cognitive-behavioural interventions). For other outcomes, only therapy modality was significant. Psychodynamic and unspecified interventions produced smaller effect-sizes (compared to cognitive-behavioural interventions). To some extent, these results are consistent with and support clinical guidelines that recommend cognitive-behavioural therapy as a first-line intervention, prior to considering other treatment modalities (National Institute for Health & care Excellence, 2011). However, caution is advised when interpreting these between-therapy comparisons using uncontrolled data from observational studies, as they could be explained by other unmeasured factors such as relevant case-mix differences between patients (e.g., socioeconomic status, personality, comorbid physical illnesses, etc.). Studies that control for case-mix variables using individual patient data find that there are no significant differences in treatment effects when comparing different treatment modalities (e.g., Pybis et al., 2017). Furthermore, as found in a previous meta-analysis (Wakefield et al., 2021), completers analyses tended to produce inflated (biased) effect sizes by comparison to intention-to-treat (more conservative and stringent) analyses.

The finding of large clinical improvements during psychotherapy and across outcomes was consistent with prior meta-analyses of psychotherapy effectiveness for depression outcomes (Hans & Hiller, 2013; Wakefield et al., 2021), anxiety outcomes (Stewart & Chambless, 2009; Wakefield et al., 2021), and other indices of psychological distress and functioning (Cahill et al., 2010). Pooled uncontrolled effect-sizes were smaller than that reported by Cahill et al. (2010) (*d* = 1.29), although this may reflect differences in the focus of the reviews (e.g., Cahill et al., 2010 included group treatments) or the changing distribution of geographical representation (i.e., more studies from non-UK/North American countries). Large clinical improvements are also consistent with many meta-analyses of psychotherapy controlled trials (e.g., Cuijpers et al., 2008, 2014a; Mayo-Wilson et al., 2014; Olatunji et al., 2014).

It is possible that there are continental differences in models of training, service structures, therapy provision and emphasis on evidence-based practice which underlie the observed differences in pooled effect-sizes between continents. This is consistent with UK and US clinical guidance recommending delivery of empirically supported treatments (APA, 2006; National Institute for Health and Care Excellence, 2011). It is possible that the service policy context in the UK places greater emphasis on the delivery of treatment with high fidelity to empirically supported treatment protocols, and this may explain the relatively larger effect sizes in this geographical location, since high integrity is associated with better treatment outcomes and especially for anxiety treatment outcomes (Power et al., 2022). Despite these differences, all continents demonstrated positive change for all outcomes (*d* = 0.59–1.10) supporting the *universality hypothesis* (i.e., that psychotherapy is assumed to work across cultures; Flückiger et al., 2018).

Consistent with several prior meta-analytic reviews (e.g., Cuijpers et al., 2014b; Driessen et al., 2010; Furukawa et al., 2017), symptom severity did not predict effectiveness of treatment for depression. For anxiety outcomes, services categorized as treating mild conditions consistently had larger effect sizes. It is possible that classifying by type of service provided an imprecise proxy for sample severity and therefore future research should explore severity as a continuous variable in routine settings.

### Limitations

The most notable critique of this review is that it is based exclusively on evidence from observational studies. We are unable to rule out alternative explanations for observed effect sizes [placebo effects, spontaneous remission (Posternak & Miller, 2001; Whiteford et al., 2012)] and subsequently the observed effect sizes in this review cannot be directly compared to efficacy trials. Nevertheless, pooled effect sizes from observational studies serve as a valuable data source for benchmarking of routine care and quality improvement initiatives (e.g., Clark et al., 2018; Delgadillo et al., 2014; Gyani et al., 2013).

A key design limitation concerns statistical dependency. Efforts to avoid statistical dependency included: (i) taking one sample measure per domain, (ii) aggregating multiple unique study samples within a single domain, and (iii) extracting one measurement tool per study, per construct (i.e., preference system). These approaches have well-documented limitations (Borenstein et al., 2021; Hoyt & Del Re, 2018; Van den Noortgate et al., 2013). A preferable approach would have been to model dependency using a multi-level analysis (Van den Noortgate et al., 2013, 2015) or through robust variance estimation and should be considered for future replications. Use of robust-variance estimation would avoid the need to assign outcomes to a restrictive number of outcome domains. This would also circumvent the need to adopt a highly heterogeneous “other” outcome domain, which for the current review included both diagnosis specific and global distress-based measures.

An additional limitation concerns the inherent limitations of the risk-of-bias assessment tool which was selected for this study a priori. It could be argued that this tool primarily indexes manuscript reporting detail and not necessarily risk of bias. Future reviews of effectiveness could consider assessing methodological rigour using other available rating tools (e.g., see Munder & Barth, 2018).

Due to resource constraints and the large number of included studies, the systematic search, data extraction and risk-of-bias ratings were not performed completely in duplicate. For the subsample of full texts screened by two coders there was a strong, but imperfect, agreement/reliability (80%, $\kappa$ = 0.65). Similarly, not extracting data or assessing RoB in duplicate is problematic due to risk of imprecise estimates of treatment effect and RoB (Armijo-Olivo et al., 2014). An additional limitation surrounds coding decisions for moderator variables. Therapy modality was coded from manuscript self-definition. The degree to which treatments truly resembled treatment code (or treatment intended) is not clear. It was also apparent during extraction that very few practice-based studies report fidelity/adherence checks. As this becomes more routinely reported opportunities for modelling differences based on adherence/competence/integrity will become available. The use of categorical moderator levels to differentiate samples at the study level may also have provided imprecise proxies for moderator levels. For example, patient severity would preferably be modelled through meta-regression at the patient level to account for the heterogeneity within samples as it has been shown that university counselling center samples have numerous highly distressed individuals (Xiao et al., 2017). Future studies investigating these moderator variables at the patient level (e.g., through individual participant data meta-analysis) would help to shed light on this.

The search strategy is unlikely to have identified every available study. Search terms were based on prior reviews and omitted several terms that were found to produce an unmanageable number of records (e.g., “effectiveness”, “evaluation”). Despite this, the current reviews gives an adequate range and depth of effectiveness research with which to make tentative interpretations regarding the field of psychotherapy effectiveness research. A final caveat is the decision to focus exclusively on self-report measures of effectiveness. Meta-analytic evidence has demonstrated significant differences between self-report and clinician rated measures of clinical improvement (Cuijpers et al., 2010). Future research is therefore needed to see if the pooled effect-sizes from this study are consistent with clinician-rated measures of effectiveness in routine settings.

## Conclusions

This review provides support for the effectiveness of psychological therapy as delivered in routine settings across a range of outcomes. Overall, the effects of psychotherapy appear to generalize well to diverse clinical settings, contexts, and populations. Nevertheless, it is evident that treatment effects vary considerably across services, and this review provides performance benchmarks to support routine service evaluation and practice development initiatives.

## Supplementary Information

Below is the link to the electronic supplementary material.

Supplementary file1 (DOCX 324 kb)

## Data Availability

Data for the systematic review and related code will all be made publicly available through the lead author’s GitHub account following publication. This review followed the PRISMA and MAP-24 reporting guidelines for conducting systematic review/meta-analysis.

## Footnotes

1 Protocol available at: https://www.crd.york.ac.uk/prospero/display_record.php?RecordID=175235.

2 The authors recognise that use of the term *effectiveness* may be somewhat misleading. The pre–post (uncontrolled) methodology which forms the body of evidence in this review is unable to disentangle treatments effects from other potential causes of change (e.g., regression to the mean, placebo). Observed change in symptoms may therefore not exclusively represent treatment effectiveness. We have opted to retain use of this term within the current review because it has consistently and frequently been used as such in the extant literature (e.g., Lambert, 2013; Nordmo et al., 2020).

3 University clinics refers to university managed clinics treating communities beyond the student population. University counselling centres that are more specifically targeted at the student population are included within the mild category.
