# Supplementary material for: The Effectiveness of Psychological Interventions Delivered in Routine Practice: Systematic Review and Meta-analysis
Source: Adm Policy Ment Health. 2022 Oct 6;50(1):43–57. doi: 10.1007/s10488-022-01225-y (PMC9832112; doi:10.1007/s10488-022-01225-y)
Supplement: Supplementary file 1 — Supplementary file1 (DOCX 324 kb) [file 10488_2022_1225_MOESM1_ESM.docx]

The Effectiveness of Psychological Interventions Delivered in Routine Practice:

Systematic Review and Meta-Analysis

**Supplementary Material**

Table of Contents

[Figure 1: 3](#_Toc91190205)

[Forest plot of pre-post psychological therapy effect sizes for depression outcomes. 3](#_Toc91190206)

[Figure 2: 4](#_Toc91190207)

[Forest plot of pre-post psychological therapy effect sizes for anxiety outcomes. 4](#_Toc91190208)

[Figure 3: 5](#_Toc91190209)

[Forest plot of pre-post psychological therapy effect sizes for miscellaneous outcomes. 5](#_Toc91190210)

[Table 1: 6](#_Toc91190211)

[Inclusion and exclusion criteria used in the current review, shown using the PICOS framework (population, intervention, comparator, outcome, setting). 6](#_Toc91190212)

[Table 2: 7](#_Toc91190213)

[List of search terms and limiters for systematic database search 7](#_Toc91190214)

[Preference system for outcome measures 8](#_Toc91190215)

[Additional Methodology Information 9](#_Toc91190216)

[Effect-Size Calculation 11](#_Toc91190217)

[Table 3: 11](#_Toc91190218)

[Hierarchical procedure for effect-size calculation. 11](#_Toc91190219)

[Table 4 12](#_Toc91190220)

[Study characteristics. 12](#_Toc91190221)

[Table 5: 36](#_Toc91190222)

[Effect size data for the studies included in the meta-analysis. 36](#_Toc91190223)

[Table 6 55](#_Toc91190224)

[*Bibliography* 56](#_Toc91190225)

# Figure 1:

## Forest plot of pre-post psychological therapy effect sizes for depression outcomes.

******

*Note.* Square boxes depict individual study Cohen’s d effect sizes, error bars display 95 percent confidence intervals, and the diamond represents the pooled estimate effect.

# Figure 2:

## Forest plot of pre-post psychological therapy effect sizes for anxiety outcomes.

****** *Note.* Square boxes depict individual study Cohen’s d effect sizes, error bars display 95 percent confidence intervals, and the diamond represents the pooled estimate effect.

# Figure 3:

## Forest plot of pre-post psychological therapy effect sizes for miscellaneous outcomes.

****** *Note.* Square boxes depict individual study Cohen’s d effect sizes, error bars display 95 percent confidence intervals, and the diamond represents the pooled estimate effect.

| Table 1:Inclusion and exclusion criteria used in the current review, shown using the PICOS framework (population, intervention, comparator, outcome, setting). | | |
| --- | --- | --- |
| **Criteria** | **Inclusion** | **Exclusion** |
| Population | Sample exclusively aged 16 and above (lower end of sample age range is at least 16). | Adolescent/child samples with a lower age limit below 16. |
| Intervention | Psychological intervention which includes individual face-to-face psychological therapy (i.e. at least one session). | Samples which indicate that any proportion of patients did not recieve at least one session of individual psychological therapy. |
| Comparator | Studies with pre and post intervention time points. Post intervention defined here as up to six months following treatment. | 1. Studies which do not report both pre and post intervention time points. 2. Studies for which the post intervention time point is beyond six months following treatment termination. 3. (iii) Treatment randomisation procedures. |
| Outcome | Psychological treatment effectiveness using a validated self-report measurement tool. | Service/settings which do not use a self-report measure of psychological effectiveness. Clinician reported measures were not included in this review. |
| Setting | Services for which a patient could expect to access psychological therapy (i.e. routine services). | Service/settings that strongly do not appear naturalistic or reflect routine practice. |
| Design | 1. Pre-post treatment designs. 2. Studies which do not use a control condition. | 1. Studies which include a control group. 2. Studies with N = <6. 3. Results not available/published in English. |

***Systematic Review Search Terms***

| Table 2:List of search terms and limiters for systematic database search | | |
| --- | --- | --- |
| Effectiveness Relevance | Psychological Relevance | Limiters |
| ‘Practice based evidence’ | Psycho* OR Therap [PsycInfo] | English Language |
| ‘Routine practice’ | Psycho* [CINAHL and MEDLINE] | Adult Sample |
| Benchmarking |  |  |
| Transportability |  |  |
| Transferability |  |  |
| Clinical* representat |  |  |
| ’External valid* N0 findings |  |  |
| Applicab* N0 findings |  |  |
| Applicab* N0 intervention* |  |  |
| ’Empiric* support*’ N0 treatment* |  |  |
| ’Empiric* support*’ N0 intervention* |  |  |
| ’Clinical* Effective*’ |  |  |
| Dissem* N0 treatment* |  |  |
| Dissem* N0 intervention* |  |  |
| ‘Clinical Practice’ N0 intervention* |  |  |
| ‘Clinical Practice’ N0 treatment* |  |  |
| ’Service deliv*’ N0 intervention* |  |  |
| ’Service deliv*’ N0 treatment* |  |  |
| ’Clinical* effective*’ N2 evaluat* |  |  |
| ’Service deliv*’ N0 evaluat* |  |  |
| Transporting |  |  |
| ‘Managed care setting’ |  |  |
| Uncontrolled |  |  |
| ‘Community clinic’ |  |  |
| ‘Community mental health centre’ |  |  |
| ‘Clinic setting’ |  |  |
| ‘Service setting’ |  |  |

# Preference system for outcome measures

Because of the heterogeneity of outcome measures which could fit within the ‘general’ category the following hierarchy was used:

- 1. global measures of psychological distress (e.g., CORE-OM, SCL-90).
  2. mono-symptomatic measures (e.g., Y-BOCS, EDE-Q),
  3. peripheral outcomes scales indicating symptom amelioration (e.g., functioning, quality of life).

If a study used more than one measure at the same stage in the hierarchy then we used the measure that had been most frequently employed in studies reviewed prior. Below is the final table of outcome measures used in the general category.


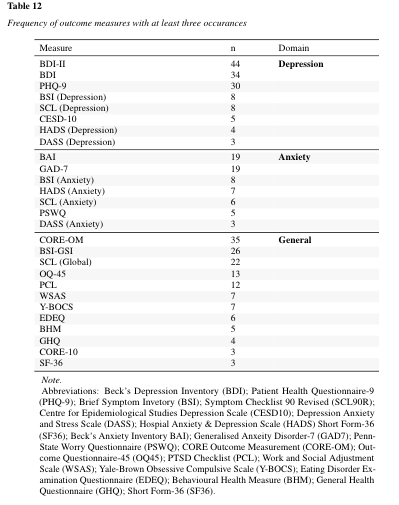


# Additional Methodology Information

***Extraction and Coding Information***

There was high variability of demographic reporting for each study (e.g. gender, age, ethnicity etc.). For demographic information, the (i) mean age of each sample was extracted, and then (when reported) the number and percentage of: (ii) female, (iii), minority ethnic group, (iv) full-time employed, (v) and married patients. Each of these variables were summarised by averaging across mean averages for studies which reported this information.

***Methodological Information******:***

For methodological information the type of completion analysis used was extracted. Samples were coded as either true ITT (everyone had an equal chance of inclusion), modified ITT, or completers. The stage of the hour-glass model was also recorded for each effectiveness study. Samples were rated as either stage-1 (pilot and preliminary effectiveness studies) or stage-3 (evaluation/benchmarking studies studies). The region (country and continent) was recorded; studies from the UK were separated from mainland Europe, due to the high volume of effectiveness research originating in the UK.

***Service Information******:***

The type of service and associated sector were extracted for each study. As there were a large number of different sectors represented, a grouping system clustered similar sectors together. Services from primary care, health settings, counselling, and voluntary services were collated into a ‘primary’ sector category. Services delivering interventions for more specialist, complex or enduring presentations were grouped into a ‘secondary’ category. This included specialist/tertiary therapy services/clinics, community mental health teams/centers, and intensive out-patient services. University based services (either training clinics or counseling centers) were assigned to a ‘University clinics’ category. Finally, inpatient, day hospital and partial hospital services were grouped into a ‘inpatient’ category. Whether or not study interventionists consisted of clinicians in training was also recorded as a separate variable. We defined clinicians in training as staff training towards a professional psychology training course (i.e. clinical psychology interns/students, training psychiatrists or assistant psychologists). Staff who were not psychologists or qualified therapists, but who had a core profession (e.g. nurses, social workers) were not recognised as unqualified interventionists.

***Treatment Information:***

The treatment delivered was recorded for each study. Treatments were then assigned to a broad meta-therapy category, including: (i) cognitive and/or behavioural, (ii) dynamic/interpersonal, (iii) person-centered counselling (or counselling without a specified orientation), or (iv) other/non-specified. The average number of sessions was also recorded. For studies that reported the mean number of sessions then this was the metric extracted. For studies that alternatively used a time metric (days/weeks/months/years) then a uniform metric was applied (i.e. conversion to days). There was subsequently two possible dosage metrics, sessions of treatment and treatment days. If studies reported sample dosage, but with an alternative measure of central tendency (i.e. median) then this was converted to mean average.

# Effect-Size Calculation

As many manuscripts did not report all the required information for calculating this variant of Cohen’s d, a hierarchical stepped approach was used to handle the missing information (see Table 3). For studies which reported all the required information (N, M^1^, M^2^, SD^1^, r) then d was calculated without additional consideration. For studies which (commonly) did not report r then we e-mailed corresponding authors (two-week response time) to request this information. When unsuccessful r was imputed using an empirically supported estimate [r = .60, [36](http://127.0.0.1:22959/rmd_output/3/#ref-Balk2012)]. For studies which did not provide the more fundamental figures (M^1^, M^2^ or SD^1^) but reported a paired samples Cohen’s d (any variant) then this effect-size was extracted. For studies which did not report fundamental figures and did not report a Cohen’s d, then e-mail requests were sent to corresponding authors. If this was unsuccessful then we applied conversion formulas in situations when studies reported alternative quantitative metrics to generate means and standard deviations. When all steps were unsuccessful/not applicable the study in question were removed.

| Table 3:Hierarchical procedure for effect-size calculation. | | |
| --- | --- | --- |
| **Steps** | **Scenario** | **Response** |
| Step 1 | Manuscript reports all required information (N, M1, M2, SD1) for preferred d. | Calculate preferred d. |
| Step 2 | Manuscript reports all information apart from Pearson’s r. | E-mail corresponding authors to request r. |
| Step 3 | Manuscript does not report the mean or standard deviation but reports paired samples d. | Use the reported d within the manuscript. |
| Step 4 | Manuscript does not report mean, standard deviation, or paired samples d however reports alternative metrics (e.g. median, range, standard error, ANOVA, regression | Estimate the mean and standard deviation by converting available metrics. |
| Step 5 | All above steps attempted without success | Study is not included in meta-analysis but is retained for narrative synthesis. |

# Table 4

## Study characteristics.

| **Citation** | **Country** | **Service** | **Sector** | **N** | **Primary Problem** | **Therapy** | **Analysis** | **Bias Score** | |
| --- | --- | --- | --- | --- | --- | --- | --- | --- | --- |
| Aazh & Moore, 2018 | England | Tinnitus and Hyperacusis Therapy Specialist Clinic | Primary | 68 | Health or Medically Unexplained | CBT | ITT | 7 | |
| Abbass, 2002 | Canada | Private Psychotherapy Practice | Private | 89 | Various or Not Specific | Dynamic | Completers | 6 | |
| Abbass, 2006 | Canada | Mood Disorders Program at Dalhousie University | Uni. Clinics | 10 | Depression | Dynamic | Assume ITT | 7 | |
| Abbass et al., 2008 | Canada | Tertiary Psychotherapy Service | Secondary | 30 | Various or Not Specific | Dynamic | Assume ITT | 7 | |
| Abbass et al., 2009 | Canada | Emergency Department | Primary | 50 | Health or Medically Unexplained | Dynamic | Modified ITT | 7 | |
| Abbass, 2013 | Canada | Acute Psychiatric Inpatient Service | Residential | 23 | Various or Not Specific | Dynamic | Completers | 4 | |
| Abramowitz et al., 2003 | USA | Two Speciality Clinics for Anxiety/OCD | Secondary | 132 | OCD | CBT | ITT | 8 | |
| Adamson et al., 2020 | England | National Persistent Physical Symptoms Research and Treatment Unit | Primary | 995 | Health or Medically Unexplained | CBT | Completers | 7 | |
| Andrews et al., 2011 | England | Practice Research Network for Primary Care Counselling | Primary | 124 | Various or Not Specific | Counselling | Modified ITT | 4 | |
| Andrews et al., 2013 | England | Practice Research Networy for Human Givens | Primary | 3885 | Various or Not Specific | Counselling | Modified ITT | 3 | |
| Archer et al., 2000 | England | Large Voluntary Sector Counselling Service (NHS) | Primary | 90 | Various or Not Specific | Counselling | Assume ITT | 3 | |
| Armstrong, 2010 | Scotland | Voluntary Sector Counselling Service | Primary | 118 | Various or Not Specific | Counselling | Completers | 5 | |
| Asay et al., 2002 | USA | Private Practice | Private | 29 | Various or Not Specific | Dynamic | Modified ITT | 5 | |
| Ashworth et al., 2015 | England | Outpatient Neuro-Rehabilitation Service | Primary | 12 | Health or Medically Unexplained | CBT | Completers | 6 | |
| Askey-Jones et al., 2013 | England | Multiple Sclerosis Clinic | Primary | 22 | Health or Medically Unexplained | CBT | Completers | 7 | |
| Asnaani et al., 2020 | USA | Specialist Anxiety Clinic | Secondary | 489 | Anxiety | CBT | ITT | 7 | |
| Back et al., 2020 | Sweden | Specialist Eating Disorder Services | Secondary | 16 | Various or Not Specific | Dynamic | ITT | 6 | |
| Back et al., 2017 | Sweden | Seven outpatient psychiatric services specialising in ED treatment in Sweden. " | Secondary | 31 | Eating Disorders | Dynamic | ITT | 4 | |
| Bados et al., 2007 | Spain | University Behavioural Therapy Unit | Uni. Clinics | 96 | Anxiety | CBT | Completers | 6 | |
| Baldwin et al., 2009 | USA | University Counselling Centre | University Counselling | 4676 | Various or Not Specific | Other | Modified ITT | 5 | |
| Bales et al., 2012 | Netherlands | Day Hospital | Residential | 45 | Personality Disorders | Dynamic | ITT | 8 | |
| Ballesteros & Labrador, 2014 | Spain | University Psychology Clinic | Uni. Clinics | 50 | Anxiety | CBT | Completers | 6 | |
| Banham & Schweitzer, 2016 | Australia | University Psychology Clinic | Uni. Clinics | 611 | Various or Not Specific | Other | Check | NA | |
| Barkham et al., 2001 | England | Six Secondary Care Services | Secondary | 224 | Various or Not Specific | Other | Completers | 4 | |
| Barkham et al., 2012 | UK | Primary Care Services | Primary | 9761 | Various or Not Specific | Other | ITT | 3 | |
| Barnitcot & Crawford, 2018 | UK | Inpatient Personality Disorder Services | Residential | 58 | Personality Disorders | CBT | Modified ITT | 5 | |
| Barnitcot & Crawford, 2018 | UK | Inpatient Personality Disorder Services | Residential | 32 | Personality Disorders | Dynamic | Modified ITT | 5 | |
| Baronian & Leggett, 2020 | England | Pain Management Service | Primary | 53 | Health or Medically Unexplained | Other | Completers | 7 | |
| Beail et al., 2005 | England | Community Learning Disabilities Service | Secondary | 20 | Various or Not Specific | Dynamic | Completers | 5 | |
| Beard et al., 2016 | USA | Partial Hospital Setting | Residential | 956 | Various or Not Specific | CBT | ITT | 6 | |
| Ben-Porath et al., 2004 | USA | CMHC | Secondary | 23 | Personality Disorders | CBT | Completers | 6 | |
| Birtchnell et al., 2005 | England | Psychotherapy Department | Secondary | 49 | Various or Not Specific | Other | Completers | 4 | |
| Bitran et al., 2008 | USA | University Based Clinic | Uni. Clinics | 40 | Anxiety | CBT | Completers | 5 | |
| Bjorgvinsson et al., 2014 | USA | Partial Hospital Program | Secondary | 951 | Various or Not Specific | CBT | ITT | 6 | |
| Blainey et al., 2017 | England | Tertiary Psychotherapy Service for ASC | Secondary | 81 | Various or Not Specific | CBT | Modified ITT | 7 | |
| Boettcher et al., 2019 | Germany | University Outpatient Clinic | Uni. Clinics | 105 | Various or Not Specific | CBT | ITT | 8 | |
| Bradshaw et al., 2009 | USA | CMHC | Secondary | 78 | Various or Not Specific | Dynamic | Completers | 6 | |
| Brand, 2020 | Republic of Ireland | Primary Care Counselling Services | Primary | 2781 | Various or Not Specific | Counselling | ITT | 7 | |
| Briggie et al., 2016 | USA | University Based Community Mental Health Clinic | Uni. Clinics | 390 | Various or Not Specific | Dynamic | Completers | 6 | |
| Brunnbauer et al., 2016 | Australia | University Training Clinic | Uni. Clinics | 82 | Various or Not Specific | CBT | ITT | 6 | |
| Buckley et al., 2006 | England | Community Based Clinical Psychology Department | Secondary | 60 | Various or Not Specific | Other | Completers | 5 | |
| Buckley et al., 2006 | England | Community Based Clinical Psychology Department | Secondary | 60 | Various or Not Specific | Other | Completers | 5 | |
| Budge et al., 2013 | USA | University Counselling Centre | University Counselling | NA | Various or Not Specific | Counselling | Modified ITT | 6 | |
| Budge et al., 2013 | USA | University Counselling Centre | University Counselling | NA | Various or Not Specific | Counselling | Modified ITT | 6 | |
| Budge et al., 2013 | USA | University Counselling Centre | University Counselling | NA | Various or Not Specific | Counselling | Modified ITT | 6 | |
| Budge et al., 2013 | USA | University Counselling Centre | University Counselling | 2574 | Various or Not Specific | Counselling | Modified ITT | 6 | |
| Buckley et al., 2016 | England | Practice Research Network for Voluntary Sector PTSD Counselling | Primary | 504 | PTSD | Counselling | ITT | 4 | |
| Burlingame et al., 2016 | USA | University Counselling Centre | University Counselling | 1557 | Various or Not Specific | Other | Modified ITT | 4 | |
| Burlingame et al., 2016 | USA | University Counselling Centre | University Counselling | 11764 | Various or Not Specific | Other | Modified ITT | 4 | |
| Butler et al., 2020 | USA | University Outpatient Clinic | Uni. Clinics | 93 | Anxiety | CBT | Assume ITT | 6 | |
| Byrne et al., 2011 | Australisa | Eating Disorders Service | Secondary | 125 | Eating Disorders | CBT | ITT | 8 | |
| Cahill et al., 2003 | England | University Based Service for Community Mental Health | Uni. Clinics | 58 | Depression | CBT | ITT | 8 | |
| Callahan et al., 2005 | USA | University Training Clinic | Uni. Clinics | 61 | Various or Not Specific | Other | Completers | 5 | |
| Carney et al., 2011 | Canada | Specialist CBT Clinic for Addiction | Secondary | 24 | Depression | CBT | Assume ITT | 4 | |
| Carr et al., 2017 | USA | University Training Clinic | Uni. Clinics | 132 | Various or Not Specific | Other | Modified ITT | 3 | |
| Carter, 2005 | Wales | CMHT | Secondary | 42 | Various or Not Specific | Other | Check | 3 | |
| Chase et al., 2015 | USA | Residential OR Intensive Outpatient Program for OCD | Mixed | 134 | OCD | CBT | Assume ITT | 4 | |
| Christiansen et al., 2015 | Germany | University Behavioural Therapy Unit | Uni. Clinics | 87 | Health or Medically Unexplained | CBT | ITT | 6 | |
| Clapp et al., 2013 | USA | Inpatient Facility | Residential | 1084 | Depression | Other | Completers | 4 | |
| Connell et al., 2008 | England | Muliple UCCs | University Counselling | 846 | Various or Not Specific | Other | ITT | 5 | |
| Cooper et al., 2017 | Canada | Academic family medicine teams (primary care) | Primary | 100 | Health or Medically Unexplained | Dynamic | Assume ITT | 6 | |
| Daig et al., 2009 | Germany | Inpatient Psychosomatic Service | Residential | 1442 | Health or Medically Unexplained | Dynamic | Modified ITT | 7 | |
| Davis et al., 2008 | England | Primary Care Counselling Service | Primary | 58 | Various or Not Specific | Counselling | Completers | 4 | |
| Davis et al., 2010 | USA | University Based Clinic | Uni. Clinics | 150 | Various or Not Specific | CBT | Completers | NA | |
| de Jongh et al., 2011 | UK | Private Insurance Company | Private | 125 | Anxiety | CBT | ITT | 7 | |
| de Jongh et al., 2011 | UK | Private Insurance Company | Private | 59 | Anxiety | CBT | ITT | 7 | |
| Delgadillo et al., 2020 | UK | Primary Care Service (IAPT) | Primary | 1435 | Depression | Other | Modified ITT | 4 | |
| Dennhag & Armelius, 2012 | Sweden | University Training Clinic | Uni. Clinics | 187 | Various or Not Specific | Other | Completers | 6 | |
| Dickson & Gullo, 2015 | England | Student Health Centre | Check with team | 48 | Various or Not Specific | CBT | Check | 4 | |
| Doorn et al., 2014 | England | Secondary Care Psychological Therapy Services | Secondary | 31 | Various or Not Specific | Dynamic | Assume ITT | 6 | |
| Douglas et al., 2016 | England | Tertiary Psychotherapy Service | Secondary | 28 | Various or Not Specific | Dynamic | Assume ITT | 5 | |
| Ehlers et al., 2013 | England | Specialist Anxiety & Trauma Clinic | Secondary | 330 | PTSD | CBT | ITT | 8 | |
| Ellison et al., 2013 | USA | University Based Service for Community Mental Health | Uni. Clinics | 60 | Various or Not Specific | Other | Modified ITT | NA | |
| Erekson et al., 2013 | USA | University Counselling Centre | University Counselling | 3092 | Various or Not Specific | Counselling | Modified ITT | NA | |
| Erekson et al., 2013 | USA | University Counselling Centre | University Counselling | 3092 | Various or Not Specific | Counselling | Modified ITT | NA | |
| Puig et al., 2012 | Spain | University Assistential Clinic | Uni. Clinics | 44 | Depression | CBT | Completers | 6 | |
| Evans et al., 2017 | England | Secondary Care Psychological Therapy Team | Secondary | 925 | Various or Not Specific | Other | Completers | 4 | |
| Falkenstein et al., 2019 | USA | Inpatient Program | Residential | 306 | OCD | CBT | Check | NA | |
| Falkenstrom, 2010 | Sweden | Community Based Counselling Clinic for Young Adults | Primary | 101 | Various or Not Specific | Dynamic | Completers | 5 | |
| Fizke et al., 2002 | Germany | Psychosomatic and psychotherapy inpatient units | Residential | 234 | Depression | Dynamic | Completers | 7 | |
| Fizke et al., 2010 | Germany | Psychosomatic and psychotherapy inpatient units | Residential | 506 | Depression | Dynamic | Completers | 7 | |
| Flo et al., 2014 | England | CFS Unit | Primary | 140 | Health or Medically Unexplained | CBT | Completers | 4 | |
| Flygare et al., 2020 | Sweden | Specialist OCD Clinic | Secondary | 19 | OCD | CBT | ITT | 8 | |
| Forand et al., 2011 | USA | Cognitive Therapy Clinic | Uni. Clinics | 249 | Various or Not Specific | CBT | Modified ITT | 6 | |
| Fortune et al., 2005 | England | Clinical Psychology Service with Two Strand - Primary & Secondary | Mixed | 52 | Various or Not Specific | CBT | Modified ITT | 4 | |
| Fowler et al., 2017 | USA | Specialised Psychiatric Hospital | Residential | 994 | Various or Not Specific | Other | Modified ITT | 7 | |
| Fowler et al., 2018 | USA | Specialised Psychiatric Hospital | Residential | 245 | Personality Disorders | Dynamic | Modified ITT | 6 | |
| Fox et al., 2015 | England | Low Secure Mental Health Hospital | Residential | 18 | Personality Disorders | CBT | Completers | 7 | |
| Frueh et al., 2009 | USA | CMHC | Secondary | 20 | Various or Not Specific | CBT | Completers | 6 | |
| Galili-Weinstock et al., 2018 | Israel | University Outpatient Clinic | Uni. Clinics | 112 | Various or Not Specific | Dynamic | Completers | 3 | |
| Gamble et al., 2013 | USA | Substance Misuse Clinic | Secondary | 14 | Various or Not Specific | Dynamic | ITT | 5 | |
| Ghafoori et al., 2019 | USA | Community Based Mental Health Agency | Secondary | 88 | PTSD | CBT | Completers | 5 | |
| Ghafoori et al., 2019 | USA | Community Based Mental Health Agency | Secondary | 95 | PTSD | Other | Completers | 5 | |
| Ghafoori et al., 2019 | USA | Community Based Mental Health Agency | Secondary | 206 | PTSD | Counselling | Completers | 5 | |
| Ghafoori et al., 2019 | USA | Community Based Mental Health Agency | Secondary | 137 | PTSD | Other | Completers | 5 | |
| Ghilardia et al., 2018 | Italy | University Counselling Centre | University Counselling | 80 | Various or Not Specific | Counselling | Completers | 6 | |
| Gibbard & Hanley, 2008 | England | Counselling Service | Primary | 1098 | Various or Not Specific | Counselling | Completers | 7 | |
| Gibbons et al., 2010 | USA | Outpatient Clinic for Cognitive Therapy | Secondary | 217 | Depression | CBT | ITT | 5 | |
| Gilbert et al., 2005 | England | Trust of Primary Care Services | Primary | 2205 | Various or Not Specific | CBT | Completers | 7 | |
| Gillespie et al., 2002 | Northern Ireland | Community Trauma & Recovery Team | Secondary | 91 | PTSD | CBT | ITT | 8 | |
| Gimeno-Peon et al., 2019 | Spain | Private Practice | Private | 42 | Various or Not Specific | Other | Modified ITT | 5 | |
| Goldberg et al., 2016 | USA | University Counselling Centre | University Counselling | 6591 | Various or Not Specific | Other | Modified ITT | 6 | |
| Gordon, 2001 | USA | Private Practice | Private | 55 | Various or Not Specific | Dynamic | Modified ITT | 6 | |
| Graca et al., 2014 | USA | PTSD Residential Program from VA | Residential | 51 | PTSD | CBT | Assume ITT | 5 | |
| Graca et al., 2014 | USA | PTSD Residential Program from VA | Residential | 51 | PTSD | CBT | Assume ITT | 5 | |
| Greasley & Small, 2005 | England | Primary Care Counselling Service | Primary | 89 | Various or Not Specific | Counselling | Completers | 6 | |
| Gropalis et al., 2012 | Germany | University Outpatient Clinic | Uni. Clinics | 224 | Anxiety | CBT | ITT | 6 | |
| Gropalis et al., 2012 | Germany | University Outpatient Clinic | Uni. Clinics | 65 | Anxiety | CBT | ITT | 6 | |
| Gropalis et al., 2012 | Germany | University Outpatient Clinic | Uni. Clinics | 94 | Check | CBT | ITT | 6 | |
| Guthrie et al., 2004 | England | Check | Primary | 34 | Various or Not Specific | Dynamic | Completers | 3 | |
| Haase et al., 2008 | Germany | Hospital Based Treatment | Residential | 158 | Various or Not Specific | Dynamic | Assume ITT | 4 | |
| Hahlweg et al., 2001 | Germany | Multiple Outpatient Clinics | Uni. Clinics | 416 | Anxiety | CBT | Completers | 6 | |
| Halje et al., 2015 | Sweden | Young Adults Centre | Secondary | 607 | Various or Not Specific | CBT | ITT | 8 | |
| Harnett et al., 2010 | Australia | University Psychology Clinics | Uni. Clinics | 125 | Various or Not Specific | Other | Modified ITT | 5 | |
| Harte & Hawkins, 2016 | USA | Clinical Psychology Training Clinic | Uni. Clinics | 191 | Depression | CBT | Assume ITT | 6 | |
| Harte & Hawkins, 2016 | USA | Clinical Psychology Training Clinic | Uni. Clinics | 114 | Depression | CBT | Assume ITT | 6 | |
| Harte & Hawkins, 2016 | USA | Clinical Psychology Training Clinic | Uni. Clinics | NA | Anxiety | CBT | Assume ITT | 6 | |
| Harte & Hawkins, 2016 | USA | Clinical Psychology Training Clinic | Uni. Clinics | NA | Anxiety | CBT | Assume ITT | 6 | |
| Harte & Hawkins, 2016 | USA | Clinical Psychology Training Clinic | Uni. Clinics | NA | Anxiety | CBT | Assume ITT | 6 | |
| Harte & Hawkins, 2016 | USA | Clinical Psychology Training Clinic | Uni. Clinics | NA | Anxiety | CBT | Assume ITT | 6 | |
| Harte & Hawkins, 2016 | USA | Clinical Psychology Training Clinic | Uni. Clinics | NA | Anxiety | CBT | Assume ITT | 6 | |
| Harte & Hawkins, 2016 | USA | Clinical Psychology Training Clinic | Uni. Clinics | NA | Anxiety | CBT | Assume ITT | 6 | |
| Harte & Hawkins, 2016 | USA | Clinical Psychology Training Clinic | Uni. Clinics | NA | Anxiety | CBT | Assume ITT | 6 | |
| Harte & Hawkins, 2016 | USA | Clinical Psychology Training Clinic | Uni. Clinics | NA | Anxiety | CBT | Assume ITT | 6 | |
| Harte & Hawkins, 2016 | USA | Clinical Psychology Training Clinic | Uni. Clinics | NA | Anxiety | CBT | Assume ITT | 6 | |
| Harte & Hawkins, 2016 | USA | Clinical Psychology Training Clinic | Uni. Clinics | NA | Anxiety | CBT | Assume ITT | 6 | |
| Haugen et al., 2017 | USA | World Trade Centre Responder Clinic | Secondary | 32 | PTSD | Other | ITT | 8 | |
| Heins et al., 2011 | Netherlands | Tertiary Care CFS | Secondary | 80 | Health or Medically Unexplained | CBT | ITT | 8 | |
| Heins et al., 2011 | Netherlands | Tertiary Care CFS | Secondary | 136 | Health or Medically Unexplained | CBT | ITT | 8 | |
| Hill et al., 2015 | USA | University Training Clinic | Uni. Clinics | 121 | Various or Not Specific | Dynamic | Modified ITT | 3 | |
| Hilsenroth et al., 2003 | USA | Community Outpatient Clinic | Secondary | 21 | Depression | Dynamic | Completers | 7 | |
| Hiltunen et al., 2013 | Sweden | University Training Clinic | Uni. Clinics | 35 | Various or Not Specific | CBT | Completers | 3 | |
| Hiney-Saunders et al., 2019 | England | Specialist Eating Disorders Unit | Residential | 44 | Eating Disorders | CBT | ITT | 4 | |
| Hirsch et al., 2000 | England | Psychology Service | Secondary | 42 | Anxiety | CBT | Completers | 4 | |
| Hirsch et al., 2000 | England | Psychology Service | Secondary | 52 | Depression | CBT | Completers | 4 | |
| Hitt et al., 2018 | Wales | Occupational Health Service | EAP/OH | 76 | Various or Not Specific | CBT | Completers | 6 | |
| Holmqvist et al., 2013 | Sweden | Primary Care Centres | Primary | 733 | Various or Not Specific | Other | Modified ITT | 6 | |
| Houghton et al., 2010 | England | Psychotherapy Service | Secondary | 37 | OCD | CBT | ITT | 6 | |
| Jakupcak et al., 2010 | USA | Specialty Postdeployment Primary Care Clinic | Primary | 6 | Depression | CBT | Completers | 6 | |
| Jankowski et al., 2019 | USA | Psychodynamic community mental health training clinic | Uni. Clinics | 118 | Various or Not Specific | Dynamic | Modified ITT | NA | |
| Jenkins et al., 2019 | England | Specialist Eating Disorders Service | Secondary | 63 | Eating Disorders | CBT | ITT | 8 | |
| Jepsen et al., 2009 | Norway | Inpatient CSA Program | Residential | 34 | PTSD | Other | Assume ITT | 7 | |
| Jepsen et al., 2014 | Norway | Inpatient CSA Program | Residential | 56 | PTSD | Other | Completers | 7 | |
| Johansson et al., 2014 | Canada | Tertiary Psychotherapy Service | Secondary | 412 | Various or Not Specific | Dynamic | Completers | 6 | |
| Jolley et al., 2015 | England | IAPT SMI | Primary | 221 | Psychosis | CBT | Completers | 6 | |
| Jolley et al., 2015 | England | IAPT SMI | Primary | 20 | Psychosis | CBT | Completers | 1 | |
| Jones et al., 2008 | England | ICU Counselling Service | Primary | 46 | Various or Not Specific | Counselling | Check | 3 | |
| Jones et al., 2012 | England | Eating Disorders Service | Secondary | 48 | Eating Disorders | CBT | Completers | 2 | |
| Jordan et al., 2019 | England | CBT Clinic within Gastro Service | Primary | 27 | Health or Medically Unexplained | CBT | Completers | 7 | |
| Kaplinski, 2014 | USA | University Training Clinic | Uni. Clinics | 213 | Various or Not Specific | Other | Modified ITT | 5 | |
| Karlin et al., 2019 | USA | Non Profit Health Care System | Secondary | 36 | Depression | CBT | ITT | 3 | |
| Kehle et al., 2008 | USA | Frontline Service Settings - Two Clinics | Secondary | 29 | Anxiety | CBT | ITT | 6 | |
| Kellett et al., 2013 | England | Specialist & Tertiary Psychotherapy Services | Residential | 17 | Personality Disorders | Other | Completers | 6 | |
| Kikuchi et al., 2019 | Japan | Inpatients & Outpatients | Mixed | 7 | Health or Medically Unexplained | CBT | Completers | 4 | |
| Knoop et al., 2007 | Netherlands | University Medical Centre | Primary | 96 | Health or Medically Unexplained | CBT | Completers | 6 | |
| Knott et al., 2015 | Wales | Eating Disorders Service | Secondary | 272 | Eating Disorders | CBT | ITT | 7 | |
| Kobori et al., 2014 | Japan | University Outpatient Clinic | Uni. Clinics | 8 | Eating Disorders | CBT | ITT | 6 | |
| Kobori, et al., 2014 | Japan | University Outpatient Clinic | Uni. Clinics | 14 | OCD | CBT | ITT | 6 | |
| Kobori et al., 2014 | Japan | University Outpatient Clinic | Uni. Clinics | 19 | Anxiety | CBT | ITT | 6 | |
| Kolly et al., 2015 | Switzerland | University Hospital | Secondary | 13 | Personality Disorders | Other | Assume ITT | 6 | |
| Kramer et al., 2013 | Switzerland | Diverse Outpatient Settings | Secondary | 13 | Various or Not Specific | Other | Completers | 3 | |
| Kvarsteinet al., 2014 | Norway | Specialist Treatment Uni | Residential | 64 | Personality Disorders | Dynamic | Assume ITT | 5 | |
| Lambert et al., 2001 | USA | Various | Mixed | NA | Various or Not Specific | Other | NA | 7 | |
| Levitt et al., 2007 | USA | Community Outpatient Clinic | Secondary | 59 | PTSD | CBT | ITT | 1 | |
| Liness et al., 2019 | England | IAPT Trainees | Primary | 1927 | Various or Not Specific | CBT | Completers | 8 | |
| Lopez et al., 2011 | USA | CMHC | Secondary | 40 | Depression | CBT | ITT | 4 | |
| Lopez & Basco, 2015 | USA | CMHCs from 39 Local Mental Health Authorities | Secondary | 83 | Depression | CBT | Assume ITT | 6 | |
| LoSavio et al., 2019 | USA | Mental Health Agencies | Secondary | 242 | PTSD | CBT | ITT | 3 | |
| Low et al., 2001 | England | High Secure Hospital | Residential | 10 | Personality Disorders | CBT | Completers | 4 | |
| Lu et al., 2009 | USA | Behavioural Health Organisation | Secondary | 14 | PTSD | CBT | Completers | 6 | |
| Lunnen et al., 2008 | USA | CMHCs | Secondary | 66 | Various or Not Specific | Other | Modified ITT | 5 | |
| Lutz et al., 2002 | USA | Managed Care Companies | Secondary | 75 | Various or Not Specific | Other | Check | 6 | |
| Lutz et al., 2016 | Germany | University Outpatient Clinic | Uni. Clinics | 574 | Depression | CBT | Modified ITT | 2 | |
| Marriott & Kellett, 2009 | England | Psychotherapy Service | Secondary | 27 | Various or Not Specific | Other | Completers | 5 | |
| Marriott & Kellett, 2009 | England | Psychotherapy Service | Secondary | 27 | Various or Not Specific | CBT | Completers | 5 | |
| Marriott & Kellett, 2009 | England | Psychotherapy Service | Secondary | 25 | Various or Not Specific | Counselling | Completers | 5 | |
| Marriott & Kellett, 2009 | England | Psychotherapy Service | Secondary | 38 | Various or Not Specific | Other | Completers | 5 | |
| Marriott & Kellett, 2009 | England | Psychotherapy Service | Secondary | 38 | Various or Not Specific | CBT | Completers | 5 | |
| Marriott & Kellett, 2009 | England | Psychotherapy Service | Secondary | 38 | Various or Not Specific | Counselling | Completers | 5 | |
| Mayy, 1984 | USA | CMHC | Secondary | 131 | Various or Not Specific | Other | Modified ITT | NA | |
| McAleavey et al., 2019 | USA | Practice Research Network for UCCs | University Counselling | 9895 | Various or Not Specific | Counselling | Modified ITT | 5 | |
| McBride et al., 2010 | Canada | University Outpatient Clinic | Uni. Clinics | 74 | Depression | Dynamic | Completers | 5 | |
| McDevitt-Petrovic et al., 2018 | Ireland (Northern) | IAPT | Primary | 211 | Various or Not Specific | CBT | Modified ITT | 5 | |
| McEvoy et al., 2014 | Australia | Community Based Specialist Mental Health Clinic | Secondary | 84 | Various or Not Specific | CBT | Completers | 7 | |
| McHugh et al., 2014 | Ireland | Primary Care Service | Primary | 45 | Various or Not Specific | CBT | Completers | 5 | |
| McHugh et al., 2016 | Ireland | Primary Care Service | Primary | NA | Various or Not Specific | CBT | Completers | 3 | |
| McKenzie & Marks, 2003 | England | Inpatient Behavioural Unit | Residential | NA | OCD | CBT | Completers | 6 | |
| McLeod et al., 2000 | England | EAP | EAP/OH | 235 | Various or Not Specific | Counselling | Completers | 2 | |
| McLeod et al., 2000 | England | Counselling Centre | Primary | 265 | Various or Not Specific | Counselling | Completers | 4 | |
| Mellor-Clark et al., 2013 | England | EAP | EAP/OH | 28476 | Various or Not Specific | Other | Completers | 4 | |
| Merrill et al., 2003 | USA | CMHC | Secondary | 192 | Depression | CBT | ITT | 6 | |
| Minami et al., 2008 | USA | Behavioural Health Organisation | Secondary | 5704 | Depression | Other | Modified ITT | 4 | |
| Mitsopoulou et al., 2020 | Greece | Behavioural Therapy Unit of University | Secondary | 11 | Anxiety | CBT | Completers | 6 | |
| Moorhead & Scott, 1999 | UK | Cognitive Therapy Clinic | Secondary | 20 | Various or Not Specific | CBT | Assume ITT | 5 | |
| Morley et al., 2008 | UK | Tertiary Inpatient Pain Management Service | Residential | 1013 | Health or Medically Unexplained | CBT | Completers | 6 | |
| Mullin et al., 2017 | USA | University Based Outpatient Clinic | Uni. Clinics | 75 | Various or Not Specific | Dynamic | Modified ITT | 5 | |
| Murray et al., 2016 | England | University Counselling Service | University Counselling | 305 | Various or Not Specific | Counselling | Completers | 4 | |
| Murray, 2017 | England | IAPT | Primary | NA | Various or Not Specific | CBT | Check | NA | |
| Nordmo et al., 2020 | Norway | Outpatient Services | Secondary | 370 | Various or Not Specific | Other | Modified ITT | 6 | |
| Ost et al., 2012 | Sweden | University Based Outpatient Clinic | Uni. Clinics | 591 | Various or Not Specific | CBT | ITT | 8 | |
| Owen & Hilsenroth, 2011 | USA | University Based Outpatient Clinic | Uni. Clinics | 68 | Various or Not Specific | Dynamic | Modified ITT | 5 | |
| Owen & Hilsenroth, 2014 | USA | University Based Outpatient Clinic | Uni. Clinics | 70 | Various or Not Specific | Dynamic | Modified ITT | NA | |
| Owen et al., 2015 | USA | University Counselling Centres | University Counselling | 10854 | Various or Not Specific | Other | Modified ITT | NA | |
| Paine et al., 2019 | USA | University Training Clinic In Community Psychodynamic Clinic | Uni. Clinics | 280 | Various or Not Specific | Dynamic | Modified ITT | 6 | |
| Paley et al., 2008 | England | Secondary & Tertiary Psychotherapy Services | Secondary | 67 | Various or Not Specific | Dynamic | ITT | 8 | |
| Pekarik et al., 1996 | USA | 3 CMHCs | Secondary | 152 | Various or Not Specific | Other | Completers | 6 | |
| Pereira et al., 2017 | England | IAPT | Primary | 4980 | Various or Not Specific | Other | ITT | 2 | |
| Persons et al., 1988 | USA | Private Practice | Private | 70 | Depression | CBT | Modified ITT | 7 | |
| Persons et al., 2006 | USA | Private Practice | Private | 58 | Various or Not Specific | CBT | Modified ITT | 7 | |
| Thomas et al., 2019 | USA | Private Practice | Private | 81 | Depression | CBT | Modified ITT | 3 | |
| Pfund et al., 2018 | USA | Private Practice | Private | 334 | Depression | CBT | ITT | 3 | |
| Plagge et al., 2013 | USA | Veterans Service | Veterans | 30 | Various or Not Specific | CBT | Completers | 6 | |
| Prout, 2013 | USA | University Training Clinic | Uni. Clinics | 199 | Various or Not Specific | Other | Modified ITT | 6 | |
| Puschner et al., 2007 | Germany | Psychotherapy Service | Secondary | 116 | Various or Not Specific | Dynamic | Assume ITT | 3 | |
| Puschner et al., 2007 | Germany | Psychotherapy Service | Secondary | 357 | Various or Not Specific | Dynamic | Assume ITT | 3 | |
| Pybis et al., 2017 | England | IAPT | Primary | 33243 | Depression | CBT | Modified ITT | 7 | |
| Pybis et al., 2017 | England | IAPT | Primary | NA | Depression | Counselling | Modified ITT | 7 | |
| Quarmby et al., 2007 | England | CFS Unit | Primary | 384 | Health or Medically Unexplained | CBT | Completers | 6 | |
| Rauch et al., 2009 | USA | PTSD Clinic within VA Centre | Veterans | 10 | PTSD | CBT | Completers | 5 | |
| Reese et al., 2014 | USA | Behavioural Health Organisation | Secondary | 5176 | Various or Not Specific | Other | Modified ITT | 6 | |
| Reiss et al., 2013 | USA | Inpatient | Residential | 41 | Personality Disorders | CBT | Completers | 7 | |
| Reiss et al., 2013 | USA | Inpatient | Residential | 36 | Personality Disorders | CBT | Completers | 7 | |
| Reiss et al., 2013 | Germany | Inpatient | Residential | 15 | Personality Disorders | CBT | Completers | 6 | |
| Renaud et al., 2013 | Canada | University CBT Centre | Uni. Clinics | 53 | Depression | CBT | Completers | 5 | |
| Reuber et al., 2007 | England | Specialist Psychotherapy Service with Health Service | Secondary | 63 | Health or Medically Unexplained | Dynamic | ITT | 8 | |
| Reuter et al., 2016 | Germany | Inpatient | Residential | 546 | Various or Not Specific | Other | Completers | 7 | |
| Richards et al., 2011 | England | IAPT | Primary | 219 | Various or Not Specific | CBT | Completers | 6 | |
| Ritschel et al., 2012 | USA | CMHC | Secondary | 56 | Various or Not Specific | CBT | Modified ITT | 7 | |
| Rizvi et al., 2017 | USA | DBT Training Clinic | Uni. Clinics | 50 | Personality Disorders | CBT | ITT | 8 | |
| Rocco et al., 2014 | Italy | University Outpatient Clinic | Uni. Clinics | 8 | Anxiety | Dynamic | ITT | 4 | |
| Ronnestad et al., 2019 | Norway | Private Practice | Private | 48 | Check | Other | Completers | 5 | |
| Rose & Waller, 2017 | England | IAPT | Primary | 47 | Eating Disorders | CBT | ITT | 7 | |
| Roseborough, 2006 | USA | Outpatient Mental Health Centre | Secondary | 164 | Various or Not Specific | Dynamic | Assume ITT | 6 | |
| Rosenberg et al., 2004 | USA | CMHC | Secondary | 12 | Various or Not Specific | CBT | Completers | 6 | |
| Rothbaum & Shahar, 2000 | USA | Check | Secondary | 23 | OCD | CBT | Completers | 5 | |
| Ryle & Golynkina, 2000 | England | CAT Clinic | Secondary | 27 | Personality Disorders | Other | Completers | 5 | |
| Sadock et al., 2014 | USA | University Based Ambulatory Care Clinic | Uni. Clinics | 452 | Various or Not Specific | Other | Completers | 5 | |
| Samstag & Norlander, 2019 | USA | University Counselling Centre | University Counselling | 30 | Various or Not Specific | Other | Completers | 5 | |
| Sanders et al., 2015 | USA | University Counselling Centre | University Counselling | 304 | Various or Not Specific | Other | Assume ITT | 3 | |
| Sarnholm et al., 2017 | Sweden | Cardiology Service | Primary | 19 | Health or Medically Unexplained | CBT | ITT | 6 | |
| Sauer-Zavala et al., 2019 | USA | Homeless Centre | Secondary | 4 | Various or Not Specific | CBT | ITT | 5 | |
| Scheeres et al., 2008 | Netherlands | CBT for CFS at a MHC | Veterans | 112 | Health or Medically Unexplained | CBT | ITT | 8 | |
| Schindler et al., 2011 | Germany | University Outpatient Clinic | Uni. Clinics | 338 | Depression | CBT | Modified ITT | 8 | |
| Schnicker et al., 2013 | Germany | University Outpatient Clinic | Uni. Clinics | 27 | Eating Disorders | CBT | Completers | 7 | |
| Schnicker et al., 2013 | Germany | University Outpatient Clinic | Uni. Clinics | 41 | Eating Disorders | CBT | Completers | 7 | |
| Schulz et al., 2006 | USA | Community Mental Health Agency for VA | Veterans | 53 | PTSD | CBT | Completers | 6 | |
| Schwartz, 2018 | USA | University Outpatient Clinic | Uni. Clinics | 19 | Depression | CBT | ITT | 6 | |
| Sembill et al., 2019 | Germany | University Outpatient Clinic | Uni. Clinics | 351 | Various or Not Specific | Other | Completers | 5 | |
| Shepherd et al., 2005 | England | Check | Primary | 2291 | Various or Not Specific | Other | Completers | 4 | |
| Signorini et al., 2018 | Australia | Specialist Eating Disorders Service | Secondary | 114 | Eating Disorders | CBT | ITT | 5 | |
| Simons et al., 2010 | USA | Community Mental Health Centre | Secondary | 42 | Depression | CBT | Assume ITT | 5 | |
| Simons et al., 2010 | USA | Community Mental Health Centre | Secondary | 74 | Depression | Other | Assume ITT | 5 | |
| Simpson et al., 2015 | Australia | University Outpatient Clinic | Uni. Clinics | 17 | Various or Not Specific | Other | Modified ITT | NA | |
| Slavin-Mulford et al., 2011 | USA | Community Outpatient Psychology Clinic | Uni. Clinics | 21 | Anxiety | Dynamic | Modified ITT | 7 | |
| Smith et al., 2018 | Australia | Gambling Therapy Service | Secondary | 54 | Addiction | CBT | Check | NA | |
| Smith et al., 2018 | Australia | Gambling Therapy Service | Secondary | 214 | Addiction | CBT | Check | NA | |
| Smout et al., 2019 | Australia | University Training Clinic | Uni. Clinics | 342 | Various or Not Specific | CBT | Modified ITT | 6 | |
| Steinert et al., 2015 | Germany | Inpatient Psychosomatic Service | Residential | 254 | Various or Not Specific | Dynamic | Assume ITT | 6 | |
| Steinert et al., 2019 | Germany | Inpatient & Day patient | Residential | 709 | Health or Medically Unexplained | Dynamic | Completers | 7 | |
| Stiles et al., 2003 | England | Wakefield CMHT | Secondary | 135 | Various or Not Specific | Other | Modified ITT | 8 | |
| Stiles et al., 2015 | UK | Various Sector NHS Services | Mixed | 26430 | Various or Not Specific | Other | Completers | 8 | |
| Strepparava et al., 2016 | Italy | University Counselling Centre | University Counselling | 45 | Various or Not Specific | CBT | Completers | 6 | |
| Stulz et al., 2013 | USA | UCCs, Primary Care & Private Outpatient | Mixed | 6375 | Various or Not Specific | Other | Modified ITT | 5 | |
| Swift et al., 2010 | USA | University Training Clinic | Uni. Clinics | 38 | Various or Not Specific | CBT | Assume ITT | 4 | |
| Swift et al., 2010 | USA | University Training Clinic | Uni. Clinics | 97 | Various or Not Specific | CBT | Assume ITT | 3 | |
| Talbot et al., 2005 | USA | CMHC | Secondary | 25 | Various or Not Specific | Dynamic | Completers | 4 | |
| Timmons, 2013 | USA | University Training Clinic | Uni. Clinics | 46 | Depression | Other | Completers | 5 | |
| Trockel et al., 2015 | USA | Veterans Service | Veterans | 405 | Health or Medically Unexplained | CBT | Completers | 6 | |
| Turner et al., 2015 | England | Specialist Eating Disorders Service | Secondary | 203 | Eating Disorders | CBT | ITT | 6 | |
| Tuschen-Caffier et al., 2001 | Germany | Outpatient Treatment Centre for Eating Disorders | Secondary | 66 | Eating Disorders | CBT | ITT | 4 | |
| van der Lem et al., 2012 | Netherlands | Not Clear | Secondary | 170 | Depression | Other | Modified ITT | 5 | |
| van Rijn et al., 2014 | England | Metatonia Institute | Private | NA | Various or Not Specific | Other | NA | NA | |
| van Rijn et al., 2014 | Spain | Private Practice | Private | 26 | Various or Not Specific | Other | Modified ITT | 2 | |
| van Rijn et al., 2008 | England | Low Cost Counselling Service | Private | 263 | Various or Not Specific | Other | Modified ITT | 4 | |
| Van Woudenberg et al., 2018 | Netherlands | Dutch psychotrauma expertise centre | Residential | 347 | PTSD | CBT | ITT | 6 | |
| Vermote et al., 2009 | Belgium | Hospital Based Treatment Program | Residential | 70 | Personality Disorders | Dynamic | Modified ITT | NA | |
| Vescovelli et al., 2017 | Italy | University Counselling Centre | University Counselling | 149 | Various or Not Specific | Other | Completers | 6 | |
| von Brachel et al., 2019 | Germany | University Training Clinic | Uni. Clinics | 256 | Various or Not Specific | CBT | Modified ITT | 6 | |
| Waller, et al., 2013 | England | Recovery Team & Psychosis Teams | Secondary | 12 | Psychosis | CBT | Completers | 5 | |
| Waller et al, 2014 | England | Outpatient Eating Disorders Service | Secondary | 78 | Eating Disorders | CBT | ITT | 8 | |
| Waller, et al., 2018 | England | Outpatient Eating Disorders Service | Secondary | 93 | Eating Disorders | CBT | ITT | 5 | |
| Walser et al., 2015 | USA | Veterans Service | Veterans | 981 | Depression | CBT | Completers | 4 | |
| Warren & Thomas, 2001 | USA | Private Practice | Private | 19 | OCD | CBT | Completers | 5 | |
| Wattar et al., 2005 | Denmark | Private Cognitive Psychology Clinic | Private | 16 | Anxiety | CBT | ITT | 7 | |
| Werbart et al., 2012 | Sweden | Residential Treatment for Young Adults | Residential | 56 | Various or Not Specific | Dynamic | Completers | 7 | |
| Westbrook et al., 2005 | England | Specialist CBT Clinic | Secondary | 1276 | Various or Not Specific | CBT | Completers | 7 | |
| Wilkinson et al., 2017 | USA | University Counselling Centre | University Counselling | 26 | PTSD | CBT | Modified ITT | 5 | |
| Winter et al, 2003 | England | National Network of Counselling Services | Primary | NA | Various or Not Specific | Other | Completers | 3 | |
| Wiseman, 2014 | Israel | University Counselling Centre | University Counselling | 67 | Various or Not Specific | Dynamic | Completers | 3 | |
| Wolf et al., 2018 | USA | Inpatient, Residential & Outpatient | Mixed | 44 | Various or Not Specific | CBT | ITT | 8 | |
| Worm-Smeitink et al., 2016 | England | Leading Centres for CFS | Primary | 163 | Health or Medically Unexplained | CBT | Completers | 7 | |
| Worm-Smeitink et al., 2016 | Netherlands | Leading Centres for CFS | Primary | 293 | Health or Medically Unexplained | CBT | Completers | 7 | |
| Wright & Abrahams, 2015 | England | IAPT | Primary | 26 | Various or Not Specific | Dynamic | Assume ITT | 6 | |
| Ybrandt et al., 2019 | Sweden | University Training Clinic | Uni. Clinics | 734 | Various or Not Specific | Other | Completers | 6 | |
| Young et al., 2017 | England | IAPT | Primary | 349 | Various or Not Specific | Other | Modified ITT | 6 | |
| Young et al., 2017 | England | IAPT | Primary | 85 | Various or Not Specific | Other | Modified ITT | 6 | |
| Zeeck et al., 2015 | Germany | Psychosomatic Inpatient + Day Hospitals | Mixed | 604 | Depression | Other | Completers | 7 | |
| Zieve et al., 2019 | USA | Private Practice | Private | 1092 | Various or Not Specific | CBT | Completers | 5 | |
| *Note.* |  |  |  |  |  |  |  |  |  |

# Table 5:

## Effect size data for the studies included in the meta-analysis.

|  | **Depression** | | | **Anxiety** | | | **Miscellaneous** | | |
| --- | --- | --- | --- | --- | --- | --- | --- | --- | --- |
| **Citation** | **Measure** | **N** | **d** | **Measure** | **N** | **d** | **Measure** | **N** | **d** |
| Abbass, 2002 | BDI | 101 | 1.12 | BAI | 94 | 1.04 | BSI-GSI | 89 | 1.23 |
| Abbass, 2006 | BSI (Depression) | 10 | 2.12 | No Anx Measure | NA | NA | No Miscellaneous Measure Used | NA | NA |
| Abbass et al., 2008 | BSI (Depression) | 30 | 0.62 | BSI (Anxiety) | 30 | 0.79 | BSI-GSI | 30 | 0.68 |
| Abbass, 2013 | BSI (Depression) | 23 | 0.70 | BSI (Anxiety) | 23 | 0.75 | BSI-GSI | 23 | 0.72 |
| Archer et al., 2000 | GHQ (Depression) | 89 | 0.58 | GHQ (Anxiety & Insomnia) | 89 | 0.41 | GHQ | 89 | 0.59 |
| Ashworth et al., 2015 | HADS (Depression) | 12 | 1.13 | HADS (Anxiety) | 12 | 0.98 | No Miscellaneous Measure Used | NA | NA |
| Askey-Jones et al., 2013 | HADS (Depression) | 20 | 0.99 | HADS (Anxiety) | 20 | 1.14 | No Miscellaneous Measure Used | NA | NA |
| Asnaani et al., 2020 | BDI | 453 | 0.87 | BAI | 146 | 1.22 | SDS | 442 | 1.14 |
| Back et al., 2020 | PHQ-9 | 31 | 1.52 | No Anx Measure | NA | NA | EDEQ | 31 | 1.52 |
| Back et al., 2017 | PHQ-9 | 16 | 1.68 | No Anx Measure | NA | NA | REDS | 16 | 1.22 |
| Bados et al., 2007 | DASS (Depression) | 38 | 0.82 | DASS (Anxiety) | 38 | 0.67 | No Miscellaneous Measure Used | NA | NA |
| Bales et al., 2012 | BDI | 45 | 1.19 | No Anx Measure | NA | NA | SCL (Global) | 45 | 1.36 |
| Ballesteros & Labrador, 2014 | BDI-II | 23 | 0.99 | BSQ | 15 | 1.08 | No Miscellaneous Measure Used | NA | NA |
| Beard et al., 2016 | CESD-10 | 956 | 1.60 | No Anx Measure | NA | NA | No Miscellaneous Measure Used | NA | NA |
| Ben-Porath et al., 2004 | SCL (Depression) | 23 | 0.60 | SCL (Anxiety) | 23 | 0.25 | SCL (Global) | 23 | 0.57 |
| Bjorgvinsson et al., 2014 | CESD-10 | 951 | 1.08 | PSWQ | 951 | 0.46 | SOS | 951 | 0.93 |
| Buckley et al., 2006 | BDI-II | 60 | 0.71 | No Anx Measure | NA | NA | BSI-GSI | 60 | 0.49 |
| Buckley et al., 2006 | BDI-II | 60 | 0.93 | No Anx Measure | NA | NA | BSI-GSI | 60 | 0.66 |
| Byrne et al., 2011 | DASS (Depression) | 125 | 0.40 | DASS (Anxiety) | 125 | 0.29 | EDEQ | 125 | 0.75 |
| Cahill et al., 2003 | BDI | 58 | 1.99 | No Anx Measure | NA | NA | No Miscellaneous Measure Used | NA | NA |
| Carney et al., 2011 | BDI-II | 24 | 1.41 | No Anx Measure | NA | NA | PSQI | 24 | 0.36 |
| Chase et al., 2015 | BDI-II | 134 | 1.99 | No Anx Measure | NA | NA | Y-BOCS | 134 | 1.71 |
| Christiansen et al., 2015 | CESD-10 | 85 | 0.08 | No Anx Measure | NA | NA | No Miscellaneous Measure Used | NA | NA |
| Clapp et al., 2013 | BDI | 1084 | 1.33 | No Anx Measure | NA | NA | No Miscellaneous Measure Used | NA | NA |
| Douglas et al., 2016 | PHQ-9 | 28 | 0.72 | GAD-7 | 28 | 0.76 | CORE-OM | 17 | 1.22 |
| Ehlers et al., 2013 | BDI-II | 330 | 0.97 | BAI | 330 | 1.02 | PDS | 330 | 1.88 |
| Puig et al., 2012 | BDI-II | 69 | 2.06 | No Anx Measure | NA | NA | No Miscellaneous Measure Used | NA | NA |
| Falkenstrom, 2010 | SCL (Depression) | 56 | 1.10 | SCL (Anxiety) | 56 | 0.99 | SCL (Global) | NA | NA |
| Fizke et al., 2002 | SCL (Depression) | 234 | 1.12 | No Anx Measure | NA | NA | SCL (Global) | 234 | 0.86 |
| Fizke et al., 2010 | SCL (Depression) | 514 | 1.20 | No Anx Measure | NA | NA | SCL (Global) | 514 | 1.00 |
| Forand et al., 2011 | BDI-II | 249 | 0.95 | BAI | 249 | 0.57 | No Miscellaneous Measure Used | NA | NA |
| Fortune et al., 2005 | BDI | 48 | 0.93 | BAI | 48 | 0.73 | BSI-GSI | 48 | 0.73 |
| Fowler et al., 2017 | PHQ-9 | 994 | 1.17 | GAD-7 | 994 | 1.08 | No Miscellaneous Measure Used | NA | NA |
| Fowler et al., 2018 | PHQ-9 | 245 | 1.19 | GAD-7 | 245 | 1.05 | WHO-DAS | 245 | 1.00 |
| Galili-Weinstock et al., 2018 | BDI-II | 112 | 0.29 | No Anx Measure | NA | NA | OQ-45 | 112 | 0.38 |
| Gamble et al., 2013 | BDI-II | 14 | 1.49 | No Anx Measure | NA | NA | No Miscellaneous Measure Used | NA | NA |
| Ghafoori et al., 2019 | BSI (Depression) | 88 | 0.61 | No Anx Measure | NA | NA | PCL | 88 | 1.11 |
| Ghafoori et al., 2019 | BSI (Depression) | 95 | 2.27 | No Anx Measure | NA | NA | PCL | 95 | 0.94 |
| Ghafoori et al., 2019 | BSI (Depression) | 206 | 0.86 | No Anx Measure | NA | NA | PCL | 206 | 0.95 |
| Ghafoori et al., 2019 | BSI (Depression) | 137 | 1.06 | No Anx Measure | NA | NA | PCL | 137 | 1.28 |
| Gibbons et al., 2010 | BDI-II | 217 | 1.06 | No Anx Measure | NA | NA | No Miscellaneous Measure Used | NA | NA |
| Gillespie et al., 2002 | BDI | 33 | 1.69 | No Anx Measure | NA | NA | GHQ | 37 | 1.75 |
| Gordon, 2001 | MMPI (Depression) | 55 | 1.05 | MMPI (Anxiety) | 55 | 1.19 | No Miscellaneous Measure Used | NA | NA |
| Graca et al., 2014 | BDI-II | 17 | 0.38 | BAI | 17 | -0.01 | PCL | 17 | 1.06 |
| Graca et al., 2014 | BDI-II | 17 | 0.47 | BAI | 17 | 0.27 | PCL | 17 | 1.15 |
| Hahlweg et al., 2001 | BDI | 400 | 0.92 | BAI | 399 | 1.06 | SCL (Global) | 401 | 0.83 |
| Harte & Hawkins, 2016 | BDI | 32 | 0.90 | No Anx Measure | NA | NA | No Miscellaneous Measure Used | NA | NA |
| Harte & Hawkins, 2016 | BDI | 28 | 0.85 | No Anx Measure | NA | NA | No Miscellaneous Measure Used | NA | NA |
| Hilsenroth et al., 2003 | SCL (Depression) | 20 | 1.25 | No Anx Measure | NA | NA | SCL (Global) | 20 | 0.93 |
| Hirsch et al., 2000 | BDI | 37 | 0.38 | BAI | 41 | 0.22 | No Miscellaneous Measure Used | NA | NA |
| Hirsch et al., 2000 | BDI | 32 | 0.66 | BAI | 37 | 0.82 | No Miscellaneous Measure Used | NA | NA |
| Hitt et al., 2018 | PHQ-9 | 76 | 3.08 | HADS (Anxiety) | 76 | 2.10 | WSAS | 74 | 2.15 |
| Jepsen et al., 2009 | BDI | 34 | 0.22 | SCL (Anxiety) | 34 | 0.12 | Check | 34 | 0.39 |
| Jepsen et al., 2014 | BDI-II | 56 | 0.47 | No Anx Measure | NA | NA | SCL (Global) | 56 | 0.45 |
| Jolley et al., 2015 | BDI-II | 9 | 0.16 | BAI | 9 | 0.26 | CORE-OM | 9 | 0.75 |
| Jones et al., 2012 | BDI-II | 17 | 1.24 | No Anx Measure | NA | NA | WSAS | 17 | 0.87 |
| Jordan et al., 2019 | PHQ-9 | 27 | 1.83 | GAD-7 | 27 | 1.97 | No Miscellaneous Measure Used | NA | NA |
| Karlin et al., 2019 | PHQ-9 | 36 | 1.22 | GAD-7 | 36 | 1.26 | No Miscellaneous Measure Used | NA | NA |
| Kehle et al., 2008 | BDI | 29 | 0.13 | PSWQ | 29 | 0.22 | No Miscellaneous Measure Used | NA | NA |
| Kikuchi et al., 2019 | CESD-10 | 7 | 0.89 | No Anx Measure | NA | NA | No Miscellaneous Measure Used | NA | NA |
| Knott et al., 2015 | BDI-II | 246 | 0.82 | BAI | 246 | 0.47 | EDEQ | NA | NA |
| Kobori et al., 2014 | PHQ-9 | 10 | 0.47 | GAD-7 | 10 | 0.47 | BITE-SS | 10 | 1.23 |
| Kobori, et al., 2014 | PHQ-9 | 14 | 0.81 | GAD-7 | 14 | 0.68 | OCI | 14 | 1.05 |
| Kobori et al., 2014 | PHQ-9 | 19 | 0.59 | GAD-7 | 19 | 0.59 | LSAS | 19 | 1.08 |
| Kolly et al., 2015 | BDI-II | 13 | 0.59 | No Anx Measure | NA | NA | OQ-45 | 13 | 0.09 |
| Lopez et al., 2011 | BDI | 40 | 1.20 | No Anx Measure | NA | NA | No Miscellaneous Measure Used | NA | NA |
| LoSavio et al., 2019 | PHQ-9 | 232 | 0.92 | No Anx Measure | NA | NA | PCL | 232 | 1.57 |
| Low et al., 2001 | BDI | 10 | 0.70 | No Anx Measure | NA | NA | No Miscellaneous Measure Used | NA | NA |
| Lu et al., 2009 | BDI-II | 14 | 0.37 | No Anx Measure | NA | NA | No Miscellaneous Measure Used | NA | NA |
| Marriott & Kellett, 2009 | BDI-II | 27 | 1.56 | No Anx Measure | NA | NA | BSI-GSI | 27 | 1.03 |
| Marriott & Kellett, 2009 | BDI-II | 27 | 2.30 | No Anx Measure | NA | NA | BSI-GSI | 27 | 1.39 |
| Marriott & Kellett, 2009 | BDI-II | 25 | 1.12 | No Anx Measure | NA | NA | BSI-GSI | 25 | 0.89 |
| Marriott & Kellett, 2009 | BDI-II | 38 | 1.46 | No Anx Measure | NA | NA | BSI-GSI | 38 | 0.96 |
| Marriott & Kellett, 2009 | BDI-II | 38 | 2.14 | No Anx Measure | NA | NA | BSI-GSI | 38 | 1.89 |
| Marriott & Kellett, 2009 | BDI-II | 38 | 1.24 | No Anx Measure | NA | NA | BSI-GSI | 38 | 0.76 |
| McAleavey et al., 2019 | CCAPS (Depression) | 9895 | 0.64 | CCAPS (Anxiety) | 9895 | 0.46 | No Miscellaneous Measure Used | NA | NA |
| McBride et al., 2010 | BDI-II | 74 | 1.64 | No Anx Measure | NA | NA | No Miscellaneous Measure Used | NA | NA |
| McDevitt-Petrovic et al., 2018 | PHQ-9 | 163 | 1.29 | GAD-7 | 163 | 1.74 | No Miscellaneous Measure Used | NA | NA |
| McEvoy et al., 2014 | BDI-II | 85 | 1.03 | BAI | 83 | 0.87 | No Miscellaneous Measure Used | NA | NA |
| Merrill et al., 2003 | BDI | 160 | 1.41 | No Anx Measure | NA | NA | No Miscellaneous Measure Used | NA | NA |
| Mitsopoulou et al., 2020 | BDI-II | 11 | 0.93 | BAI | 11 | 1.14 | WSAS | 11 | 1.19 |
| Morley et al., 2008 | BDI | 833 | 0.63 | HADS (Anxiety) | 720 | 0.48 | PSEQ | 831 | 0.82 |
| Ost et al., 2012 | BDI | 546 | 0.74 | BAI | 546 | 0.98 | No Miscellaneous Measure Used | NA | NA |
| Paine et al., 2019 | TOP (Depression) | 269 | 0.44 | TOP (Anxiety) | 269 | 0.40 | No Miscellaneous Measure Used | NA | NA |
| Paley et al., 2008 | BDI-II | 62 | 0.88 | No Anx Measure | NA | NA | CORE-OM | 62 | 0.92 |
| Pereira et al., 2017 | PHQ-9 | 4980 | 0.57 | GAD-7 | 4976 | 0.55 | WSAS | 4980 | 0.36 |
| Persons et al., 1988 | BDI | 70 | 1.38 | No Anx Measure | NA | NA | No Miscellaneous Measure Used | NA | NA |
| Persons et al., 2006 | BDI | 38 | 1.40 | Burns AS | 56 | 0.96 | No Miscellaneous Measure Used | NA | NA |
| Thomas et al., 2019 | BDI | 82 | 1.93 | No Anx Measure | NA | NA | No Miscellaneous Measure Used | NA | NA |
| Pfund et al., 2018 | BDI-II | 334 | 0.70 | No Anx Measure | NA | NA | No Miscellaneous Measure Used | NA | NA |
| Plagge et al., 2013 | PHQ-9 | 30 | 0.82 | No Anx Measure | NA | NA | PCL | 30 | 0.84 |
| Pybis et al., 2017 | PHQ-9 | 23595 | 0.94 | No Anx Measure | NA | NA | No Miscellaneous Measure Used | NA | NA |
| Pybis et al., 2017 | PHQ-9 | 9648 | 0.95 | No Anx Measure | NA | NA | No Miscellaneous Measure Used | NA | NA |
| Rauch et al., 2009 | BDI-II | 7 | 1.06 | No Anx Measure | NA | NA | Check | 10 | 2.14 |
| Renaud et al., 2013 | BDI-II | 51 | 1.25 | No Anx Measure | NA | NA | No Miscellaneous Measure Used | NA | NA |
| Richards et al., 2011 | PHQ-9 | 219 | 1.19 | GAD-7 | 219 | 1.31 | No Miscellaneous Measure Used | NA | NA |
| Ritschel et al., 2012 | BDI | 55 | 0.47 | SAS | 56 | 0.37 | No Miscellaneous Measure Used | NA | NA |
| Rizvi et al., 2017 | BDI-II | 50 | 0.90 | No Anx Measure | NA | NA | BSI-GSI | 50 | 1.10 |
| Rocco et al., 2014 | SCL (Depression) | 8 | 0.44 | SCL (Anxiety) | 8 | 1.76 | SCL (Global) | 8 | 1.76 |
| Rose & Waller, 2017 | PHQ-9 | 47 | 1.09 | No Anx Measure | NA | NA | EDEQ | 47 | 0.38 |
| Ryle & Golynkina, 2000 | BDI | 26 | 0.76 | No Anx Measure | NA | NA | SCL (Global) | 27 | 0.63 |
| Sadock et al., 2014 | PHQ-9 | 26 | 0.33 | GAD-7 | 10 | 0.80 | No Miscellaneous Measure Used | NA | NA |
| Sarnholm et al., 2017 | PHQ-9 | 19 | 0.60 | GAD-7 | 19 | 0.75 | No Miscellaneous Measure Used | NA | NA |
| Sauer-Zavala et al., 2019 | PHQ-9 | 6 | -0.23 | GAD-7 | 6 | 0.43 | No Miscellaneous Measure Used | NA | NA |
| Schindler et al., 2011 | BDI | 338 | 1.42 | No Anx Measure | NA | NA | No Miscellaneous Measure Used | NA | NA |
| Schnicker et al., 2013 | BDI | 21 | 1.05 | BSI (Anxiety) | 17 | 0.88 | BSI-GSI | 23 | 0.64 |
| Schnicker et al., 2013 | BDI | 28 | 2.07 | BSI (Anxiety) | 21 | 1.13 | BSI-GSI | 32 | 1.14 |
| Schwartz, 2018 | PHQ-9 | 19 | 1.03 | No Anx Measure | NA | NA | No Miscellaneous Measure Used | NA | NA |
| Signorini et al., 2018 | DASS (Depression) | 111 | 0.34 | DASS (Anxiety) | 111 | 0.24 | EDEQ | 108 | 0.72 |
| Simons et al., 2010 | BDI-II | 42 | NA | BAI | 42 | NA | No Miscellaneous Measure Used | NA | NA |
| Simons et al., 2010 | BDI-II | 74 | NA | BAI | 74 | NA | No Miscellaneous Measure Used | NA | NA |
| Slavin-Mulford et al., 2011 | BSI (Depression) | 21 | 0.66 | BSI (Anxiety) | 21 | 0.89 | BSI-GSI | 21 | 0.82 |
| Smout et al., 2019 | BDI-II | 342 | 0.90 | PSWQ | 93 | 1.04 | K10 | 210 | 0.89 |
| Stiles et al., 2003 | BDI | 119 | 1.12 | BAI | 117 | 0.68 | CORE-OM | 135 | 0.83 |
| Strepparava et al., 2016 | SCL (Depression) | 45 | 0.50 | SCL (Anxiety) | 45 | 0.59 | CORE-OM | 45 | 0.86 |
| Talbot et al., 2005 | BDI-II | 25 | 1.15 | No Anx Measure | NA | NA | SAS-SR | 25 | 0.49 |
| Trockel et al., 2015 | BDI-II | 405 | 0.59 | No Anx Measure | NA | NA | ISI | 405 | 2.25 |
| Turner et al., 2015 | HADS (Depression) | 116 | 0.51 | HADS (Anxiety) | 116 | 0.38 | No Miscellaneous Measure Used | NA | NA |
| van Rijn et al., 2014 | PHQ-9 | 263 | 0.68 | Check | 263 | 0.77 | CORE 10 | 263 | 0.75 |
| von Brachel et al., 2019 | BDI | 193 | 0.88 | No Anx Measure | NA | NA | BSI-GSI | 216 | 0.63 |
| Waller, et al., 2013 | CESD-10 | 12 | 0.89 | No Anx Measure | NA | NA | CORE-OM | 12 | 1.02 |
| Waller et al, 2014 | BDI-II | 78 | 1.03 | No Anx Measure | NA | NA | EDI | 78 | 0.61 |
| Waller, et al., 2018 | PHQ-9 | 93 | 0.91 | GAD-7 | 93 | 0.86 | EDEQ | 93 | 1.65 |
| Walser et al., 2015 | BDI-II | 981 | 1.01 | No Anx Measure | NA | NA | No Miscellaneous Measure Used | NA | NA |
| Westbrook et al., 2005 | BDI | 776 | 0.68 | BAI | 423 | 0.54 | No Miscellaneous Measure Used | NA | NA |
| Wolf et al., 2018 | BDI-II | 44 | 1.41 | No Anx Measure | NA | NA | PCL | 44 | 2.89 |
| Wright & Abrahams, 2015 | PHQ-9 | 24 | 0.46 | GAD-7 | 24 | 0.65 | No Miscellaneous Measure Used | NA | NA |
| Young et al., 2017 | PHQ-9 | 429 | 1.16 | GAD-7 | 428 | 1.10 | No Miscellaneous Measure Used | NA | NA |
| Young et al., 2017 | PHQ-9 | 116 | 1.03 | GAD-7 | 116 | 0.87 | No Miscellaneous Measure Used | NA | NA |
| Zieve et al., 2019 | BDI | 410 | 0.72 | Burns AS | 358 | 0.68 | No Miscellaneous Measure Used | NA | NA |
| Aazh & Moore, 2018 | No Depression Measure Used | NA | NA | No Anx Measure | NA | NA | ISI | 55 | 0.57 |
| Abbass et al., 2009 | No Depression Measure Used | NA | NA | No Anx Measure | NA | NA | BSI-GSI | 23 | 0.58 |
| Abramowitz et al., 2003 | No Depression Measure Used | NA | NA | No Anx Measure | NA | NA | Y-BOCS | 132 | 3.07 |
| Adamson et al., 2020 | HADS (Depression) | NA | NA | HADS (Anxiety) | NA | NA | WSAS | 989 | 0.94 |
| Andrews et al., 2011 | No Depression Measure Used | NA | NA | No Anx Measure | NA | NA | CORE (10/OM) | 108 | 1.49 |
| Andrews et al., 2013 | No Depression Measure Used | NA | NA | No Anx Measure | NA | NA | CORE-10 | 3885 | 1.21 |
| Armstrong, 2010 | No Depression Measure Used | NA | NA | No Anx Measure | NA | NA | CORE-OM | 118 | 0.71 |
| Asay et al., 2002 | No Depression Measure Used | NA | NA | No Anx Measure | NA | NA | OQ-45 | 28 | NA |
| Baldwin et al., 2009 | No Depression Measure Used | NA | NA | No Anx Measure | NA | NA | OQ-45 | 4676 | 0.66 |
| Banham & Schweitzer, 2016 | No Depression Measure Used | NA | NA | No Anx Measure | NA | NA | OQ-45 | NA | NA |
| Barkham et al., 2001 | No Depression Measure Used | NA | NA | No Anx Measure | NA | NA | CORE-OM | 224 | 0.87 |
| Barkham et al., 2012 | No Depression Measure Used | NA | NA | No Anx Measure | NA | NA | CORE-OM | 16145 | 0.81 |
| Barnitcot & Crawford, 2018 | No Depression Measure Used | NA | NA | No Anx Measure | NA | NA | BEST | 58 | 0.98 |
| Barnitcot & Crawford, 2018 | No Depression Measure Used | NA | NA | No Anx Measure | NA | NA | BEST | 32 | 0.45 |
| Baronian & Leggett, 2020 | No Depression Measure Used | NA | NA | No Anx Measure | NA | NA | CORE-OM | 53 | 1.27 |
| Beail et al., 2005 | No Depression Measure Used | NA | NA | No Anx Measure | NA | NA | BSI-GSI | 20 | 0.54 |
| Birtchnell et al., 2005 | No Depression Measure Used | NA | NA | No Anx Measure | NA | NA | CORE-OM | 31 | 0.73 |
| Bitran et al., 2008 | No Depression Measure Used | NA | NA | PSDD | 32 | 1.84 | No Miscellaneous Measure Used | NA | NA |
| Blainey et al., 2017 | No Depression Measure Used | NA | NA | No Anx Measure | NA | NA | CORE-OM | 81 | 0.39 |
| Boettcher et al., 2019 | BDI-II | 105 | 1.01 | SPS | 105 | 0.98 | BSI-GSI | 105 | 1.11 |
| Bradshaw et al., 2009 | No Depression Measure Used | NA | NA | No Anx Measure | NA | NA | OQ-45 | 78 | 0.84 |
| Brand, 2020 | No Depression Measure Used | NA | NA | No Anx Measure | NA | NA | CORE-OM | 2151 | 0.92 |
| Briggie et al., 2016 | No Depression Measure Used | NA | NA | No Anx Measure | NA | NA | BSI-GSI | 243 | 0.51 |
| Brunnbauer et al., 2016 | No Depression Measure Used | NA | NA | No Anx Measure | NA | NA | CORE-10 | 74 | 0.90 |
| Budge et al., 2013 | No Depression Measure Used | NA | NA | No Anx Measure | NA | NA | BHM | 169 | 0.60 |
| Budge et al., 2013 | No Depression Measure Used | NA | NA | No Anx Measure | NA | NA | BHM | 33 | 0.82 |
| Budge et al., 2013 | No Depression Measure Used | NA | NA | No Anx Measure | NA | NA | BHM | 403 | 0.76 |
| Budge et al., 2013 | No Depression Measure Used | NA | NA | No Anx Measure | NA | NA | BHM | 713 | 0.67 |
| Buckley et al., 2016 | No Depression Measure Used | NA | NA | No Anx Measure | NA | NA | CORE 10 | NA | NA |
| Burlingame et al., 2016 | No Depression Measure Used | NA | NA | No Anx Measure | NA | NA | OQ-45 | 1557 | NA |
| Burlingame et al., 2016 | No Depression Measure Used | NA | NA | No Anx Measure | NA | NA | OQ-45 | 11764 | NA |
| Butler et al., 2020 | No Depression Measure Used | NA | NA | SIAS-S | 93 | 0.87 | No Miscellaneous Measure Used | NA | NA |
| Callahan et al., 2005 | No Depression Measure Used | NA | NA | No Anx Measure | NA | NA | OQ-45 | 61 | NA |
| Carr et al., 2017 | No Depression Measure Used | NA | NA | No Anx Measure | NA | NA | OQ-45 | 132 | NA |
| Carter, 2005 | No Depression Measure Used | NA | NA | No Anx Measure | NA | NA | GHQ | NA | NA |
| Connell et al., 2008 | No Depression Measure Used | NA | NA | No Anx Measure | NA | NA | CORE-OM | 323 | 1.55 |
| Cooper et al., 2017 | No Depression Measure Used | NA | NA | No Anx Measure | NA | NA | PHQ-15 | 37 | 0.36 |
| Daig et al., 2009 | No Depression Measure Used | NA | NA | No Anx Measure | NA | NA | BMM | 992 | 0.60 |
| Davis et al., 2008 | No Depression Measure Used | NA | NA | No Anx Measure | NA | NA | CORE-OM | 58 | 0.97 |
| Davis et al., 2010 | BDI-II | NA | NA | DASS (Anxiety) | NA | NA | WSAS | NA | NA |
| de Jongh et al., 2011 | No Depression Measure Used | NA | NA | No Anx Measure | NA | NA | HADS | 125 | 1.13 |
| de Jongh et al., 2011 | No Depression Measure Used | NA | NA | No Anx Measure | NA | NA | HADS | 59 | 1.12 |
| Delgadillo et al., 2020 | PHQ-9 | 1435 | NA | No Anx Measure | NA | NA | No Miscellaneous Measure Used | NA | NA |
| Dennhag & Armelius, 2012 | No Depression Measure Used | NA | NA | No Anx Measure | NA | NA | SCL | 187 | 0.60 |
| Dickson & Gullo, 2015 | PHQ-9 | 48 | NA | GAD-7 | 48 | NA | No Miscellaneous Measure Used | NA | NA |
| Doorn et al., 2014 | No Depression Measure Used | NA | NA | No Anx Measure | NA | NA | CORE-OM | 31 | 2.36 |
| Ellison et al., 2013 | No Depression Measure Used | NA | NA | No Anx Measure | NA | NA | TOP | NA | NA |
| Erekson et al., 2013 | No Depression Measure Used | NA | NA | No Anx Measure | NA | NA | OQ-45 | 3092 | NA |
| Erekson et al., 2013 | No Depression Measure Used | NA | NA | No Anx Measure | NA | NA | OQ-45 | 3092 | NA |
| Evans et al., 2017 | No Depression Measure Used | NA | NA | No Anx Measure | NA | NA | CORE-OM | 925 | 0.79 |
| Falkenstein et al., 2019 | HRSD-6 | NA | NA | No Anx Measure | NA | NA | YBOCS | NA | NA |
| Flo et al., 2014 | HADS (Depression) | NA | NA | HADS (Anxiety) | NA | NA | WSAS | NA | NA |
| Flygare et al., 2020 | No Depression Measure Used | NA | NA | No Anx Measure | NA | NA | YBOCS | 19 | 1.98 |
| Fox et al., 2015 | No Depression Measure Used | NA | NA | No Anx Measure | NA | NA | ZAN-BPD | 18 | 1.28 |
| Frueh et al., 2009 | No Depression Measure Used | NA | NA | No Anx Measure | NA | NA | SF-36 | 13 | 0.96 |
| Ghilardia et al., 2018 | No Depression Measure Used | NA | NA | No Anx Measure | NA | NA | GHQ | 39 | 1.61 |
| Gibbard & Hanley, 2008 | No Depression Measure Used | NA | NA | No Anx Measure | NA | NA | CORE-OM | 697 | 1.24 |
| Gilbert et al., 2005 | No Depression Measure Used | NA | NA | No Anx Measure | NA | NA | CORE-OM | 553 | 1.35 |
| Gimeno-Peon et al., 2019 | No Depression Measure Used | NA | NA | No Anx Measure | NA | NA | ORS | 42 | 1.45 |
| Goldberg et al., 2016 | No Depression Measure Used | NA | NA | No Anx Measure | NA | NA | OQ-45 | 6591 | 0.94 |
| Greasley & Small, 2005 | No Depression Measure Used | NA | NA | No Anx Measure | NA | NA | CORE-OM | 16 | 0.11 |
| Gropalis et al., 2012 | No Depression Measure Used | NA | NA | BSI (Anxiety) | 224 | 0.62 | BSI-GSI | 224 | 0.58 |
| Gropalis et al., 2012 | No Depression Measure Used | NA | NA | BSI (Anxiety) | 65 | 0.44 | BSI-GSI | 65 | 0.44 |
| Gropalis et al., 2012 | No Depression Measure Used | NA | NA | BSI (Anxiety) | 94 | 0.50 | BSI-GSI | 94 | 0.72 |
| Guthrie et al., 2004 | No Depression Measure Used | NA | NA | No Anx Measure | NA | NA | CORE-OM | 41 | 1.29 |
| Haase et al., 2008 | SCL (Depression) | 158 | 1.22 | SCL (Anxiety) | 158 | 1.00 | SCL (Global) | 158 | 1.16 |
| Halje et al., 2015 | HADS (Depression) | 607 | 0.58 | HADS (Anxiety) | 607 | 0.58 | GHQ | 604 | 0.64 |
| Harnett et al., 2010 | No Depression Measure Used | NA | NA | No Anx Measure | NA | NA | OQ-45 | 125 | NA |
| Harte & Hawkins, 2016 | No Depression Measure Used | NA | NA | PSWQ | 22 | 1.80 | No Miscellaneous Measure Used | NA | NA |
| Harte & Hawkins, 2016 | No Depression Measure Used | NA | NA | PSWQ | 17 | 2.03 | No Miscellaneous Measure Used | NA | NA |
| Harte & Hawkins, 2016 | No Depression Measure Used | NA | NA | No Anx Measure | NA | NA | Y-BOCS | 20 | 1.13 |
| Harte & Hawkins, 2016 | No Depression Measure Used | NA | NA | No Anx Measure | NA | NA | Y-BOCS | 4 | -0.57 |
| Harte & Hawkins, 2016 | No Depression Measure Used | NA | NA | PAI | 53 | 1.02 | No Miscellaneous Measure Used | NA | NA |
| Harte & Hawkins, 2016 | No Depression Measure Used | NA | NA | PAI | 22 | 1.15 | No Miscellaneous Measure Used | NA | NA |
| Harte & Hawkins, 2016 | No Depression Measure Used | NA | NA | BAI | 14 | 1.27 | No Miscellaneous Measure Used | NA | NA |
| Harte & Hawkins, 2016 | No Depression Measure Used | NA | NA | BAI | 5 | 0.30 | No Miscellaneous Measure Used | NA | NA |
| Harte & Hawkins, 2016 | No Depression Measure Used | NA | NA | ASC | 50 | 1.67 | No Miscellaneous Measure Used | NA | NA |
| Harte & Hawkins, 2016 | No Depression Measure Used | NA | NA | ASC | 38 | 2.19 | No Miscellaneous Measure Used | NA | NA |
| Haugen et al., 2017 | No Depression Measure Used | NA | NA | No Anx Measure | NA | NA | OQ-45 | 32 | 0.59 |
| Heins et al., 2011 | No Depression Measure Used | NA | NA | No Anx Measure | NA | NA | SCL (Global) | 80 | NA |
| Heins et al., 2011 | No Depression Measure Used | NA | NA | No Anx Measure | NA | NA | SCL (Global) | 136 | NA |
| Hill et al., 2015 | No Depression Measure Used | NA | NA | No Anx Measure | NA | NA | OQ-45 | NA | NA |
| Hiltunen et al., 2013 | No Depression Measure Used | NA | NA | No Anx Measure | NA | NA | SCL | NA | NA |
| Hiney-Saunders et al., 2019 | No Depression Measure Used | NA | NA | No Anx Measure | NA | NA | EDEQ | 44 | 1.33 |
| Holmqvist et al., 2013 | No Depression Measure Used | NA | NA | No Anx Measure | NA | NA | CORE-OM | 667 | 1.36 |
| Houghton et al., 2010 | No Depression Measure Used | NA | NA | No Anx Measure | NA | NA | YBOCS | 37 | 1.20 |
| Jakupcak et al., 2010 | BDI-II | 6 | 1.28 | No Anx Measure | NA | NA | PCL | 6 | 1.87 |
| Jankowski et al., 2019 | No Depression Measure Used | NA | NA | No Anx Measure | NA | NA | TOP | NA | NA |
| Jenkins et al., 2019 | No Depression Measure Used | NA | NA | No Anx Measure | NA | NA | CORE-OM | 63 | 1.29 |
| Johansson et al., 2014 | No Depression Measure Used | NA | NA | No Anx Measure | NA | NA | SCL (Global) | 412 | 0.91 |
| Jolley et al., 2015 | No Depression Measure Used | NA | NA | No Anx Measure | NA | NA | CORE-10 | 45 | 0.57 |
| Jones et al., 2008 | No Depression Measure Used | NA | NA | No Anx Measure | NA | NA | CORE-OM | NA | NA |
| Kaplinski, 2014 | No Depression Measure Used | NA | NA | No Anx Measure | NA | NA | OQ-45 | 213 | 0.44 |
| Kellett et al., 2013 | No Depression Measure Used | NA | NA | No Anx Measure | NA | NA | CORE-OM | 17 | 0.75 |
| Knoop et al., 2007 | No Depression Measure Used | NA | NA | No Anx Measure | NA | NA | SF-36 | 96 | 1.27 |
| Kramer et al., 2013 | No Depression Measure Used | NA | NA | No Anx Measure | NA | NA | OQ-45 | 13 | 0.63 |
| Kvarsteinet al., 2014 | No Depression Measure Used | NA | NA | No Anx Measure | NA | NA | BSI-GSI | 64 | 1.48 |
| Lambert et al., 2001 | No Depression Measure Used | NA | NA | No Anx Measure | NA | NA | OQ-45 | NA | NA |
| Levitt et al., 2007 | BDI | 59 | 0.73 | No Anx Measure | NA | NA | SAS | 59 | 0.54 |
| Liness et al., 2019 | PHQ-9 | 488 | 1.80 | GAD-7 | 1089 | 1.38 | OCI | 191 | 1.46 |
| Lopez & Basco, 2015 | BDI-II | 83 | 1.00 | No Anx Measure | NA | NA | No Miscellaneous Measure Used | NA | NA |
| Lunnen et al., 2008 | No Depression Measure Used | NA | NA | No Anx Measure | NA | NA | OQ-45 | 66 | -0.04 |
| Lutz et al., 2002 | No Depression Measure Used | NA | NA | No Anx Measure | NA | NA | GHQ | NA | NA |
| Lutz et al., 2016 | No Depression Measure Used | NA | NA | No Anx Measure | NA | NA | BSI-GSI | 574 | 0.88 |
| Mayy, 1984 | No Depression Measure Used | NA | NA | No Anx Measure | NA | NA | PAL-C | NA | NA |
| McHugh et al., 2014 | No Depression Measure Used | NA | NA | No Anx Measure | NA | NA | CORE-OM | 31 | 0.83 |
| McHugh et al., 2016 | No Depression Measure Used | NA | NA | No Anx Measure | NA | NA | CORE-OM | 48 | 1.35 |
| McKenzie & Marks, 2003 | No Depression Measure Used | NA | NA | No Anx Measure | NA | NA | Y-BOCS | 51 | 1.27 |
| McLeod et al., 2000 | No Depression Measure Used | NA | NA | No Anx Measure | NA | NA | MHI-5 | 235 | 0.69 |
| McLeod et al., 2000 | No Depression Measure Used | NA | NA | No Anx Measure | NA | NA | MHI-5 | 262 | 0.96 |
| Mellor-Clark et al., 2013 | No Depression Measure Used | NA | NA | No Anx Measure | NA | NA | CORE-OM | 17520 | 1.43 |
| Minami et al., 2008 | No Depression Measure Used | NA | NA | No Anx Measure | NA | NA | OQ-30 | 5704 | 0.74 |
| Moorhead & Scott, 1999 | BDI | 20 | 1.34 | STAI | 20 | 0.93 | No Miscellaneous Measure Used | NA | NA |
| Mullin et al., 2017 | No Depression Measure Used | NA | NA | No Anx Measure | NA | NA | SCL (Global) | 75 | 0.77 |
| Murray et al., 2016 | No Depression Measure Used | NA | NA | No Anx Measure | NA | NA | CORE-OM | 305 | 1.24 |
| Murray, 2017 | PHQ-9 | NA | NA | GAD-7 | NA | NA | IES-R | NA | NA |
| Nordmo et al., 2020 | No Depression Measure Used | NA | NA | No Anx Measure | NA | NA | SCL (Global) | 362 | 0.85 |
| Owen & Hilsenroth, 2011 | No Depression Measure Used | NA | NA | No Anx Measure | NA | NA | SCL (Global) | 68 | 1.19 |
| Owen & Hilsenroth, 2014 | No Depression Measure Used | NA | NA | No Anx Measure | NA | NA | BSI-GSI | NA | NA |
| Owen et al., 2015 | No Depression Measure Used | NA | NA | No Anx Measure | NA | NA | BHM | NA | NA |
| Pekarik et al., 1996 | No Depression Measure Used | NA | NA | No Anx Measure | NA | NA | BSI-GSI | NA | NA |
| Prout, 2013 | No Depression Measure Used | NA | NA | No Anx Measure | NA | NA | OQ-45 | 199 | 0.71 |
| Puschner et al., 2007 | No Depression Measure Used | NA | NA | No Anx Measure | NA | NA | Check | NA | NA |
| Puschner et al., 2007 | No Depression Measure Used | NA | NA | No Anx Measure | NA | NA | Check | NA | NA |
| Quarmby et al., 2007 | No Depression Measure Used | NA | NA | No Anx Measure | NA | NA | WSAS | NA | NA |
| Reese et al., 2014 | No Depression Measure Used | NA | NA | No Anx Measure | NA | NA | ORS | 5176 | 0.71 |
| Reiss et al., 2013 | No Depression Measure Used | NA | NA | No Anx Measure | NA | NA | SCL (Global) | 41 | 2.36 |
| Reiss et al., 2013 | No Depression Measure Used | NA | NA | No Anx Measure | NA | NA | SCL (Global) | 36 | 1.70 |
| Reiss et al., 2013 | No Depression Measure Used | NA | NA | No Anx Measure | NA | NA | BSL | 15 | 0.72 |
| Reuber et al., 2007 | No Depression Measure Used | NA | NA | No Anx Measure | NA | NA | CORE-OM | 63 | 0.33 |
| Reuter et al., 2016 | No Depression Measure Used | NA | NA | No Anx Measure | NA | NA | SCL (Global) | 546 | 1.20 |
| Ronnestad et al., 2019 | No Depression Measure Used | NA | NA | No Anx Measure | NA | NA | OQ-45 | 48 | 0.93 |
| Roseborough, 2006 | No Depression Measure Used | NA | NA | No Anx Measure | NA | NA | OQ-45 | 164 | 0.41 |
| Rosenberg et al., 2004 | No Depression Measure Used | NA | NA | No Anx Measure | NA | NA | PCL | 13 | 0.96 |
| Rothbaum & Shahar, 2000 | No Depression Measure Used | NA | NA | No Anx Measure | NA | NA | Y-BOCS | 23 | 2.43 |
| Samstag & Norlander, 2019 | No Depression Measure Used | NA | NA | No Anx Measure | NA | NA | SCL (Global) | 30 | 0.62 |
| Sanders et al., 2015 | No Depression Measure Used | NA | NA | No Anx Measure | NA | NA | CA-COM (Distress) | 304 | 1.10 |
| Scheeres et al., 2008 | No Depression Measure Used | NA | NA | No Anx Measure | NA | NA | SF-36 | 112 | 0.67 |
| Schulz et al., 2006 | No Depression Measure Used | NA | NA | No Anx Measure | NA | NA | PSS | 53 | 3.29 |
| Sembill et al., 2019 | No Depression Measure Used | NA | NA | No Anx Measure | NA | NA | FEP | 313 | 1.09 |
| Shepherd et al., 2005 | No Depression Measure Used | NA | NA | No Anx Measure | NA | NA | CORE-OM | 458 | 1.17 |
| Simpson et al., 2015 | No Depression Measure Used | NA | NA | No Anx Measure | NA | NA | CORE-10 | NA | NA |
| Smith et al., 2018 | No Depression Measure Used | NA | NA | No Anx Measure | NA | NA | K10 | NA | NA |
| Smith et al., 2018 | No Depression Measure Used | NA | NA | No Anx Measure | NA | NA | K10 | NA | NA |
| Steinert et al., 2015 | No Depression Measure Used | NA | NA | No Anx Measure | NA | NA | SCL (Global) | 254 | 0.90 |
| Steinert et al., 2019 | No Depression Measure Used | NA | NA | No Anx Measure | NA | NA | SCL (Global) | 709 | 1.00 |
| Stiles et al., 2015 | No Depression Measure Used | NA | NA | No Anx Measure | NA | NA | CORE-OM | 26430 | 1.89 |
| Stulz et al., 2013 | No Depression Measure Used | NA | NA | No Anx Measure | NA | NA | BHM | 6375 | 1.26 |
| Swift et al., 2010 | No Depression Measure Used | NA | NA | No Anx Measure | NA | NA | OQ-45 | NA | NA |
| Swift et al., 2010 | No Depression Measure Used | NA | NA | No Anx Measure | NA | NA | OQ-45 | NA | NA |
| Timmons, 2013 | BDI | 46 | 0.96 | No Anx Measure | NA | NA | No Miscellaneous Measure Used | NA | NA |
| Tuschen-Caffier et al., 2001 | BDI-II | 71 | 0.64 | HADS (Anxiety) | 116 | 0.37 | CORE-OM | 110 | 0.61 |
| van der Lem et al., 2012 | BDI-II | 170 | 0.85 | No Anx Measure | NA | NA | No Miscellaneous Measure Used | NA | NA |
| van Rijn et al., 2014 | PHQ-9 | NA | NA | GAD-7 | NA | NA | CORE 10 | NA | NA |
| van Rijn et al., 2008 | No Depression Measure Used | NA | NA | No Anx Measure | NA | NA | CORE-OM | NA | NA |
| Van Woudenberg et al., 2018 | No Depression Measure Used | NA | NA | No Anx Measure | NA | NA | PSS | 315 | 2.09 |
| Vermote et al., 2009 | BDI | NA | NA | STAI | NA | NA | SCL-90 | NA | NA |
| Vescovelli et al., 2017 | No Depression Measure Used | NA | NA | No Anx Measure | NA | NA | CORE-OM | 149 | 1.03 |
| Warren & Thomas, 2001 | No Depression Measure Used | NA | NA | No Anx Measure | NA | NA | Y-BOCS | 19 | 1.94 |
| Wattar et al., 2005 | BDI | 16 | NA | BAI | 16 | NA | No Miscellaneous Measure Used | NA | NA |
| Werbart et al., 2012 | No Depression Measure Used | NA | NA | No Anx Measure | NA | NA | SCL (Global) | 14 | 1.37 |
| Wilkinson et al., 2017 | PHQ-9 | 26 | 0.48 | No Anx Measure | NA | NA | PCL | 26 | 1.15 |
| Winter et al, 2003 | No Depression Measure Used | NA | NA | No Anx Measure | NA | NA | CORE-OM | 232 | 1.06 |
| Wiseman, 2014 | No Depression Measure Used | NA | NA | No Anx Measure | NA | NA | OQ-45 | 58 | 0.86 |
| Worm-Smeitink et al., 2016 | No Depression Measure Used | NA | NA | No Anx Measure | NA | NA | WSAS | 129 | 0.66 |
| Worm-Smeitink et al., 2016 | No Depression Measure Used | NA | NA | No Anx Measure | NA | NA | WSAS | 217 | 1.82 |
| Ybrandt et al., 2019 | No Depression Measure Used | NA | NA | No Anx Measure | NA | NA | CORE-OM | 734 | 1.07 |
| Zeeck et al., 2015 | No Depression Measure Used | NA | NA | No Anx Measure | NA | NA | SCL (Global) | 478 | 0.90 |
| *Note.* Abbreviations:Beck’s Depression Inventory (BDI); Patient Health Questionnaire-9 (PHQ-9); Brief Symptom Invetory (BSI); Symptom Checklist 90 Revised (SCL90R); Centre for Epidemiological Studies Depression Scale (CESD10); Depression Anxiety and Stress Scale (DASS); Hospial Anxiety & Depression Scale (HADS) Short Form-36 (SF36); Beck’s Anxiety Inventory BAI); Miscellaneous ised Anxeity Disorder-7 (GAD7); Penn-State Worry Questionnaire (PSWQ); CORE Outcome Measurement (CORE-OM); Outcome Questionnaire-45 (OQ45); PTSD Checklist (PCL); Work and Social Adjustment Scale (WSAS); Yale-Brown Obsessive Compulsive Scale (Y-BOCS); Eating Disorder Examination Questionnaire (EDEQ); Behavioural Health Measure (BHM); Miscellaneous Health Questionnaire (GHQ); Short Form-36 (SF36); Appraisal of Social Concerns Scale (ASC); Counseling Center Assessment of Psychological Symptoms (CCAPS Anxiety Index); Burns Anxiety Inventory (Burns AI); Minnesota Multi-Phasic Inventory (MMPI-Anxiety); Panic Appraisal Inventory (PAI); Panic Disorder Severity Scale (PDSS); Social Interaction Anxiety Scale (SIAS); Social Phobia Scale (SPS); State-Trait Anxiety Inventory (STAI); Zung Self-Rating Anxiety Scale (SAS) | | | | | | | | | |

| Table 6 **Findings from the primary meta-analyses.** | | | | | | |  |
| --- | --- | --- | --- | --- | --- | --- | --- |
|  | **k** | **ES** | **Lower** | **Upper** | **p** | **I2** | **Q** |
| Depression | 140 | 0.98 | 0.90 | 1.06 | < 0.001 | 98.40 | 3037.46 |
| Anxiety | 84 | 0.83 | 0.73 | 0.92 | < 0.001 | 97.52 | 1488.88 |
| Miscellaneous | 184 | 1.01 | 0.93 | 1.08 | < 0.001 | 98.92 | 15685.18 |

# *Bibliography*

*Aazh, H., & Moore, B. C. J. (2018). Effectiveness of audiologist-delivered cognitive behavioral therapy for tinnitus and hyperacusis rehabilitation: Outcomes for patients treated in routine practice. *American Journal of Audiology*, *27*(4), 547–558. https://doi.org/10.1044/2018_AJA-17-0096

*Abbass, Allan A. (2002). Intensive short-term dynamic psychotherapy in a private psychiatric office: Clinical and cost effectiveness. *American Journal of Psychotherapy*, *56*(2), 225– 232. https://doi.org/10.1176/appi.psychotherapy.2002.56.2.225

*Abbass, Allan A. (2006). Intensive short-term dynamic psychotherapy of treatment-resistant depression: A pilot study. *Depression and Anxiety*, *23*(7), 449–452. https://doi.org/10.1002/da.20203

*Abbass, Allan A., Campbell, S., Magee, K., & Tarzwell, R. (2009). Intensive short-term dynamic psychotherapy to reduce rates of emergency department return visits for patients with medically unexplained symptoms: Preliminary evidence from a prepost intervention study. *Canadian Journal of Emergency Medicine*, *11*(06), 529–534. https://doi.org/10.1017/S1481803500011799

*Abbass, Allan A., Joffres, M. R., & Ogrodniczuk, J. S. (2008). A naturalistic study of intensive short-term dynamic psychotherapy trial therapy. *Brief Treatment and Crisis Intervention*, *8*(2), 164–170. https://doi.org/10.1093/brief-treatment/mhn001

*Abbass, Allan A., Town, J. M., & Bernier, D. C. (2013). Intensive short-term dynamic psychotherapy associated with decreases in electroconvulsive therapy on adult acute care inpatient ward. *Psychotherapy and Psychosomatics*, *82*(6), 406–407. https://doi.org/10.1159/000350576

*Abramowitz, J. S., Franklin, M. E., Schwartz, S. A., & Furr, J. M. (2003). Symptom presentation and outcome of cognitive-behavioral therapy for obsessive-compulsive disorder. *Journal of Consulting and Clinical Psychology*, *71*(6), 1049–1057. https://doi.org/10.1037/0022- 006X.71.6.1049

*Adamson, J., Ali, S., Santhouse, A., Wessely, S., & Chalder, T. (2020). Cognitive behavioural therapy for chronic fatigue and chronic fatigue syndrome: Outcomes from a specialist clinic in the UK. *Journal of the Royal Society of Medicine*, *113*(10), 394–402. https://doi.org/10.1177/0141076820951545

*Andrews, W. P., Wislocki, A. P., Short, F., Chow, D., & Minami, T. (2013). A five-year evaluation of the Human Givens therapy using a practice research network. *Mental Health Review Journal*, *18*(3), 165–176. https://doi.org/10.1108/MHRJ-04-2013-0011

*Andrews, W., Twigg, E., Minami, T., & Johnson, G. (2011). Piloting a practice research network: A 12-month evaluation of the Human Givens approach in primary care at a miscellaneous medical practice. *Psychology & Psychotherapy: Theory, Research & Practice*, *84*(4), 389–405. https://doi.org/10.1111/j.2044-8341.2010.02004.x

*Archer, R., Forbes, Y., Metcalfe, C., & Winter, D. (2000). An investigation of the effectiveness of a voluntary sector psychodynamic counselling service. *The British Journal of Medical Psychology*, *73*(3), 401–412. https://doi.org/10.1348/000711200160499

*Armstrong, J. (2010). How effective are minimally trained/experienced volunteer mental health counsellors? Evaluation of CORE outcome data. *Counselling & Psychotherapy Research*, *10*(1), 22–31. https://doi.org/10.1080/14733140903163284

*Asay, T. P., Lambert, M. J., Gregersen, A. T., & Goates, M. K. (2002). Using patient-focused research in evaluating treatment outcome in private practice. *Journal of Clinical Psychology*, *58*(10), 1213–1225. https://doi.org/10.1002/jclp.10107

*Ashworth, F., Clarke, A., Jones, L., Jennings, C., & Longworth, C. (2015). An exploration of compassion focused therapy following acquired brain injury. *Psychology & Psychotherapy: Theory, Research & Practice*, *88*(2), 143–162. https://doi.org/10.1111/papt.12037

*Askey-Jones, S., David, A. S., Silber, E., Shaw, P., & Chalder, T. (2013). Cognitive behaviour therapy for common mental disorders in people with multiple sclerosis: A benchmarking study. *Behaviour Research and Therapy*, *51*(10), 648–655. https://doi.org/10.1016/j.brat.2013.04.001

*Asnaani, A., Benhamou, K., Kaczkurkin, A. N., Turk-Karan, E., & Foa, E. B. (2020). Beyond the constraints of an RCT: Naturalistic treatment outcomes for anxiety-related disorders. *Behavior Therapy*, *51*(3), 434–446. https://doi.org/10.1016/j.beth.2019.07.007

*Bados, A., Balaguer, G., & Saldaña, C. (2007). Outcome of cognitive-behavioural therapy in training practice with anxiety disorder patients. *British Journal of Clinical Psychology*, *46*(4), 429–435. https://doi.org/10.1348/014466507X209961

*Baldwin, S. A., Berkeljon, A., Atkins, D. C., Olsen, J. A., & Nielsen, S. L. (2009). Rates of change in naturalistic psychotherapy: Contrasting dose-effect and good-enough level models of change. *Journal of Consulting and Clinical Psychology*, *77*(2), 203–211. https://doi.org/10.1037/a0015235

*Bales, D., van Beek, N., Smits, M., Willemsen, S., Busschbach, J. J. V., Verheul, R., & Andrea, H. (2012). Treatment outcome of 18-month, day hospital Mentalization-Based Treatment (MBT) in patients with severe borderline personality disorder in the Netherlands. *Journal of Personality Disorders*, *26*(4), 568–582. https://doi.org/10.1521/pedi.2012.26.4.568

*Ballesteros, F., & Labrador, F. J. (2014). Empirically supported treatments for panic disorder with agoraphobia in a Spanish psychology clinic. *The Spanish Journal of Psychology*, *17*, Article e65. https://doi.org/10.1017/sjp.2014.65

Banham, J. A., & Schweitzer, R. D. A. (2016). Trainee-therapists are not all equal: Examination of therapeutic efficiency, effectiveness and early client dropout after 12 months of clinical training. *Psychology and Psychotherapy: Theory, Research and Practice*, *89*(2), 148–162. https://doi.org/10.1111/papt.12071

*Barkham, M., Margison, F., Leach, C., Lucock, M., Mellor-Clark, J., Evans, C., Benson, L., Connell, J., Audin, K., McGrath, G., & McGrath, M. (2001). Service profiling and outcomes benchmarking using the CORE-OM: Toward practice-based evidence in the psychological therapies. *Journal of Consulting and Clinical Psychology*, *69*(2), 184–196. https://doi.org/10.1037/0022-006X.69.2.184

*Barkham, M., Stiles, W. B., Connell, J., & Mellor-Clark, J. (2012). Psychological treatment outcomes in routine NHS services: What do we mean by treatment effectiveness? *Psychology & Psychotherapy: Theory, Research & Practice*, *85*(1), 1–16. https://doi.org/10.1111/j.2044-8341.2011.02019.x

*Barnicot, K., & Crawford, M. (2019). Dialectical behaviour therapy v. Mentalisation-based therapy for borderline personality disorder. *Psychological Medicine*, *49*(12), 2060–2068. https://doi.org/10.1017/S0033291718002878

*Baronian, R., & Leggett, S. J. (2020). Brief cognitive analytic therapy for adults with chronic pain: A preliminary evaluation of treatment outcome. *British Journal of Pain*, *14*(1), 57–67. https://doi.org/10.1177/2049463719858119

*Bäck, M., Falkenström, F., Gustafsson, S. A., Andersson, G., & Holmqvist, R. (2020). Reduction in depressive symptoms predicts improvement in eating disorder symptoms in interpersonal psychotherapy: Results from a naturalistic study. *Journal of Eating Disorders*, *8*(1), 33. https://doi.org/10.1186/s40337-020-00308-1

*Bäck, M., Gustafsson, S. A., & Holmqvist, R. (2017). Interpersonal psychotherapy for eating disorders with co-morbid depression: A pilot study. *European Journal of Psychotherapy & Counselling*, *19*(4), 378–395. https://doi.org/10.1080/13642537.2017.1386226

*Beail, N., Warden, S., Morsley, K., & Newman, D. (2005). Naturalistic evaluation of the effectiveness of psychodynamic psychotherapy with adults with intellectual disabilities. *Journal of Applied Research in Intellectual Disabilities*, *18*(3), 245–251. https://doi.org/10.1111/j.1468-3148.2005.00223.x

*Beard, C., Stein, A. T., Hearon, B. A., Lee, J., Hsu, K. J., & Björgvinsson, T. (2016). Predictors of depression treatment response in an intensive CBT partial hospital: Predictors of treatment response. *Journal of Clinical Psychology*, *72*(4), 297–310. https://doi.org/10.1002/jclp.22269

*Ben-Porath, D. D., Peterson, G. A., & Smee, J. (2004). Treatment of individuals With borderline personality disorder using dialectical behavior therapy in a community mental health setting: Clinical application and a preliminary investigation. *Cognitive and Behavioral Practice*, *11*(4), 424–434. https://doi.org/10.1016/S1077-7229(04)80059-2

*Birtchnell, J., Denman, C., & Okhai, F. (2004). Cognitive analytic therapy: Comparing two measures of improvement. *Psychology and Psychotherapy: Theory, Research and Practice*, *77*(4), 479–492. https://doi.org/10.1348/1476083042555398

*Bitran, S., SB, M., DA, S., & DH, B. (2008). A pilot study of sensation-focused intensive treatment for panic disorder with moderate to severe agoraphobia: Preliminary outcome and benchmarking data. *Behavior Modification*, *32*(2), 196–214. https://doi.org/10.1177/0145445507309019

*Bjorgvinsson, T., Kertz, S. J., Bigda-Peyton, J. S., Rosmarin, D. H., Aderka, I. M., & Neuhaus, E. C. (2014). Effectiveness of cognitive behavior therapy for severe mood disorders in an acute psychiatric naturalistic setting: A benchmarking study. *Cognitive Behaviour Therapy*, *43*(3), 209–220. https://doi.org/10.1080/16506073.2014.901988

*Blainey, S. H., Rumball, F., Mercer, L., Evans, L. J., & Beck, A. (2017). An evaluation of the effectiveness of psychological therapy in reducing miscellaneous psychological distress for adults with autism spectrum conditions and comorbid mental health problems. *Clinical Psychology & Psychotherapy*, *24*(6), 474–484. https://doi.org/10.1002/cpp.2108

*Boettcher, J., Weinbrecht, A., Heinrich, M., & Renneberg, B. (2019). Treatment of social anxiety disorder and avoidant personality disorder in routine care: A naturalistic study of combined individual and group therapy. *Verhaltenstherapie*, 1–9. https://doi.org/10.1159/000497738

*Bradshaw, W., Roseborough, D., Pahwa, R., & Jordan, J. (2009). Evaluation of psychodynamic psychotherapy in a community mental health center. *The Journal of the American Academy of Psychoanalysis and Dynamic Psychiatry*, *37*(4), 665–681. https://doi.org/10.1521/jaap.2009.37.4.665

*Brand, C. (2020). *A National Evaluation of the Counselling in Primary Care Service (CIPC)* [Thesis]. Trinity College Dublin. School of Psychology. Discipline of Psychology.

*Briggie, A. M., Hilsenroth, M. J., Conway, F., Muran, J. C., & Jackson, J. M. (2016). Patient comfort with audio or video recording of their psychotherapy sessions: Relation to symptomatology, treatment refusal, duration, and outcome. *Professional Psychology: Research and Practice*, *47*(1), 66–76. https://doi.org/10.1037/a0040063

*Brunnbauer, L., Simpson, S., & Balfour, C. (2016). Exploration of client profile and clinical outcome in a university psychology clinic. *Australian Psychologist*, *51*(6), 442–452. https://doi.org/10.1111/ap.12143

*Buckley, J. V., Newman, D. W., Kellett, S., & Beail, N. (2006). A naturalistic comparison of the effectiveness of trainee and qualified clinical psychologists. *Psychology and Psychotherapy: Theory, Research and Practice*, *79*(1), 137–144. https://doi.org/10.1348/147608305X52595

*Budge, S. L., Owen, J. J., Kopta, S. M., Minami, T., Hanson, M. R., & Hirsch, G. (2013). Differences among trainees in client outcomes associated with the phase model of change. *Psychotherapy*, *50*(2), 150–157. https://doi.org/10.1037/a0029565

*Burdett, H., & Greenberg, N. (2019). Service evaluation of a Human Givens Therapy service for veterans. *Occupational Medicine*, *69*(8), 586–592. https://doi.org/10.1093/occmed/kqz045

*Burlingame, G. M., Gleave, R., Erekson, D., Nelson, P. L., Olsen, J., Thayer, S., & Beecher, M. (2016). Differential effectiveness of group, individual, and conjoint treatments: An archival analysis of OQ-45 change trajectories. *Psychotherapy Research*, *26*(5), 556–572. https://doi.org/10.1080/10503307.2015.1044583

*Butler, R. M., O’Day, E. B., Swee, M. B., Horenstein, A., & Heimberg, R. G. (2020). Cognitive behavioral therapy for social anxiety disorder: Predictors of treatment outcome in a quasi- naturalistic setting. *Behavior Therapy*, S000578942030085X. https://doi.org/10.1016/j.beth.2020.06.002

*Byrne, S. M., Fursland, A., Allen, K. L., & Watson, H. (2011). The effectiveness of enhanced cognitive behavioural therapy for eating disorders: An open trial. *Behaviour Research and Therapy*, *49*(4), 219–226. https://doi.org/10.1016/j.brat.2011.01.006

*Cahill, J., Barkham, M., Hardy, G., Rees, A., Shapiro, D. A., Stiles, S., & Macaskill, N. (2003). Outcomes of patients completing and not completing cognitive therapy for depression. *British Journal of Clinical Psychology*, *42*(2), 133–143. https://doi.org/10.1348/014466503321903553

*Carney, C. E., Harris, A. L., Friedman, J., & Segal, Z. V. (2011). Residual sleep beliefs and sleep disturbance following cognitive behavioral therapy for major depression. *Depression and Anxiety*, *28*(6), 464–470. https://doi.org/10.1002/da.20811

*Carr, M. M., Saules, K. K., Koch, E. I., & Waltz, T. J. (2017). Testing the dose-response curve in a training clinic setting: Use of client pretreatment factors to minimize bias in estimates. *Training and Education in Professional Psychology*, *11*(1), 26–32. https://doi.org/10.1037/tep0000135

Carter, M. F. (2005). Time-limited therapy in a Community Mental Health Team setting. *Counselling and Psychotherapy Research*, *5*(1), 43–47. https://doi.org/10.1080/14733140512331343895

*Chase, T., Wetterneck, C. T., Bartsch, R. A., Leonard, R. C., & Riemann, B. C. (2015). Investigating treatment outcomes across OCD symptom dimensions in a clinical sample of OCD patients. *Cognitive Behaviour Therapy*, *44*(5), 365–376. https://doi.org/10.1080/16506073.2015.1015162

*Christiansen, S., Jürgens, T. P., & Klinger, R. (2015). Outpatient combined group and individual cognitive-behavioral treatment for patients with migraine and tension-type headache in a routine clinical setting. *Headache: The Journal of Head & Face Pain*, *55*(8), 1072–1091. https://doi.org/10.1111/head.12626

*Clapp, J. D., Grubaugh, A. L., Allen, J. G., Mahoney, J., Oldham, J. M., Fowler, J. C., Ellis, T., Elhai, J. D., & Frueh, B. C. (2013). Modeling trajectory of depressive symptoms among psychiatric inpatients: A latent growth curve approach. *The Journal of Clinical Psychiatry*, *74*(5), 492–499. https://doi.org/10.4088/JCP.12m07842

*Connell, J., Barkham, M., & Mellor-Clark, J. (2008). The effectiveness of UK student counselling services: An analysis using the CORE System. *British Journal of Guidance & Counselling*, *36*(1), 1–18. https://doi.org/10.1080/03069880701715655

*Cooper, A., Abbass, A., Zed, J., Bedford, L., Sampalli, T., & Town, J. (2017). Implementing a psychotherapy service for medically unexplained symptoms in a primary care setting. *Journal of Clinical Medicine*, *6*(12), 109. https://doi.org/10.3390/jcm6120109

*Daig, I., Klapp, B. F., & Fliege, H. (2009). Narcissism predicts therapy outcome in psychosomatic patients. *Journal of Psychopathology and Behavioral Assessment*, *31*(4), 368–377. https://doi.org/10.1007/s10862-008-9122-0

*Davis, D., Corrin-Pendry, S., & Savill, M. (2008). A follow-up study of the long-term effects of counselling in a primary care counselling psychology service. *Counselling and Psychotherapy Research*, *8*(2), 80–84. https://doi.org/10.1080/14733140802007863

Davis, L., Barlow, B., & Smith, L. (2010). Comorbidity and the treatment of principal anxiety disorders in a naturalistic sample. *Behavior Therapy*, *41*(3), 296–305. https://doi.org/10.1016/j.beth.2009.09.002

*de Jongh, A., Holmshaw, M., Carswell, W., & van Wijk, A. (2011). Usefulness of a trauma- focused treatment approach for travel phobia. *Clinical Psychology & Psychotherapy*, *18*(2), 124–137. https://doi.org/10.1002/cpp.680

*Delgadillo, J., & Gonzalez Salas Duhne, P. (2020). Targeted prescription of cognitive-behavioral therapy versus person-centered counseling for depression using a machine learning approach. *Journal of Consulting and Clinical Psychology*, *88*(1), 14–24. https://doi.org/10.1037/ccp0000476

*Dennhag, I., & Armelius, B.-Å. (2012). Baseline training in cognitive and psychodynamic psychotherapy during a psychologist training program: Exploring client outcomes in therapies of one or two semesters. *Psychotherapy Research*, *22*(5), 515–526. https://doi.org/10.1080/10503307.2012.677332

*Dickson, J. M., & Gullo, M. J. (2015). The role of brief CBT in the treatment of anxiety and depression for young adults at a UK university: A pilot prospective audit study. *The Cognitive Behaviour Therapist*, *8*, Article e14. https://doi.org/10.1017/S1754470X15000240

*Doorn, K. A., Macdonald, J., Stein, M., Cooper, A. M., & Tucker, S. (2014). Experiential Dynamic Therapy: A Preliminary Investigation Into the effectiveness and process of the extended initial session: Extended initial EDT session. *Journal of Clinical Psychology*, *70*(10), 914–923. https://doi.org/10.1002/jclp.22094

*Douglas, A., Ablett-Tate, N., & Chadd, N. (2016). Dynamic interpersonal therapy in an NHS tertiary level specialist psychotherapy service. *Psychoanalytic Psychotherapy*, *30*(3), 223– 239. https://doi.org/10.1080/02668734.2016.1198415

*Ehlers, A., Grey, N., Wild, J., Stott, R., Liness, S., Deale, A., Handley, R., Albert, I., Cullen, D., Hackmann, A., Manley, J., McManus, F., Brady, F., Salkovskis, P., & Clark, D. M. (2013). Implementation of cognitive therapy for PTSD in routine clinical care: Effectiveness and moderators of outcome in a consecutive sample. *Behaviour Research and Therapy*, *51*(11), 742–752. https://doi.org/10.1016/j.brat.2013.08.006

Ellison, W. D., Levy, K. N., Cain, N. M., Ansell, E. B., & Pincus, A. L. (2013). The impact of pathological narcissism on psychotherapy utilization, initial symptom severity, and early- treatment symptom change: A naturalistic investigation. *Journal of Personality Assessment*, *95*(3), 291–300. https://doi.org/10.1080/00223891.2012.742904

*Estupina Puig, F. J., & Labrador Encinas, F. J. (2012). Effectiveness of cognitive-behavioral treatment for major depressive disorder in a university psychology clinic. *The Spanish Journal of Psychology*, *15*(3), 1388–1399.
https://doi.org/10.5209/rev_SJOP .2012.v15.n3.39423

*Evans, L. J., Beck, A., & Burdett, M. (2017). The effect of length, duration, and intensity of psychological therapy on CORE global distress scores. *Psychology and Psychotherapy: Theory, Research and Practice*, *90*(3), 389–400. https://doi.org/10.1111/papt.12120

Falkenstein, M. J., Nota, J. A., Krompinger, J. W., Schreck, M., Garner, L. E., Potluri, S., Van Kirk, N., Ponzini, G., Tifft, E., Brennan, B. P., Mathes, B., Cattie, J., Crosby, J. M., & Elias, J. A. (2019). Empirically-derived response trajectories of intensive residential treatment in obsessive-compulsive disorder: A growth mixture modeling approach. *Journal of Affective Disorders*, *245*, 827–833. https://doi.org/10.1016/j.jad.2018.11.075

*Falkenström, F. (2010). Does psychotherapy for young adults in routine practice show similar results as therapy in randomized clinical trials? *Psychotherapy Research*, *20*(2), 181–192. https://doi.org/10.1080/10503300903170954

*Fizke, E., Mueller, A., & Huber, D. (2017). Psychoanalytic inpatient psychotherapy of depression: Two naturalistic samples throughout the course of a decade. *European Journal of Psychotherapy & Counselling*, *19*(4), 396–414. https://doi.org/10.1080/13642537.2017.1386224

Flo, E., & Chalder, T. (2014). Prevalence and predictors of recovery from chronic fatigue syndrome in a routine clinical practice. *Behaviour Research and Therapy*, *63*, 1–8. https://doi.org/10.1016/j.brat.2014.08.013

*Flygare, O., Andersson, E., Ringberg, H., Hellstadius, A.-C., Edbacken, J., Enander, J., Dahl, M., Aspvall, K., Windh, I., Russell, A., Mataix-Cols, D., & Rück, C. (2020). Adapted cognitive behavior therapy for obsessive-compulsive disorder with co-occurring autism spectrum disorder: A clinical effectiveness study. *Autism: The International Journal of Research and Practice*, *24*(1), 190–199. https://doi.org/10.1177/1362361319856974

*Forand, N. R., Evans, S., Haglin, D., & Fishman, B. (2011). Cognitive behavioral therapy in practice: Treatment delivered by trainees at an outpatient clinic Is clinically effective. *Behavior Therapy*, *42*(4), 612–623. https://doi.org/10.1016/j.beth.2011.02.001

*Fortune, L., Gracey, D., Burke, M., & Rawson, D. (2005). The effect of service setting on treatment outcome: A comparison between cognitive behavioural approaches within primary and secondary care. *Journal of Mental Health*, *14*(5), 483–498. https://doi.org/10.1080/09638230500271162

*Fowler, J. Christopher, Clapp, J. D., Madan, A., Allen, J. G., Frueh, B. C., Fonagy, P., & Oldham, J. M. (2018). A naturalistic longitudinal study of extended inpatient treatment for adults with borderline personality disorder: An examination of treatment response, remission and deterioration. *Journal of Affective Disorders*, *235*, 323–331. https://doi.org/10.1016/j.jad.2017.12.054

*Fowler, J. Christopher, Clapp, J. D., Madan, A., Allen, J. G., Frueh, B. C., & Oldham, J. M. (2017). An open effectiveness trial of a multimodal inpatient treatment for depression and anxiety among adults with serious mental illness. *Psychiatry*, *80*(1), 42–54. https://doi.org/10.1080/00332747.2016.1196072

*Fox, E., Krawczyk, K., Staniford, J., & Dickens, G. L. (2015). A service evaluation of a 1-year Dialectical Behaviour Therapy programme for women with borderline personality disorder in a low secure unit. *Behavioural and Cognitive Psychotherapy*, *43*(6), 676–691. https://doi.org/10.1017/S1352465813001124

*Frueh, B. C., Grubaugh, A. L., Cusack, K. J., Kimble, M. O., Elhai, J. D., & Knapp, R. G. (2009). Exposure-based cognitive-behavioral treatment of PTSD in adults with schizophrenia or schizoaffective disorder: A pilot study. *Journal of Anxiety Disorders*, *23*(5), 665–675. https://doi.org/10.1016/j.janxdis.2009.02.005

*Galili-Weinstock, L., Chen, R., Atzil-Slonim, D., Bar-Kalifa, E., Peri, T., & Rafaeli, E. (2018). The association between self-compassion and treatment outcomes: Session-level and treatment-level effects. *Journal of Clinical Psychology*, *74*(6), 849–866. https://doi.org/10.1002/jclp.22569

*Gamble, S. A. A., Talbot, N. L. L., Cashman-Brown, S. M. M., He, H., Poleshuck, E. L. L., Connors, G. J. J., & Conner, K. R. R. (2013). A pilot study of interpersonal psychotherapy for alcohol-dependent women with co-occurring major depression. *Substance Abuse*, *34*(3), 233–241. https://doi.org/10.1080/08897077.2012.746950

*Ghafoori, B., Wolf, M. G., Nylund-Gibson, K., Felix, E. D., & Wolf, M. G. (2019). A naturalistic study exploring mental health outcomes following trauma-focused treatment among diverse survivors of crime and violence. *Journal of Affective Disorders*, *245*, 617–625. https://doi.org/10.1016/j.jad.2018.11.060

*Ghilardi, A., Buizza, C., Costa, A., & Teodori, C. (2018). A follow-up study on students attending a university counselling service in Northern Italy. *British Journal of Guidance & Counselling*, *46*(4), 456–466. https://doi.org/10.1080/03069885.2017.1391372

*Gibbard, I., & Hanley, T. (2008). A five-year evaluation of the effectiveness of person-centred counselling in routine clinical practice in primary care. *Counselling and Psychotherapy Research*, *8*(4), 215–222. https://doi.org/10.1080/14733140802305440

*Gibbons, C. J., Fournier, J. C., Stirman, S. W., DeRubeis, R. J., Crits-Christoph, P., & Beck, A. T. (2010). The clinical effectiveness of cognitive therapy for depression in an outpatient clinic. *Journal of Affective Disorders*, *125*(1), 169–176. https://doi.org/10.1016/j.jad.2009.12.030

*Gilbert, N., Barkham, M., Richards, A., & Cameron, I. (2005). The effectiveness of a primary care mental health service delivering brief psychological interventions: A benchmarking study using the CORE system. *Primary Care Mental Health*, *3*(4), 241–251.

*Gillespie, K., Duffy, M., Hackmann, A., & Clark, D. M. (2002). Community based cognitive therapy in the treatment of post-traumatic stress disorder following the Omagh bomb. *Behaviour Research and Therapy*, *40*(4), 345–357. https://doi.org/10.1016/S0005- 7967(02)00004-9

*Gimeno-Peon, A., Prado-Abril, J., Inchausti, F., Barrio-Nespereira, A., Alvarez-Casariego, M. T., & Duncan, B. L. (2019). Systematic client feedback: A naturalistic pilot study. *Ansiedad y Estres*, *25*(2), 132–137. https://doi.org/10.1016/j.anyes.2019.04.005

*Goldberg, S. B., Miller, S. D., Nielsen, S. L., Rousmaniere, T., Whipple, J., Hoyt, W. T., & Wampold, B. E. (2016). Do psychotherapists improve with time and experience? A longitudinal analysis of outcomes in a clinical setting. *Journal of Counseling Psychology*, *63*(1), 1–11. https://doi.org/10.1037/cou0000131

*Gordon, R. M. (2001). MMPI/MMPI-2 changes in long-term psychoanalytic psychotherapy. *Issues in Psychoanalytic Psychology*, *23*(1), 59–79.

*Graca, J. J., Palmer, G. A., & Occhietti, K. E. (2014). Psychotherapeutic interventions for symptom reduction in veterans with PTSD: An observational study in a residential clinical setting. *Journal of Loss & Trauma*, *19*(6), 558–567. https://doi.org/10.1080/15325024.2013.810441

*Greasley, P., & Small, N. (2005). Evaluating a primary care counselling service: Outcomes and issues. *Primary Health Care Research and Development*, *6*(2), 125–136. https://doi.org/10.1191/1463423605pc206oa

*Gropalis, M., Bleichhardt, G., Witthöft, M., & Hiller, W. (2012). Hypochondriasis, somatoform disorders, and anxiety disorders: Sociodemographic variables, miscellaneous psychopathology, and naturalistic treatment effects. *Journal of Nervous & Mental Disease*, *200*(5), 406–412. https://doi.org/10.1097/NMD.0b013e31825322e5

*Guthrie, E. (2004). Effectiveness of psychodynamic interpersonal therapy training for primary care counselors. *Psychotherapy Research*, *14*(2), 161–175. https://doi.org/10.1093/ptr/kph015

*Haase, M., Frommer, J., Franke, G.-H., Hoffmann, T., Schulze-Muetzel, J., Jäger, S., Grabe, H.- J., Spitzer, C., & Schmitz, N. (2008). From symptom relief to interpersonal change: Treatment outcome and effectiveness in inpatient psychotherapy. *Psychotherapy Research*, *18*(5), 615–624. https://doi.org/10.1080/10503300802192158

*Hahlweg, K., Fiegenbaum, W., Frank, M., Schroeder, B., & von Witzleben, I. (2001). Short- and long-term effectiveness of an empirically supported treatment for agoraphobia. *Journal of Consulting and Clinical Psychology*, *69*(3), 375–382. https://doi.org/10.1037/0022- 006X.69.3.375

*Halje, K., Timpka, T., Tylestedt, P., Adler, A.-K., Fröberg, L., Schyman, T., Johansson, K., & Dahl, K. (2015). Self-referral psychological treatment centre for young adults: A 2-year observational evaluation of routine practice before and after treatment. *British Medical Journal Open*, *5*(8), Article e008030. https://doi.org/10.1136/bmjopen-2015-008030

*Harnett, P., O’Donovan, A., & Lambert, M. J. (2010). The dose response relationship in psychotherapy: Implications for social policy. *Clinical Psychologist*, *14*(2), 39–44. https://doi.org/10.1080/13284207.2010.500309

*Harte, C. B., & Hawkins, R. C. I. (2016). Impact of personality disorder comorbidity on cognitive-behavioral therapy outcome for mood and anxiety disorders: Results from a university training clinic. *Research in Psychotherapy: Psychopathology, Process and Outcome*, *19*(2). https://doi.org/10.4081/ripppo.2016.210

*Haugen, P. T., Werth, A. S., Foster, A. L., & Owen, J. (2017). Are rupture-repair episodes related to outcome in the treatment of trauma-exposed World Trade Center responders? *Counselling & Psychotherapy Research*, *17*(4), 276–282. https://doi.org/10.1002/capr.12138

*Heins, M. J., Knoop, H., Lobbestael, J., & Bleijenberg, G. (2011). Childhood maltreatment and the response to cognitive behavior therapy for chronic fatigue syndrome. *Journal of Psychosomatic Research*, *71*(6), 404–410. https://doi.org/10.1016/j.jpsychores.2011.05.005

Hill, C. E., Baumann, E., Shafran, N., Gupta, S., Morrison, A., Rojas, A. E. P., Spangler, P. T., Griffin, S., Pappa, L., & Gelso, C. J. (2015). Is training effective? A study of counseling psychology doctoral trainees in a psychodynamic/interpersonal training clinic. *Journal of Counseling Psychology*, *62*(2), 184–201. https://doi.org/10.1037/cou0000053

*Hilsenroth, M. J., Ackerman, S. J., Blagys, M. D., Baity, M. R., & Mooney, M. A. (2003). Short- term psychodynamic psychotherapy for depression: An examination of statistical, clinically significant, and technique-specific change. *The Journal of Nervous and Mental Disease*, *191*(6), 349–357. https://doi.org/10.1097/01.NMD.0000071582.11781.67

*Hiltunen, A. J., Kocys, E., & Perrin-Wallqvist, R. (2013). Effectiveness of cognitive behavioral therapy: An evaluation of therapies provided by trainees at a university psychotherapy training center. *PsyCh Journal*, *2*(2), 101–112. https://doi.org/10.1002/pchj.23

*Hiney-Saunders, K., Ousley, L., Caw, J., Cassinelli, E., & Waller, G. (2019). Effectiveness of treatment for adolescents and adults with anorexia nervosa in a routine residential setting. *Eating Disorders*, *29*(1), 1–15. https://doi.org/10.1080/10640266.2019.1656460

*Hirsch, C., Jolley, S., & Williams, R. (2000). A study of outcome in a clinical psychology service and preliminary evaluation of cognitive-behavioural therapy in real practice. *Journal of Mental Health*, *9*(5), 537–549. https://doi.org/10.1080/09638230020005264

*Hitt, D., Tahir, T., Davies, L., Delahay, J., & Kelson, M. (2018). The clinical effectiveness of a cognitive behavioural therapy intervention in a work setting: A 5-year retrospective analysis of outcomes. *Journal of Research in Nursing*, *23*(4), 360–372. https://doi.org/10.1177/1744987117745580

*Holmqvist, R., Ström, T., & Foldemo, A. (2014). The effects of psychological treatment in primary care in Sweden - A practice-based study. *Nordic Journal of Psychiatry*, *68*(3), 204–212. https://doi.org/10.3109/08039488.2013.797023

*Houghton, S., Saxon, D., Bradburn, M., Ricketts, T., & Hardy, G. (2010). The effectiveness of routinely delivered cognitive behavioural therapy for obsessive-compulsive disorder: A benchmarking study. *British Journal of Clinical Psychology*, *49*(4), 473–489. https://doi.org/10.1348/014466509X475414

*Jakupcak, M., Wagner, A., Paulson, A., Varra, A., & McFall, M. (2010). Behavioral activation as a primary care-based treatment for PTSD and depression among returning veterans. *Journal of Traumatic Stress*, *23*(4), 491–495. https://doi.org/10.1002/jts.20543

Jankowski, P. J., Sandage, S. J., Bell, C. A., Rupert, D., Bronstein, M., & Stavros, G. S. (2019). Latent trajectories of change for clients at a psychodynamic training clinic. *Journal of Clinical Psychology*, *75*(7), 1147–1168. https://doi.org/10.1002/jclp.22769

*Jenkins, P. E., Morgan, C., & Houlihan, C. (2019). Outpatient CBT for underweight patients with eating disorders: Effectiveness within a National Health Service (NHS) eating disorders service. *Behavioural and Cognitive Psychotherapy*, *47*(2), 217–229. https://doi.org/10.1017/S1352465818000449

*Jepsen, E. K. K., Langeland, W., Sexton, H., & Heir, T. (2014). Inpatient treatment for early sexually abused adults: A naturalistic 12-month follow-up study. *Psychological Trauma: Theory, Research, Practice, and Policy*, *6*(2), 142–151. https://doi.org/10.1037/a0031646

*Jepsen, E. K. K., Svagaard, T., Thelle, M. I., McCullough, L., & Martinsen, E. W. (2009). Inpatient treatment for adult survivors of childhood sexual abuse: A preliminary outcome study. *Journal of Trauma & Dissociation*, *10*(3), 315–333. https://doi.org/10.1080/15299730902956812

*Johansson, R., Town, J. M., & Abbass, A. (2014). Davanloo’s intensive short-term dynamic psychotherapy in a tertiary psychotherapy service: Overall effectiveness and association between unlocking the unconscious and outcome. *PeerJ*, *2*, Article e548. https://doi.org/10.7717/peerj.548

*Jolley, S., Garety, P., Peters, E., Fornells-Ambrojo, M., Onwumere, J., Harris, V., Brabban, A., & Johns, L. (2015). Opportunities and challenges in Improving Access to Psychological Therapies for people with Severe Mental Illness (IAPT-SMI): Evaluating the first operational year of the South London and Maudsley (SLaM) demonstration site for psychosis. *Behaviour Research and Therapy*, *64*, 24–30. https://doi.org/10.1016/j.brat.2014.11.006

*Jolley, S., Onwumere, J., Bissoli, S., Bhayani, P., Singh, G., Kuipers, E., Craig, T., & Garety, P. (2015). A pilot evaluation of therapist training in cognitive therapy for psychosis: Therapy quality and clinical outcomes. *Behavioural and Cognitive Psychotherapy*, *43*(4), 478–489. https://doi.org/10.1017/S1352465813001100

Jones, C. (2008). Benchmarking a nurse-led ICU counselling initiative. *Nursing Times*, *104*(38), 32–34.

*Jones, C., Bryant-Waugh, R., Turner, H. M., Gamble, C., Melhuish, L., & Jenkins, P. E. (2012). Who benefits most from guided self-help for binge eating? An investigation into the clinical features of completers and non-completers. *Eating Behaviors*, *13*(2), 146–149. https://doi.org/10.1016/j.eatbeh.2011.11.016

*Jordan, C., Hayee, B., & Chalder, T. (2019). Cognitive behaviour therapy for distress in people with inflammatory bowel disease: A benchmarking study. *Clinical Psychology & Psychotherapy*, *26*(1), 14–23. https://doi.org/10.1002/cpp.2326

*Kalpinski, R. J. (2014). *Client Improvement in a Community-Based Training Clinic: As Indicated by the OQ-45* [Thesis].

*Karlin, B. E., Brown, G. K., Jager-Hyman, S., Green, K. L., Wong, M., Lee, D. S., Bertagnolli, A., & Ross, T. B. (2019). Dissemination and implementation of cognitive behavioral therapy for depression in the kaiser permanente health care system: Evaluation of initial training and clinical outcomes. *Behavior Therapy*, *50*(2), 446–458. https://doi.org/10.1016/j.beth.2018.08.002

*Kehle, S. M. (2008). The effectiveness of cognitive behavioral therapy for miscellaneous ized anxiety disorder in a frontline service setting. *Cognitive Behaviour Therapy*, *37*(3), 1–7. https://doi.org/10.1080/16506070802190262

*Kellett, S., Bennett, D., Ryle, T., & Thake, A. (2013). Cognitive analytic therapy for borderline personality disorder: Therapist competence and therapeutic effectiveness in routine practice. *Clinical Psychology & Psychotherapy*, *20*(3), 216–225. https://doi.org/10.1002/cpp.796

*Kikuchi, H., Niino, M., Hirotani, M., Miyazaki, Y., & Kikuchi, S. (2019). Pilot study on the effects of cognitive behavioral therapy on depression among Japanese patients with multiple sclerosis. *Clinical and Experimental Neuroimmunology*, *10*(3), 180–185. https://doi.org/10.1111/cen3.12529

*Knoop, H., Bleijenberg, G., Gielissen, M. F. M., van der Meer, J. W. M., & White, P. D. (2007). Is a full recovery possible after cognitive behavioural therapy for chronic fatigue syndrome? *Psychotherapy and Psychosomatics*, *76*(3), 171–176. https://doi.org/10.1159/000099844

*Knott, S., Woodward, D., Hoefkens, A., & Limbert, C. (2015). Cognitive behaviour therapy for bulimia nervosa and eating disorders not otherwise specified: Translation from randomized controlled trial to a clinical setting. *Behavioural and Cognitive Psychotherapy*, *43*(6), 641– 654. https://doi.org/10.1017/S1352465814000393

*Kobori, O., Nakazato, M., Yoshinaga, N., Shiraishi, T., Takaoka, K., Nakagawa, A., Iyo, M., & Shimizu, E. (2014). Transporting Cognitive Behavioral Therapy (CBT) and the Improving Access to Psychological Therapies (IAPT) project to Japan: Preliminary observations and service evaluation in Chiba. *Journal of Mental Health Training, Education & Practice*, *9*(3), 155–166. https://doi.org/10.1108/JMHTEP-10-2013-0033

*Kolly, S., Kramer, U., Maillard, P., Charbon, P., Droz, J., Fresard, E., Berney, S., & Despland, J.- N. (2015). Psychotherapy for personality disorders in a natural setting: A pilot study over two years of treatment. *The Journal of Nervous and Mental Disease*, *203*(9), 735–738. https://doi.org/10.1097/NMD.0000000000000356

*Kramer, U., Berthoud, L., Koch, N., Michaud, L., Guex, P., & Despland, J.-N. (2013). Monitoring the effects of adult psychotherapy in routine practice in Switzerland: A feasibility trial. *Counselling & Psychotherapy Research*, *13*(2), 145–150. https://doi.org/10.1080/14733145.2012.729849

*Kvarstein, E. H., Pedersen, G., Urnes, Ø., Hummelen, B., Wilberg, T., & Karterud, S. (2015). Changing from a traditional psychodynamic treatment programme to mentalization-based treatment for patients with borderline personality disorder Does it make a difference? *Psychology and Psychotherapy: Theory, Research and Practice*, *88*(1), 71–86. https://doi.org/10.1111/papt.12036

Lambert, M. J., Hansen, N. B., & Finch, A. E. (2001). Patient-focused research: Using patient outcome data to enhance treatment effects. *Journal of Consulting and Clinical Psychology*, *69*(2), 159–172. https://doi.org/10.1037/0022-006X.69.2.159

*Levitt, J. T., Malta, L. S., Martin, A., Davis, L., & Cloitre, M. (2007). The flexible application of a manualized treatment for PTSD symptoms and functional impairment related to the 9/11 World Trade Center attack. *Behaviour Research and Therapy*, *45*(7), 1419–1433. https://doi.org/10.1016/j.brat.2007.01.004

*Liness, S., Beale, S., Lea, S., Byrne, S., Hirsch, C. R., & Clark, D. M. (2019). Multi-professional IAPT CBT training: Clinical competence and patient outcomes. *Behavioural and Cognitive Psychotherapy*, *47*(6), 672–685. https://doi.org/10.1017/S1352465819000201

*Lopez, M. A., & Basco, M. A. (2015). Effectiveness of cognitive behavioral therapy in public mental health: Comparison to treatment as usual for treatment-resistant depression. *Administration and Policy in Mental Health and Mental Health Services Research*, *42*(1), 87–98. https://doi.org/10.1007/s10488-014-0546-4

*Lopez, M. A., & Basco, M. R. (2011). Feasibility of dissemination of cognitive behavioral therapy to Texas community mental health centers. *The Journal of Behavioral Health Services & Research*, *38*(1), 91–104. https://doi.org/10.1007/s11414-009-9209-8

*LoSavio, S. T., Dillon, K. H., Murphy, R. A., Goetz, K., Houston, F., & Resick, P. A. (2019). Using a learning collaborative model to disseminate cognitive processing therapy to community-based agencies. *Behavior Therapy*, *50*(1), 36–49. https://doi.org/10.1016/j.beth.2018.03.007

*Low, G., Jones, D., Duggan, C., Power, M., & MacLeod, A. (2001). The treatment of deliberate self-harm in bordelrine personality disorder using dialectical behavior therapy: A pilot study in a high security hospital. *Behavioural and Cognitive Psychotherapy*, *29*(1), 85–92. https://doi.org/10.1017/S1352465801001096

*Lu, W., Fite, R., Kim, E., Hyer, L., Yanos, P. T., Mueser, K. T., & Rosenberg, S. D. (2009). Cognitive-behavioral treatment of PTSD in severe mental illness: Pilot study replication in an ethnically diverse population. *American Journal of Psychiatric Rehabilitation*, *12*(1), 73–91. https://doi.org/10.1080/15487760802615863

*Lunnen, K. M., Ogles, B. M., & Pappas, L. N. (2008). A multiperspective comparison of satisfaction, symptomatic change, perceived change, and end-point functioning. *Professional Psychology: Research and Practice*, *39*(2), 145–152. https://doi.org/10.1037/0735-7028.39.2.145

Lutz, W. (2002). Adaptive modeling of progress in outpatient psychotherapy. *Psychotherapy Research*, *12*(4), 427–443. https://doi.org/10.1093/ptr/12.4.427

*Lutz, W., Schiefele, A.-K., Wucherpfennig, F., Rubel, J., & Stulz, N. (2016). Clinical effectiveness of cognitive behavioral therapy for depression in routine care: A propensity score based comparison between randomized controlled trials and clinical practice. *Journal of Affective Disorders*, *189*(1), 150–158. https://doi.org/10.1016/j.jad.2015.08.072

*Marriott, M., & Kellett, S. (2009). Evaluating a cognitive analytic therapy service; practice-based outcomes and comparisons with person-centred and cognitive-behavioural therapies. *Psychology & Psychotherapy: Theory, Research & Practice*, *82*(1), 57–72. https://doi.org/10.1348/147608308X336100

May, J. M. (1984). Number of sessions and psychotherapy outcome: Impact on community mental health center services. *Dissertation Abstracts International*, *45*(5), 1624–1624.

*McAleavey, A. A., Youn, S. J., Xiao, H., Castonguay, L. G., Hayes, J. A., & Locke, B. D. (2019). Effectiveness of routine psychotherapy: Method matters. *Psychotherapy Research : Journal of the Society for Psychotherapy Research*, *29*(2), 139–156. https://doi.org/10.1080/10503307.2017.1395921

*McBride, C., Zuroff, D. C., Ravitz, P., Koestner, R., Moskowitz, D. S., Quilty, L., & Bagby, R. M. (2010). Autonomous and controlled motivation and interpersonal therapy for depression: Moderating role of recurrent depression. *British Journal of Clinical Psychology*, *49*(4), 529–545. https://doi.org/10.1348/014466509X479186

*McDevitt-Petrovic, O., Kirby, K., McBride, O., Shevlin, M., McAteer, D., Gorman, C., & Murphy, J. (2018). Preliminary findings of a new primary and community care psychological service in Northern Ireland: Low-intensity cognitive behavioural therapy for common mental health difficulties. *Behavioural and Cognitive Psychotherapy*, *46*(6), 761– 767. https://doi.org/10.1017/S1352465818000322

*McEvoy, P. M., Burgess, M. M., & Nathan, P. (2014). The relationship between interpersonal problems, therapeutic alliance, and outcomes following group and individual cognitive behaviour therapy. *Journal of Affective Disorders*, *157*, 25–32. https://doi.org/10.1016/j.jad.2013.12.038

*McHugh, P., Gordon, M., & Byrne, M. (2014). Evaluating brief cognitive behavioural therapy within primary care. *Mental Health Review Journal*, *19*(3), 196–206. https://doi.org/10.1108/MHRJ-02-2014-0004

*McHugh, P., Martin, N., Hennessy, M., Collins, P., & Byrne, M. (2016). An evaluation of access to psychological services Ireland: Year one outcomes. *Irish Journal of Psychological Medicine*, *33*(4), 225–233. https://doi.org/10.1017/ipm.2015.51

*McKenzie, N., & Marks, I. (2003). Routine monitoring of outcome over 11 years in a residential behavioural psychotherapy unit. *Psychotherapy and Psychosomatics*, *72*(4), 223–227. https://doi.org/10.1159/000070787

*McLeod, J., Johnston, J., & Griffin, J. (2000). A naturalistic study of the effectiveness of time- limited counselling with low-income clients. *European Journal of Psychotherapy & Counselling*, *3*(2), 263–277. https://doi.org/10.1080/13642530050178159

*Mellor-Clark, J., Twigg, E., Farrell, E., & Kinder, A. (2013). Benchmarking key service quality indicators in UK Employee Assistance Programme Counselling: A CORE System data profile. *Counselling & Psychotherapy Research*, *13*(1), 14–23. https://doi.org/10.1080/14733145.2012.728235

*Merrill, K. A., Tolbert, V. E., & Wade, W. A. (2003). Effectiveness of cognitive therapy for depression in a community mental health center: A benchmarking study. *Journal of Consulting and Clinical Psychology*, *71*(2), 404–409. https://doi.org/10.1037/0022- 006X.71.2.404

*Minami, T., Wampold, B. E., Serlin, R. C., Hamilton, E. G., Brown, G. S. J., & Kircher, J. C. (2008). Benchmarking the effectiveness of psychotherapy treatment for adult depression in a managed care environment: A preliminary study. *Journal of Consulting and Clinical Psychology*, *76*(1), 116–124. https://doi.org/10.1037/0022-006X.76.1.116

*Mitsopoulou, T., Kasvikis, Y., Koumantanou, L., Giaglis, G., Skapinakis, P., & Mavreas, V. (2020). Manualized single-session behavior treatment with self-help manual for panic disorder with or without agoraphobia. *Psychotherapy Research*, *30*(6), 776–787. https://doi.org/10.1080/10503307.2019.1663956

*Moorhead, S., & Scott, J. (1999). Is specialist registrar training in cognitive therapy effective? *Psychiatric Bulletin*, *23*(10), 603–607. https://doi.org/10.1192/pb.23.10.603

*Morley, S., Williams, A., & Hussain, S. (2008). Estimating the clinical effectiveness of cognitive behavioural therapy in the clinic: Evaluation of a CBT informed pain management programme. *Pain*, *137*(3), 670–680. https://doi.org/10.1016/j.pain.2008.02.025

*Mullin, A. S. J., Hilsenroth, M. J., Gold, J., & Farber, B. A. (2017). Changes in object relations over the course of psychodynamic psychotherapy: Changes in object relations. *Clinical Psychology & Psychotherapy*, *24*(2), 501–511. https://doi.org/10.1002/cpp.2021

Murray, A. L., McKenzie, K., Murray, K. R., & Richelieu, M. (2016). An analysis of the effectiveness of university counselling services. *British Journal of Guidance & Counselling*, *44*(1), 130–139. https://doi.org/10.1080/03069885.2015.1043621

*Murray, H. (2017). Evaluation of a Trauma-Focused CBT Training Programme for IAPT services. *Behavioural and Cognitive Psychotherapy*, *45*(5), 467–482. https://doi.org/10.1017/S1352465816000606

*Nordmo, M., Sønderland, N. M., Havik, O. E., Eilertsen, D.-E., Monsen, J. T., & Solbakken, O. A. (2020). Effectiveness of open-ended psychotherapy under clinically representative conditions. *Frontiers in Psychiatry*, *11*, 384. https://doi.org/10.3389/fpsyt.2020.00384

Owen, J., Adelson, J., Budge, S., Wampold, B., Kopta, M., Minami, T., & Miller, S. (2015). Trajectories of change in psychotherapy. *Journal of Clinical Psychology*, *71*(9), 817–827. https://doi.org/10.1002/jclp.22191

*Owen, J., & Hilsenroth, M. J. (2011). Interaction between alliance and technique in predicting patient outcome during psychodynamic psychotherapy. *The Journal of Nervous and Mental Disease*, *199*(6), 384–389. https://doi.org/10.1097/NMD.0b013e31821cd28a

Owen, J., & Hilsenroth, M. J. (2014). Treatment adherence: The importance of therapist flexibility in relation to therapy outcomes. *Journal of Counseling Psychology*, *61*(2), 280–288. https://doi.org/10.1037/a0035753

*Öst, L.-G., Karlstedt, A., & Widén, S. (2012). The effects of cognitive behavior therapy delivered by students in a psychologist training program: An effectiveness study. *Behavior Therapy*, *43*(1), 160–173. https://doi.org/10.1016/j.beth.2011.05.001

*Paine, D. R., Bell, C. A., Sandage, S. J., Rupert, D., Bronstein, M., O’Rourke, C. G., Stavros, G. S., Moon, S. H., & Kehoe, L. E. (2019). Trainee psychotherapy effectiveness at a psychodynamic training clinic: A practice-based study. *Psychoanalytic Psychotherapy*, *33*(1), 20–33. https://doi.org/10.1080/02668734.2019.1582084

*Paley, G., Cahill, J., Barkham, M., Shapiro, D., Jones, J., Patrick, S., & Reid, E. (2008). The effectiveness of psychodynamic-interpersonal therapy (PIT) in routine clinical practice: A benchmarking comparison. *Psychology & Psychotherapy: Theory, Research & Practice*, *81*(2), 157–175. https://doi.org/10.1348/147608307X270889

*Pekarik, G. (1996). Relationship of satisfaction to symptom change, follow-up adjustment, and clinical significance. *Professional Psychology: Research & Practice*, *27*(2), 202–208.

*Pereira, J.-A., Barkham, M., Kellett, S., & Saxon, D. (2017). The role of practitioner resilience and mindfulness in effective practice: A practice-based feasibility study. *Administration and Policy in Mental Health and Mental Health Services Research*, *44*(5), 691–704. https://doi.org/10.1007/s10488-016-0747-0

*Persons, J. B., Burns, D. D., & Perloff, J. M. (1988). Predictors of dropout and outcome in cognitive therapy for depression in a private practice setting. *Cognitive Therapy and Research*, *12*(6), 557–575. https://doi.org/10.1007/BF01205010

*Persons, J. B., Roberts, N. A., Zalecki, C. A., & Brechwald, W. A. G. (2006). Naturalistic outcome of case formulation-driven cognitive-behavior therapy for anxious depressed outpatients. *Behaviour Research and Therapy*, *44*(7), 1041–1051. https://doi.org/10.1016/j.brat.2005.08.005

*Persons, J. B., & Thomas, C. (2019). Symptom severity at week 4 of cognitive-behavior therapy predicts depression remission. *Behavior Therapy*, *50*(4), 791–802. https://doi.org/10.1016/j.beth.2018.12.002

*Pfund, R. A., Peter, S. C., Whelan, J. P., & Meyers, A. W. (2018). When does premature treatment termination occur? Examining session-by-session dropout among clients with gambling disorder. *Journal of Gambling Studies*, *34*(2), 617–630. https://doi.org/10.1007/s10899-017-9733-z

*Plagge, J. M., Lu, M. W., Lovejoy, T. I., Karl, A. I., & Dobscha, S. K. (2013). Treatment of comorbid pain and PTSD in returning veterans: A collaborative approach utilizing behavioral activation. *Pain Medicine*, *14*(8), 1164–1172. https://doi.org/10.1111/pme.12155

*Prout, K. (2016). *An Investigation of Clinically Significant Change among Child and Adolescent Clients of a Graduate-Level Training Clinic* [PhD thesis].

*Puschner, B., Kraft, S., Kächele, H., & Kordy, H. (2007). Course of improvement over 2 years in psychoanalytic and psychodynamic outpatient psychotherapy. *Psychology and Psychotherapy: Theory, Research and Practice*, *80*(1), 51–68. https://doi.org/10.1348/147608306X107593

*Pybis, J., Saxon, D., Hill, A., & Barkham, M. (2017). The comparative effectiveness and efficiency of cognitive behaviour therapy and generic counselling in the treatment of depression: Evidence from the 2nd UK National Audit of psychological therapies. *BMC Psychiatry*, *17*(1), Article 215. https://doi.org/10.1186/s12888-017-1370-7

Quarmby, L., Rimes, K. A., Deale, A., Wessely, S., & Chalder, T. (2007). Cognitive-behaviour therapy for chronic fatigue syndrome: Comparison of outcomes within and outside the confines of a randomised controlled trial. *Behaviour Research and Therapy*, *45*(6), 1085– 1094. https://doi.org/10.1016/j.brat.2006.08.019

*Rauch, S. A. M., Defever, E., Favorite, T., Duroe, A., Garrity, C., Martis, B., & Liberzon, I. (2009). Prolonged exposure for PTSD in a veterans health administration PTSD clinic. *Journal of Traumatic Stress*, *22*(1), 60–64. https://doi.org/10.1002/jts.20380

*Reese, R. J., Duncan, B. L., Bohanske, R. T., Owen, J. J., & Minami, T. (2014). Benchmarking outcomes in a public behavioral health setting: Feedback as a quality improvement strategy. *Journal of Consulting and Clinical Psychology*, *82*(4), 731–742. https://doi.org/10.1037/a0036915

*Reiss, N., Lieb, K., Arntz, A., Shaw, I. A., & Farrell, J. (2014). Responding to the treatment challenge of patients with severe BPD: Results of three pilot studies of inpatient schema therapy. *Behavioural and Cognitive Psychotherapy*, *42*(3), 355–367. https://doi.org/10.1017/S1352465813000027

*Renaud, J., Russell, J. J., & Myhr, G. (2013). The association between positive outcome expectancies and avoidance in predicting the outcome of cognitive behavioural therapy for major depressive disorder. *British Journal of Clinical Psychology*, *52*(1), 42–52. https://doi.org/10.1111/j.2044-8260.2012.02044.x

*Reuber, M., Burness, C., Howlett, S., Brazier, J., & Grünewald, R. (2007). Tailored psychotherapy for patients with functional neurological symptoms: A pilot study. *Journal of Psychosomatic Research*, *63*(6), 625–632. https://doi.org/10.1016/j.jpsychores.2007.06.013

*Reuter, L., Munder, T., Altmann, U., Hartmann, A., Strauss, B., & Scheidt, C. E. (2016). Pretreatment and process predictors of nonresponse at different stages of inpatient psychotherapy. *Psychotherapy Research*, *26*(4), 410–424. https://doi.org/10.1080/10503307.2015.1030471

*Richards, D. A., & Borglin, G. (2011). Implementation of psychological therapies for anxiety and depression in routine practice: Two year prospective cohort study. *Journal of Affective Disorders*, *133*(1-2), 51–60. https://doi.org/10.1016/j.jad.2011.03.024

*Ritschel, L. A., Cheavens, J. S., & Nelson, J. (2012). Dialectical behavior therapy in an intensive outpatient program with a mixed-diagnostic sample. *Journal of Clinical Psychology*, *68*(3), 221–235. https://doi.org/10.1002/jclp.20863

*Rizvi, S. L., Hughes, C. D., Hittman, A. D., & Vieira Oliveira, P. (2017). Can trainees effectively deliver dialectical behavior therapy for individuals with borderline personality disorder? Outcomes from a training clinic. *Journal of Clinical Psychology*, *73*(12), 1599–1611. https://doi.org/10.1002/jclp.22467

*Ronnestad, M. H., Nissen-Lie, H. A., Oddli, H. W., Benum, K., Ekroll, V. B., Gullestad, S. E., Haavind, H., Reichelt, S., Rabu, M., Stanicke, E., von der Lippe, A. L., & Halvorsen, M. S. (2019). Expanding the conceptualization of outcome and clinical effectiveness. *Journal of Contemporary Psychotherapy*, *49*(2), 87–97. https://doi.org/10.1007/s10879-018-9405-z

*Rose, C., & Waller, G. (2017). Cognitive-behavioral therapy for eating disorders in primary care settings: Does it work, and does a greater dose make it more effective? *International Journal of Eating Disorders*, *50*(12), 1350–1355. https://doi.org/10.1002/eat.22778

*Roseborough, D. J. (2006). Psychodynamic psychotherapy: An effectiveness study. *Research on Social Work Practice*, *16*(2), 166–175. https://doi.org/10.1177/1049731505281373

*Rosenberg, S. D., Mueser, K. T., Jankowski, M. K., Salyers, M. P., & Acker, K. (2004). Cognitive-behavioral treatment of PTSD in severe mental illness: Results of a pilot study. *American Journal of Psychiatric Rehabilitation*, *7*(2), 171–186. https://doi.org/10.1080/15487760490476200

*Rothbaum, B. O., & Shahar, F. (2000). Behavioral treatment of obsessive-compulsive disorder in a naturalistic setting. *Cognitive and Behavioral Practice*, *7*(3), 262–270. https://doi.org/10.1016/S1077-7229(00)80082-6

*Ryle, A., & Golynkina, K. (2000). Effectiveness of time-limited cognitive analytic therapy of borderline personality disorder: Factors associated with outcome. *British Journal of Medical Psychology*, *73*(2), 197–210. https://doi.org/10.1348/000711200160426

*Sadock, E., Auerbach, S. M., Rybarczyk, B., & Aggarwal, A. (2014). Evaluation of integrated psychological services in a University-based primary care clinic. *Journal of Clinical Psychology in Medical Settings*, *21*(1), 19–32. https://doi.org/10.1007/s10880-013-9378-8

*Samstag, L. W., & Norlander, K. (2019). Characteristics of trainees’ early sessions: A naturalistic process-outcome study tribute to Jeremy D. Safran. *Psychoanalytic Psychology*, *36*(2), 148–158. https://doi.org/10.1037/pap0000239

*Sanders, P. W., Richards, P. S., McBride, J. A., Lea, T., Hardman, R. K., & Barnes, D. V. (2015). Processes and outcomes of theistic spiritually oriented psychotherapy: A practice-based evidence investigation. *Spirituality in Clinical Practice*, *2*(3), 180–190. https://doi.org/10.1037/scp0000083

*Sauer-Zavala, S., Ametaj, A. A., Wilner, J. G., Bentley, K. H., Marquez, S., Patrick, K. A., Starks, B., Shtasel, D., & Marques, L. (2019). Evaluating transdiagnostic, evidence-based mental health care in a safety-net setting serving homeless individuals. *Psychotherapy*, *56*(1), 100–114. https://doi.org/10.1037/pst0000187

*Särnholm, J., Skúladóttir, H., Rück, C., Pedersen, S. S., Braunschweig, F., & Ljótsson, B. (2017). Exposure-based therapy for symptom preoccupation in atrial fibrillation: An uncontrolled pilot study. *Behavior Therapy*, *48*(6), 808–819. https://doi.org/10.1016/j.beth.2017.06.001

*Scheeres, K., Wensing, M., Knoop, H., & Bleijenberg, G. (2008). Implementing cognitive behavioral therapy for chronic fatigue syndrome in a mental health center: A benchmarking evaluation. *Journal of Consulting and Clinical Psychology*, *76*(1), 163–171. https://doi.org/10.1037/0022-006X.76.1.163

*Schindler, A. C., Hiller, W., & Witthöft, M. (2011). Benchmarking of cognitive-behavioral therapy for depression in efficacy and effectiveness studiesHow do exclusion criteria affect treatment outcome? *Psychotherapy Research*, *21*(6), 644–657. https://doi.org/10.1080/10503307.2011.602750

*Schnicker, K., Hiller, W., & Legenbauer, T. (2013). Drop-out and treatment outcome of outpatient cognitive-behavioral therapy for anorexia nervosa and bulimia nervosa. *Comprehensive Psychiatry*, *54*(7), 812–823. https://doi.org/10.1016/j.comppsych.2013.02.007

*Schulz, P. M., Resick, P. A., Huber, L. C., & Griffin, M. G. (2006). The effectiveness of cognitive processing therapy for PTSD with refugees in a community setting. *Cognitive and Behavioral Practice*, *13*(4), 322–331. https://doi.org/10.1016/j.cbpra.2006.04.011

*Schwartz, C. (2017). *Development and evaluation of behavioral activation guided self-help treatment for mild to moderate depression* [PhD thesis, Rutgers University - Graduate School of Applied; Professional Psychology]. https://doi.org/10.7282/T3MC9321

*Sembill, A., Vocks, S., Kosfelder, J., & Schöttke, H. (2019). The phase model of psychotherapy outcome: Domain-specific trajectories of change in outpatient treatment. *Psychotherapy Research*, *29*(4), 541–552. https://doi.org/10.1080/10503307.2017.1405170

*Shepherd, M., Ashworth, M., Evans, C., SI, R., Rendall, M., & Ward, S. (2005). What factors are associated with improvement after brief psychological interventions in primary care? Issues arising from using routine outcome measurement to inform clinical practice. *Counselling & Psychotherapy Research*, *5*(4), 273–280. https://doi.org/10.1080/14733140600571326

*Signorini, R., Sheffield, J., Rhodes, N., Fleming, C., & Ward, W. (2018). The effectiveness of Enhanced Cognitive Behavioural Therapy (CBT-E): A naturalistic study within an out- patient eating disorder service. *Behavioural and Cognitive Psychotherapy*, *46*(1), 21–34. https://doi.org/10.1017/S1352465817000352

*Simons, A. D., Padesky, C. A., Montemarano, J., Lewis, C. C., Murakami, J., Lamb, K., DeVinney, S., Reid, M., Smith, D. A., & Beck, A. T. (2010). Training and dissemination of cognitive behavior therapy for depression in adults: A preliminary examination of therapist competence and client outcomes. *Journal of Consulting and Clinical Psychology*, *78*(5), 751–756. https://doi.org/10.1037/a0020569

Simpson, S., Guerrini, L., & Rochford, S. (2015). Telepsychology in a University psychology clinic setting: A pilot Ppoject. *Australian Psychologist*, *50*(4), 285–291. https://doi.org/10.1111/ap.12131

*Slavin-Mulford, J., Hilsenroth, M., Weinberger, J., & Gold, J. (2011). Therapeutic interventions related to outcome in psychodynamic psychotherapy for anxiety disorder patients. *The Journal of Nervous and Mental Disease*, *199*(4), 214–221. https://doi.org/10.1097/NMD.0b013e3182125d60

Smith, D. P., Fairweather-Schmidt, A. K., Harvey, P. W., & Battersby, M. W. (2018). How does routinely delivered cognitive-behavioural therapy for gambling disorder compare to "gold standard" clinical trial? *Clinical Psychology & Psychotherapy*, *25*(2), 302–310. https://doi.org/10.1002/cpp.2163

*Smout, M. F., Harris, J. K., & Furber, G. (2019). Outcome benchmarks for cognitive behaviour therapy delivered by student psychologist training clinics. *Australian Psychologist*, *54*(4), 272–291. https://doi.org//10.1111/ap.12387

*Steinert, C., Klein, S., Leweke, F., & Leichsenring, F. (2015). Do personality traits predict outcome of psychodynamically oriented psychosomatic inpatient treatment beyond initial symptoms? *British Journal of Clinical Psychology*, *54*(1), 109–125. https://doi.org/10.1111/bjc.12064

*Steinert, C., Kruse, J., Leweke, F., & Leichsenring, F. (2019). Psychosomatic inpatient treatment: Real-world effectiveness, response rates and the helping alliance. *Journal of Psychosomatic Research*, *124*, Article 109743. https://doi.org/10.1016/j.jpsychores.2019.109743

*Stiles, W. B., Barkham, M., & Wheeler, S. (2015). Duration of psychological therapy: Relation to recovery and improvement rates in UK routine practice. [corrected]. *The British Journal of Psychiatry : The Journal of Mental Science*, *207*(2), 115–122. https://doi.org/10.1192/bjp.bp.114.145565

*Stiles, W. B., Leach, C., Barkham, M., Lucock, M., Iveson, S., Shapiro, D. A., Iveson, M., & Hardy, G. E. (2003). Early sudden gains in psychotherapy under routine clinic conditions: Practice-based evidence. *Journal of Consulting and Clinical Psychology*, *71*(1), 14–21.

*Strepparava, M. G., Bani, M., Zorzi, F., Corrias, D., Dolce, R., & Rezzonico, G. (2016). Cognitive counselling intervention: Treatment effectiveness in an Italian university centre. *British Journal of Guidance & Counselling*, *44*(4), 423–433. https://doi.org/10.1080/03069885.2015.1110561

*Stulz, N., Lutz, W., Kopta, S. M., Minami, T., & Saunders, S. M. (2013). Dose effect relationship in routine outpatient psychotherapy: Does treatment duration matter? *Journal of Counseling Psychology*, *60*(4), 593–600. https://doi.org/10.1037/a0033589

*Swift, J. K., & Callahan, J. L. (2010). A comparison of client preferences for intervention empirical support versus common therapy variables. *Journal of Clinical Psychology*, *66*(12), 1217–1231. https://doi.org/10.1002/jclp.20720

*Talbot, N. L., Conwell, Y., O’Hara, M. W., Stuart, S., Ward, E. A., Gamble, S. A., Watts, A., & Tu, X. (2005). Interpersonal psychotherapy for depressed women with sexual abuse histories: A pilot study in a community mental health center. *The Journal of Nervous and Mental Disease*, *193*(12), 847–850. https://doi.org/10.1097/01.nmd.0000188987.07734.22

*Timmons, K. (2013). Assessing the effectiveness of modular psychotherapy in a community clinic. *Dissertation Abstracts International: Section B: The Sciences and Engineering*, *74*(2), No–Specified.

*Trockel, M., Karlin, B. E., Taylor, C. B., Brown, G. K., & Manber, R. (2015). Effects of cognitive behavioral therapy for insomnia on suicidal ideation in veterans. *Sleep*, *38*(2), 259–265. https://doi.org/10.5665/sleep.4410

*Turner, H., Marshall, E., Stopa, L., & Waller, G. (2015). Cognitive-behavioural therapy for outpatients with eating disorders: Effectiveness for a transdiagnostic group in a routine clinical setting. *Behaviour Research and Therapy*, *68*, 70–75. https://doi.org/10.1016/j.brat.2015.03.001

*Tuschen-Caffier, B., Pook, M., & Frank, M. (2001). Evaluation of manual-based cognitive- behavioral therapy for bulimia nervosa in a service setting. *Behaviour Research and Therapy*, *39*(3), 299–308. https://doi.org/10.1016/S0005-7967(00)00004-8

*van der Lem, R., van der Wee, N. J. A., van Veen, T., & Zitman, F. G. (2012). Efficacy versus effectiveness: A direct comparison of the outcome of treatment for mild to moderate depression in randomized controlled trials and daily practice. *Psychotherapy and Psychosomatics*, *81*(4), 226–234. https://doi.org/10.1159/000330890

van Rijn, B., Sills, C., Hunt, J., Shivanath, S., Gildebrand, K., & Fowlie, H. (2008). Developing clinical effectiveness in psychotherapy training: Action research. *Counselling & Psychotherapy Research*, *8*(4), 261–268. https://doi.org/10.1080/14733140802305804

*van Rijn, B., Wild, C., & Dumitru, A. (2014). Challenges to developing routine outcomes evaluation in different practice settings and cultures: A naturalistic enquiry in Spain and the UK. *International Journal of Transactional Analysis Research & Practice*, *5*(2). https://doi.org/10.29044/v5i2p28

*van Woudenberg, C., Voorendonk, E. M., Bongaerts, H., Zoet, H. A., Verhagen, M., Lee, C. W., van Minnen, A., & De Jongh, A. (2018). Effectiveness of an intensive treatment programme combining prolonged exposure and eye movement desensitization and reprocessing for severe post-traumatic stress disorder. *European Journal of Psychotraumatology*, *9*(1), 1487225. https://doi.org/10.1080/20008198.2018.1487225

Vermote, R., Fonagy, P., Vertommen, H., Verhaest, Y., Stroobants, R., Vandeneede, B., Corveleyn, J., Lowyck, B., Luyten, P., & Peuskens, J. (2009). Outcome and outcome trajectories of personality disordered patients during and after a psychoanalytic hospitalization-based treatment. *Journal of Personality Disorders*, *23*(3), 294–307. https://doi.org/10.1521/pedi.2009.23.3.294

*Vescovelli, F., Melani, P., Ruini, C., Ricci Bitti, P. E., & Monti, F. (2017). University counseling service for improving students’ mental health. *Psychological Services*, *14*(4), 470–480. https://doi.org/10.1037/ser0000166

*von Brachel, R., Hirschfeld, G., Berner, A., Willutzki, U., Teismann, T., Cwik, J. C., Velten, J., Schulte, D., & Margraf, J. (2019). Long-term effectiveness of cognitive behavioral therapy in routine outpatient care: A 5- to 20-year follow-up study. *Psychotherapy and Psychosomatics*, *88*(4), 225–235. https://doi.org/10.1159/000500188

*Waller, G., Gray, E., Hinrichsen, H., Mountford, V., Lawson, R., & Patient, E. (2014). Cognitive- behavioral therapy for bulimia nervosa and atypical bulimic nervosa: Effectiveness in clinical settings: CBT For bulimic disorders. *International Journal of Eating Disorders*, *47*(1), 13–17. https://doi.org/10.1002/eat.22181

*Waller, G., Tatham, M., Turner, H., Mountford, V. A., Bennetts, A., Bramwell, K., Dodd, J., & Ingram, L. (2018). A 10-session cognitive-behavioral therapy (CBT-T) for eating disorders: Outcomes from a case series of nonunderweight adult patients. *International Journal of Eating Disorders*, *51*(3), 262–269. https://doi.org/10.1002/eat.22837

*Waller, H., Garety, G., Jolley, S., Fornells-Ambrojo, M., Kuipers, E., Onwumere, J., Woodall, A., Emsley, R., Craig, T., Waller, H., Garety, P. A., Jolley, S., Fornells-Ambrojo, M., Kuipers, E., Onwumere, J., Woodall, A., Emsley, R., & Craig, T. (2013). Low intensity cognitive behavioural therapy for psychosis: A pilot study. *Journal of Behavior Therapy & Experimental Psychiatry*, *44*(1), 98–104. https://doi.org/10.1016/j.jbtep.2012.07.013

*Walser, R. D., Garvert, D. W., Karlin, B. E., Trockel, M., Ryu, D. M., & Taylor, C. B. (2015). Effectiveness of acceptance and commitment therapy in treating depression and suicidal ideation in veterans. *Behaviour Research and Therapy*, *74*, 25–31. https://doi.org/10.1016/j.brat.2015.08.012

*Warren, R., & Thomas, J. C. (2001). Cognitive behavior therapy of obsessive compulsive disorder in private practice: An effectiveness study. *Journal of Anxiety Disorders*, *15*(4), 277–285. https://doi.org/10.1016/S0887-6185(01)00063-9

Wattar, U., Sorensen, P., Buemann, I., Birket-Smith, M., Salkovskis, P. M., Albertsen, M., & Strange, S. (2005). Outcome of cognitive-behavioural treatment for health anxiety (hypochondriasis) in a routine clinical setting. *Behavioural and Cognitive Psychotherapy*, *33*(2), 165–175. https://doi.org/10.1017/S1352465804002000

*Werbart, A., Forsström, D., & Jeanneau, M. (2012). Long-term outcomes of psychodynamic residential treatment for severely disturbed young adults: A naturalistic study at a Swedish therapeutic community. *Nordic Journal of Psychiatry*, *66*(6), 367–375. https://doi.org/10.3109/08039488.2012.654508

*Westbrook, D., & Kirk, J. (2005). The clinical effectiveness of cognitive behaviour therapy: Outcome for a large sample of adults treated in routine practice. *Behaviour Research and Therapy*, *43*(10), 1243–1261. https://doi.org/10.1016/j.brat.2004.09.006

*Wilkinson, C., von Linden, M., Wacha-Montes, A., Bryan, C., & O’Leary, K. (2017). Cognitive processing therapy for post-traumatic stress disorder in a University Counselling Center: An outcome study. *The Cognitive Behaviour Therapist*, *10*(1), 1–11. https://doi.org/10.4088/JCP .13m08842

*Winter, D., Archer, R., Spearman, P., Costello, M., Quaite, A., & Metcalfe, C. (2003). Explorations of the effectiveness of a voluntary sector psychodynamic counselling service. *Counselling and Psychotherapy Research*, *3*(4), 261–269. https://doi.org/10.1080/14733140312331384253

*Wiseman, H., & Tishby, O. (2014). Client attachment, attachment to the therapist and client- therapist attachment match: How do they relate to change in psychodynamic psychotherapy? *Psychotherapy Research*, *24*(3), 392–406. https://doi.org/10.1080/10503307.2014.892646

*Wolf, G. K., Mauntel, G. J., Kretzmer, T., Crawford, E., Thors, C., Strom, T. Q., & Vanderploeg, R. D. (2018). Comorbid posttraumatic stress disorder and traumatic brain injury: Miscellaneous ization of prolonged-exposure PTSD treatment outcomes to postconcussive symptoms, cognition, and self-efficacy in veterans and active duty service members. *Journal of Head Trauma Rehabilitation*, *33*(2), 53–63. https://doi.org/10.1097/HTR.0000000000000344

*Worm-Smeitink, M., Nikolaus, S., Goldsmith, K., Wiborg, J., Ali, S., Knoop, H., & Chalder, T. (2016). Cognitive behaviour therapy for chronic fatigue syndrome: Differences in treatment outcome between a tertiary treatment centre in the United Kingdom and the Netherlands. *Journal of Psychosomatic Research*, *87*, 43–49. https://doi.org/10.1016/j.jpsychores.2016.06.006

*Wright, D., & Abrahams, D. (2015). An investigation into the effectiveness of Dynamic Interpersonal Therapy (DIT) as a treatment for depression and anxiety in IAPT (Increasing Access to Psychological Therapies). *Psychoanalytic Psychotherapy*, *29*(2), 160–170. https://doi.org/10.1080/02668734.2015.1035740

*Ybrandt, H., Berglund, K., Strid, C., Kivi, M., & Knutsson, J. (n.d.). Clinical outcomes in the routine evaluation of psychotherapy given by trainees: Effects on clients’ inter-personal problems and psychological symptoms. *International Journal of Psychotherapy*, *23*(1), 1– 16.

*Young, A., Rogers, K., Davies, L., Pilling, M., Lovell, K., Pilling, S., Belk, R., Shields, G., Dodds, C., Campbell, M., Nassimi-Green, C., Buck, D., & Oram, R. (2017). Evaluating the effectiveness and cost-effectiveness of British Sign Language Improving Access to Psychological Therapies: An exploratory study. *Health Services and Delivery Research*, *5*(24), 1–196. https://doi.org/10.3310/hsdr05240

*Zeeck, A., von Wietersheim, J., Weiß, H., Eduard Scheidt, C., Völker, A., Helesic, A., Eckhardt- Henn, A., Beutel, M., Endorf, K., Knoblauch, J., Rochlitz, P., & Hartmann, A. (2015). Symptom course in inpatient and day clinic treatment of depression: Results from the INDDEP-Study. *Journal of Affective Disorders*, *187*, 35–44. https://doi.org/10.1016/j.jad.2015.07.025

*Zieve, G. G., Persons, J. B., & Yu, L. A. D. (2019). The relationship between dropout and outcome in naturalistic bognitive behavior therapy. *Behavior Therapy*, *50*(1), 189–199. https://doi.org/10.1016/j.beth.2018.05.004
